# High level of complexity and global diversity of the 3q29 locus revealed by optical mapping and long-read sequencing

**DOI:** 10.1186/s13073-023-01184-5

**Published:** 2023-05-10

**Authors:** Feyza Yilmaz, Umamaheswaran Gurusamy, Trenell J. Mosley, Pille Hallast, Kwondo Kim, Yulia Mostovoy, Ryan H. Purcell, Tamim H. Shaikh, Michael E. Zwick, Pui-Yan Kwok, Charles Lee, Jennifer G. Mulle

**Affiliations:** 1 The Jackson Laboratory for Genomic Medicine, 10 Discovery Drive, Farmington, CT 06032 USA; 2 Cardiovascular Research Institute and Institute for Human Genetics, UCSF School of Medicine, 513 Parnassus Ave, San Francisco, CA 94143 USA; 3 Graduate Program in Genetics and Molecular Biology, Laney Graduate School, Emory University, 201 Dowman Drive, Atlanta, GA 30322 USA; 4 Laboratory of Translational Cell Biology, Department of Cell Biology, Emory University School of Medicine, 100 Woodruff Circle, Atlanta, GA 30322 USA; 5 Department of Pediatrics, Section of Genetics and Metabolism, University of Colorado School of Medicine, 13123 E 16Th Ave, Aurora, CO 80045 USA; 6 Department of Genetics, Rutgers University–New Brunswick, Rutgers University, Piscataway, New Brunswick, NJ 08901 USA; 7 Department of Dermatology, UCSF School of Medicine, 1701 Divisadero Street, San Francisco, CA 94115 USA; 8 Department of Psychiatry, Robert Wood Johnson Medical School, Rutgers Biomedical and Health Sciences, Rutgers University, 671 Hoes Lane, New Brunswick, NJ 08901 USA

**Keywords:** 3q29, Structural variations, Genomic disorders, Schizophrenia, NAHR, Copy number variant(s)

## Abstract

**Background:**

High sequence identity between segmental duplications (SDs) can facilitate copy number variants (CNVs) via non-allelic homologous recombination (NAHR). These CNVs are one of the fundamental causes of genomic disorders such as the 3q29 deletion syndrome (del3q29S). There are 21 protein-coding genes lost or gained as a result of such recurrent 1.6-Mbp deletions or duplications, respectively, in the 3q29 locus. While NAHR plays a role in CNV occurrence, the factors that increase the risk of NAHR at this particular locus are not well understood.

**Methods:**

We employed an optical genome mapping technique to characterize the 3q29 locus in 161 unaffected individuals, 16 probands with del3q29S and their parents, and 2 probands with the 3q29 duplication syndrome (dup3q29S). Long-read sequencing-based haplotype resolved de novo assemblies from 44 unaffected individuals, and 1 trio was used for orthogonal validation of haplotypes and deletion breakpoints.

**Results:**

In total, we discovered 34 haplotypes, of which 19 were novel haplotypes. Among these 19 novel haplotypes, 18 were detected in unaffected individuals, while 1 novel haplotype was detected on the parent-of-origin chromosome of a proband with the del3q29S. Phased assemblies from 44 unaffected individuals enabled the orthogonal validation of 20 haplotypes. In 89% (16/18) of the probands, breakpoints were confined to paralogous copies of a 20-kbp segment within the 3q29 SDs. In one del3q29S proband, the breakpoint was confined to a 374-bp region using long-read sequencing. Furthermore, we categorized del3q29S cases into three classes and dup3q29S cases into two classes based on breakpoints. Finally, we found no evidence of inversions in parent-of-origin chromosomes.

**Conclusions:**

We have generated the most comprehensive haplotype map for the 3q29 locus using unaffected individuals, probands with del3q29S or dup3q29S, and available parents, and also determined the deletion breakpoint to be within a 374-bp region in one proband with del3q29S. These results should provide a better understanding of the underlying genetic architecture that contributes to the etiology of del3q29S and dup3q29S.

**Supplementary Information:**

The online version contains supplementary material available at 10.1186/s13073-023-01184-5.

## Background

Genomic disorders account for a substantial fraction of physical, neurodevelopmental, and psychiatric morbidity [1–3]. Examples include deletions at the 7q11.23 locus known as Williams–Beuren syndrome (WBS, OMIM 194,050), deletions at the 22q11.2 locus that give rise to 22q11.2 deletion syndrome (OMIM 611,867), reciprocal pathogenic deletions and duplications at the 16p11.2 locus (OMIM 611,913 and OMIM 614,671), and the 3q29 deletion syndrome (del3q29S) (OMIM 609,425) [4–9]. In the recurrent 3q29 deletion (del 3q29) and duplication (dup 3q29) cases, breakpoints occur within two segmental duplication (SD) blocks that comprise different SD segments with > 98% sequence identity and are therefore prone to non-allelic homologous recombination (NAHR) [10, 11]. It is often difficult to accurately identify the breakpoints that occurred in these types of genomic disorders. Techniques such as short-read sequencing have a limited ability to pinpoint breakpoint locations within repetitive genomic elements like SDs because of the high sequence identity and lengths of the paralogous copies [12]. Long-read sequencing (LRS) and optical genome mapping (OGM), two complementary genomic technologies, are now available, allowing us to interrogate these loci and pinpoint breakpoint locations with increased sequence resolution [13, 14]. These technologies deliver haplotype-resolved de novo assembled genomes and provide the ability to resolve complex regions of the genome even when there is the presence of a substantial amount of SDs.

The 3q29 deletion syndrome was first identified by Rossi and colleagues in 2001 as a cryptic subtelomeric deletion, and later Willatt and colleagues described an additional six individuals with del 3q29 [7, 15]. In 2008, 14,698 individuals with idiopathic mental retardation were screened by array comparative genomic hybridization (aCGH), and reciprocal dup 3q29s were identified in 19 samples [10]. A population prevalence estimate for the del3q29S and duplication syndrome (dup3q29S) is 1 in 30,000 [16] and 1 in 8000–75,000 [17], respectively. More than 200 cases have been reported in the literature, via case reports as well as systematic ascertainment of cases through the 3q29 registry [18–21]. Patient phenotypes include developmental delay, intellectual disability, autism spectrum disorder (ASD), anxiety disorders, attention-deficit/hyperactivity disorder (ADHD), congenital heart defects, and additional neurodevelopmental phenotypes as well as schizophrenia in 30 patients [17, 19, 20, 22]. Similar phenotypes are observed in individuals with the reciprocal dup3q29S [10, 17, 22–30].

A small number (7%) of del 3q29s are inherited from a parent who displays mild phenotypic effects [18, 20]. Most del 3q29 patients typically carry the same ~ 1.6-Mbp de novo recurrent deletion at the 3q29 locus that is absent in both parental genomes. These recurrent de novo rearrangements suggest that the duplication architecture flanking the 3q29 interval predisposes the locus to deletions and duplications through NAHR. It has been noted that complex genomic structure within these regions predisposes individuals to recurrent deletions and duplications associated with genomic disorder [31]. However, in probands with del3q29S or dup3q29S, the parental 3q29 locus and haplotype architecture have not been well characterized, and it is unclear whether distinct duplication configurations can predispose individuals and their progeny to this genomic disorder.

In this study, we first characterized the 3q29 locus in unaffected individuals from the 1000 Genomes Project (1000GP) and the California Initiative to Advance Precision Medicine (CIAPM). Our aim was to identify the haplotypes present at this locus in order to gain a better understanding of their haplotype diversity and frequency across diverse populations using Bionano Genomics (BG) OGM [32, 33]. We then used haplotype-resolved phased assemblies (PA) for orthogonal validation of haplotype structures. Subsequently, we used this information to accurately identify the haplotype structures (and breakpoints) in probands with del3q29S and dup3q29S and determine the parental origin of the deletion [19]. Using PacBio HiFi sequencing on a trio, we further refined the breakpoints in one proband with del3q29S. Our combined data provide a more thorough understanding of the molecular etiology of del3q29S and dup3q29S.

## Methods

### 3q29 segments

In this study, we analyzed a ~ 2-Mbp region on chromosome 3 (GRCh38; 195,428,934–197,230,596), including three SD blocks, denoted as SDA, SDB, and SDC. The 3q29 SD blocks and the segments within them, depicted by colored arrows, were determined based on the labeling pattern from the OGM data and, as previously described [34], the sequence identity between 3q29 SD blocks using blastn (BLASTN 2.9.0 +) [35, 36] (see Additional file 1: Supplementary Methods; Additional file 2: Tables S1, S2). We identified three copies of ~ 26-kbp segment (magenta), four copies of ~ 5 kbp (blue), three copies of ~ 11 kbp (yellow), two copies of ~ 4 kbp (red), one copy of ~ 33 kbp (maroon), one copy of ~ 15 kbp (orange), and one copy of ~ 124 kbp (green) segments within these three SD blocks. The two SD blocks closest to the telomere (SDB and SDC) flank the canonical ~ 1.6-Mbp interval, which is deleted in del3q29S and duplicated in dup3q29S.

### Sample collection—1000 Genomes Project and California Initiative to Advance Precision Medicine samples

Unaffected individuals (*n* = 161 from 26 diverse populations) were part of the 1000GP and CIAPM cohorts and consisted of Africans (AFR) (*n* = 37), Americans (AMR) (*n* = 52), East Asians (EAS) (*n* = 22), Europeans (EUR) (*n* = 27), and South Asians (SAS) (*n* = 23). Genomic DNAs from these individuals were used for OGM (Additional file 1: Figure S1; Additional file 2: Table S3). CIAPM samples were collected as described previously [37]. CIAPM samples and 114 unaffected individuals from 1000GP were designated as the University of California San Francisco (UCSF) dataset. Samples from two publicly available datasets, the Human Genome Structural Variation Consortium (HGSVC) [34] and the Human Pangenome Reference Consortium (HPRC) [38], were included in the analyses. HGSVC included BG OGM data from three samples (HG01573, HG02018, and GM19036), which were previously not studied. Cell lines of these additional samples were obtained from Coriell and maintained in RPMI 1640 media (Gibco Life Technologies) with 15% FBS (Sigma), supplemented with L-glutamine and penicillin/streptomycin, at 37 °C and 5% CO_2_, as previously described [34]. Forty-four unaffected individuals had OGM data and PA available through HPRC, two of which (HG00733 and NA19240) were shared between HGSVC and HPRC. Three samples from this dataset (HG00513, HG00732, and NA19239) were previously studied as part of HGSVC [34] and not included.

### Sample collection—The 3q29 Project samples

Subjects (*n* = 46) were recruited from the 3q29 Project registry [19] (3q29deletion.org) as previously described (Additional file 2: Table S3) [39]. Inclusion criteria were (a) a validated clinical diagnosis of del3q29S where the deletion of the subject overlapped the canonical region (chr3:195,725,000–197,350,000; GRCH37) by ≥ 80% and (b) a willingness and ability to travel to Atlanta, Georgia. Exclusion criteria were (a) any del 3q29 with less than 80% overlap with the canonical region and (b) nonfluency in English. Subjects selected for the 3q29 study underwent deep phenotyping according to an established protocol [19, 39] and phenotypes were recorded accordingly (Additional file 2: Table S4). Whole blood, drawn from 46 subjects at the study visit, was used for downstream OGM analysis. Optical genome maps were generated using single molecule files (bnx) [40] for 16 probands with the del3q29S and parental genomes (when available) who were of European ancestry (Additional file 1: Figure S1). For ten probands, blood samples from both biological parents were analyzed, and deletions were confirmed to be de novo using aCGH. For four probands, DNA was not available from either of the parents; for two probands, DNA from only one parent was available. Two additional probands with the dup3q29S were also included in the present study; the same protocol was followed for OGM analysis. All subjects had genotyping arrays completed for parent-of-origin analysis. Finally, one trio, Family 15, was used for PacBio HiFi sequencing to refine the breakpoints in the proband.

### The 3q29 Project Family 15 PacBio HiFi sequencing

Family 15 trio was used to produce ≥ 30X PacBio HiFi sequencing [40] for orthogonal support and detection of breakpoint junctions at the base pair level. High-molecular-weight DNA was extracted from the frozen pelleted cells (obtained from induced pluripotent stem cell lines which were reprogrammed with the non-integrating Sendai virus) using the Gentra Puregene kit (Qiagen), following the manufacturer’s instructions. Purified genomic DNA (gDNA) was quantitatively and qualitatively assessed using a Qubit fluorometer (Thermo Fisher) and a FEMTO Pulse (Agilent), respectively. Samples exhibiting a mode size above 50 kbp were considered good candidates for HiFi/CCS sequencing. gDNA was sheared to target 15–18-kbp fragments using gTUBEs (Covaris) with centrifugation settings at 5000 rpm for 2 min, until the sample passed through the tube. The sheared material was subjected to SMRTbell® library preparation using the Template Prep Kit v2 (PacBio) according to the manufacturer’s recommendation. Libraries were subjected to size selection on Pippin HT (Sage Science) to remove templates smaller than 10 kbp. All libraries were sequenced on a Sequel II System (PacBio) using chemistry v2 sequencing kits. Sequencing parameters included a 2-h pre-extension and 30-h movie times. HiFi/CCS analysis was performed using SMRT® Link v10 with default parameters. For each sample, three SMRT® cells were used to obtain ≥ 30X sequencing coverage.

### Bionano Genomics optical mapping high-molecular-weight DNA extraction

Ultra-high-molecular-weight DNA was extracted from cell lines of 1000GP and CIAPM samples (*n* = 114), and from whole blood samples from the 3q29 Project (*n* = 46) according to the Bionano Prep SP Fresh Cells DNA Isolation protocol (revision C, Document #30,257), using a Bionano SP Blood & Cell DNA Isolation Kit (catalog #80,030). In short, 1.5 million cells were centrifuged and resuspended in a solution containing detergents, proteinase K, and RNase A. DNA was bound to a silica disk, washed, eluted, and homogenized via 1-h end-over-end rotation at 15 rpm, followed by an overnight rest at room temperature. Isolated DNA was fluorescently tagged at motif CTTAAG by the enzyme DLE-1 and counter-stained using a Bionano Prep™ DNA Labeling Kit – Direct Label and Stain (catalog #8005) according to the Bionano Prep DLS Protocol (revision F, Document #30,206). A total of 750 ng of purified gDNA was labeled by incubating with DL-Green dye and DLE-1 Enzyme in DLE-1 Buffer for 2 h at 37 °C, followed by heat inactivation of the enzyme for 20 min at 70 °C. The labeled DNA was treated with Proteinase K at 50 °C for 1 h, and excess DL-Green dye was removed by membrane adsorption. The DNA was stored at 4 °C overnight to facilitate DNA homogenization and then quantified using a Qubit dsDNA HS Assay Kit (Molecular Probes/Life Technologies). Labeled DNA was stained with an intercalating dye and left to stand at room temperature for at least 2 h before loading onto a Bionano Saphyr Chip®. The DNA was loaded onto the Bionano Genomics Saphyr® system for linearization and visualization. Data collection was performed using Saphyr® 2^nd^ generation instruments (Part #60,325) and Instrument Control Software (ICS) version 4.9.19316.1. The DNA backbone length and locations of fluorescent labels along each molecule were detected using the Saphyr® system software.

### De novo assembly of optical genome maps

Single-molecule optical genome maps of 1000GP, CIAPM, and the 3q29 Project samples were assembled de novo and aligned to the GRCh38 reference assembly using the Bionano Solve v3.5 (https://bionanogenomics.com/support/software-downloads/) assembly pipeline, with default settings as described previously [34, 41]. In short, a pairwise comparison of DNA molecules (min 250 kbp) was generated to produce the initial consensus genome maps. During an extension step, molecules were aligned to genome maps, and maps were extended based on the molecules aligning past the map ends. Overlapping genome maps were then merged. Extension and merge steps were repeated five times before a final refinement of the genome maps. Clusters of molecules aligned to genome maps with unaligned ends > 30 kbp in the extension step were re-assembled to identify all alleles. To identify alternate alleles with smaller size differences from the assembled allele, clusters of molecules aligned to genome maps with internal alignment gaps of size < 50 kbp were detected, and the genome maps were converted into two haplotype maps. The final genome maps were aligned to the reference genome, GRCh38.

### Single-molecule analysis and haplotype construction pipeline for optical genome maps

Structural variations (SVs) and haplotypes at the 3q29 locus were analyzed, and single molecule support was collected [42, 43] from all samples using the Optical Maps to Genotype Structural Variation (OMGenSV, https://github.com/yuliamostovoy/OMGenSV) package as described previously [13, 14]. To identify 3q29 haplotypes in samples from 114 unaffected individuals (UCSF dataset), the assembled contigs were visualized using the “anchor” mode in OMView from the OMTools package (https://github.com/TF-Chan-Lab/OMTools) [44]. The Bionano Access™ software was used for HGSVC (*n* = 3), HPRC (*n* = 44), and the 3q29 Project (*n* = 46) samples. Haplotypes were manually identified from these visualizations, and corresponding consensus map (cmap) files were constructed for each haplotype to evaluate single molecule support [42, 43]. If the contig in the 3q29 locus was not contiguous, at least 500 kbp of the unique flanking region where applicable was included. When the 3q29 haplotypes were substantially long (> 500 kbp), molecules were subdivided into groups that were anchored in the proximal or distal unique regions of 3q29 SDA and SDB. For each haplotype, the corresponding cmaps were compiled into a single file and used as a reference input file for the OMGenSV pipeline, along with local molecules from each sample. A set of “critical regions” was also included (GRCh38; SDA: 195,578,485–195,817,578 Mbp; SDB: 195,804,017–196,073,500 Mbp; SDC: 197,557,633–197,743,251 Mbp) to define the areas on each cmap that molecules need to span to support the presence of that haplotype in the sample. The 3q29 Project sample cohort haplotypes were identified by using unaffected individuals’ 3q29 haplotype maps as a reference, and molecule support was confirmed by following the steps described above. Images of molecule support were obtained by using OMTools and Bionano Access™ (Availability of data and materials; Additional file 3) [42, 43]. To confirm that the structure of 3q29 haplotypes was not biased by GRCh38 alignment, we selected six unaffected individuals (BC00701, BC03702, HG01358, HG01573, HG02055, and HG03863) and two 3q29 probands (from Family 1 and Family 2) for an additional analysis. These samples were then aligned to the Telomere-to-telomere (T2T) reference assembly (chm13v2.fa) using refAligner (Bionano Solve™ v3.5.1).

Next, we evaluated whether there was an association between haplotypes and populations. After calculating the expected value for each haplotype, Fisher’s exact test was used to test the significance of the 3q29 haplotype frequencies in each population using RStudio (version 1.4.1717). Finally, Cohen-Friendly association plots were generated in RStudio (see Additional file 1: Supplementary Methods).

### Breakpoint mapping and trio analysis of the 3q29 Project samples using optical maps

For each of the 18 probands, de novo assembled contigs were aligned to the GRCh38 assembly using the refAligner tool (Bionano Solve™ v3.5.1) (Additional file 1: Supplementary Methods), and the 3q29 locus was manually inspected to identify deletion and duplication breakpoints. Once identified, the underlying single molecules were examined to verify that deletion and duplication breakpoints were well supported by single molecules (*n* ≥ 3 molecules) (Additional file 3). Then, the sequence identity of the approximate breakpoints and the corresponding sequence in GRCh38 were determined by the *blastn* tool (BLASTN 2.9.0 +) [35, 36], and only “the best alignment” was included in the final output file. Next, we categorized breakpoints into five distinct classes based on the size of the deletion or duplication as well as the breakpoints in SDB and SDC. Next, the haplotypes of the parents were identified as described above, and the parent transmitting the rearranged chromosome (i.e., the chromosome with the deletion or duplication) was identified by a visual pairwise comparison of the haplotypes using Bionano Access™.

### PacBio HiFi de novo assembly and breakpoint detection in Family 15

Family 15 trio samples were assembled de novo by hifiasm [45] (0.16.1-r375, https://github.com/chhylp123/hifiasm) using raw reads. Resulting assemblies were used for variant calling by svim-asm [46] (v1.0.2, https://github.com/eldariont/svim-asm). To determine the contigs that aligned to chr3:195,000,000–197,700,000 (i.e., the region of interest), de novo assemblies were aligned to GRCh38 using minimap2 [47]. Contigs aligned to the region of interest were used for multiple sequence alignment by using mafft [48] (v7.310, https://mafft.cbrc.jp/alignment/software/source.html) to detect the parent-of-origin chromosome as well as the parental origin of the intact chromosome. Jalview [49] (v2_11_2, http://www.jalview.org/getdown/release/) was used for the visualization of alignment results. The deletion breakpoints in the proband were identified by using variant calls from svim-asm. Secondary structure prediction for the breakpoint region was performed by using RNAfold (http://rna.tbi.univie.ac.at/cgi-bin/RNAWebSuite/RNAfold.cgi). Finally, 3q29 segments in the Family 15 trio were detected using GRCh38 3q29 segments as the reference by blastn (BLASTN 2.9.0 +) [35, 36]. The resulting alignment files were visualized using genoPlotR (v0.8.11) [50].

### Comparison and validation using orthogonal data

Publicly available OGM and PA data for the 44 unaffected individuals from the HPRC were analyzed to (1) determine the 3q29 haplotypes by using OGM data and, subsequently, (2) validate the haplotype structures using PA data. First, HPRC PA fasta files were converted to in silico maps using fa2cmap_multi_color.pl (Bionano Solve™ v3.5.1). Resulting in silico maps were aligned to the GRCh38 using the refAligner tool (Bionano Solve™ v3.5.1) (see Additional file 1: Supplementary Methods). Haplotypes in each sample were detected using the 3q29 segments as a guide, as described above. Finally, for each sample, a visual pairwise comparison of OGM and PA 3q29 haplotypes was performed using Bionano Access™.

## Results

### De novo assembled optical genome maps of unaffected individuals reveal unique haplotypes in the 3q29 locus

**Fig. 1:**
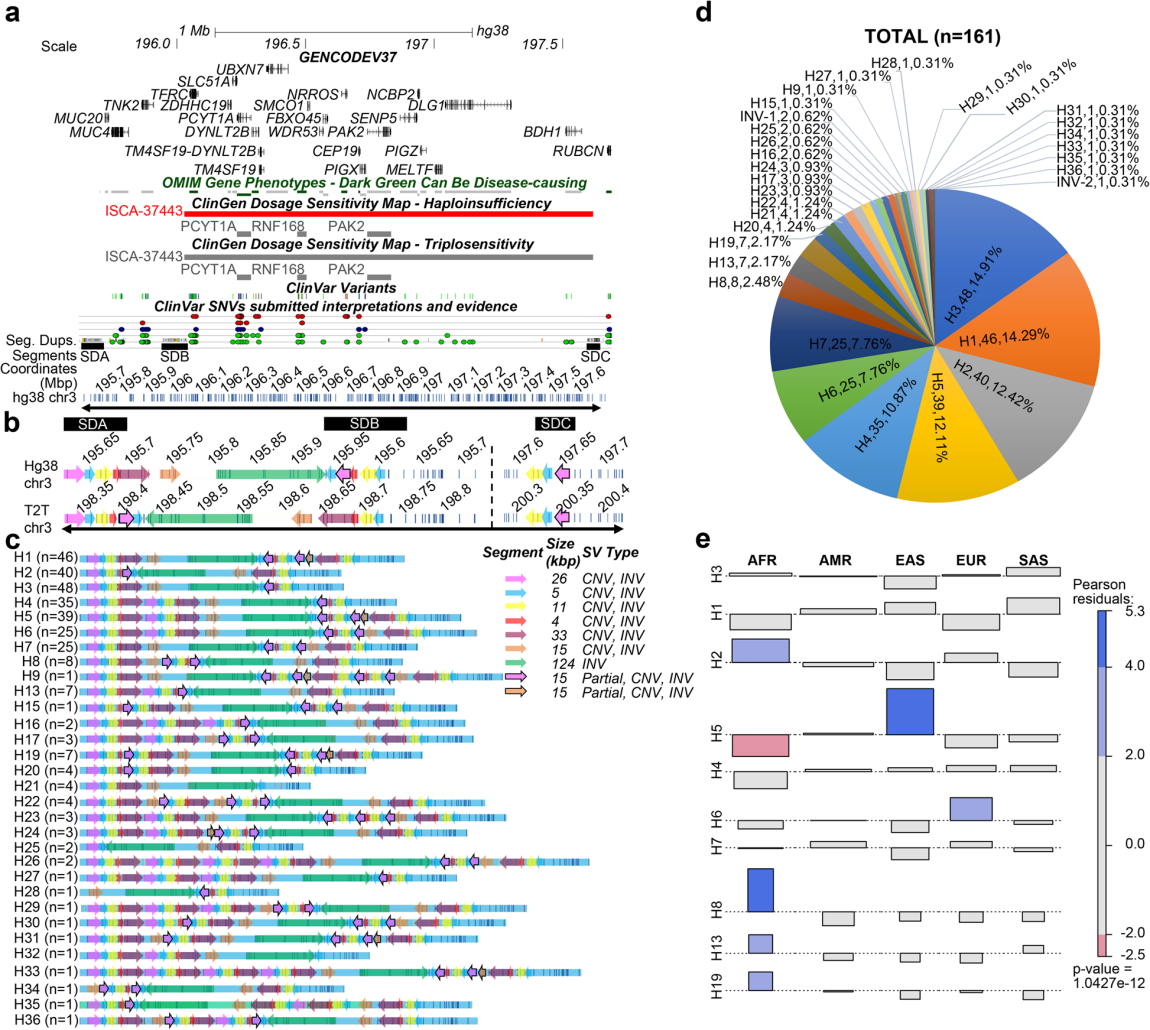
The segment structure of GRCh38 3q29 region and haplotypes identified in this study. **a** 3q29 locus with SDA, SDB, SDC, OMIM Gene Phenotypes, ClinGen Dosage Sensitivity Map—Haploinsufficiency, ClinGen Dosage Sensitivity Map—Triplosensitivity, ClinVar Variants, ClinVar SNVs, and Segmental Duplications are represented as Tracks. The 3q29 GRCh38 in silico map is represented in the last track. ClinVar Track: red dots, pathogenic; dark blue dots, variants of uncertain significance; green dots, benign variants. **b** 3q29 segments of GRCh38 and T2T with SDA, SDB, and SDC represented as black boxes on top overlaid on the in silico maps (white background with vertical blue lines). Dashed line: the unique region between SDB and SDC. Black arrows in panels **a** and **b**—the region included in our analyses. **c** The structure and prevalence of 13 known haplotypes identified among our samples (H1-H9, H13, H15-H17) and 18 novel haplotypes, which were ordered by frequency (H19-H36). Each colored arrow represents 3q29 segments. Partial, partial copy of 32q9 segments; CNV, copy number polymorphism; INV, inversion. **d** Prevalence of the 3q29 haplotypes represented in unaffected individuals. **e** Cohen-Friendly association plot depicting the relationship between haplotypes and populations. If the observed count is greater than expected, the rectangle rises above the baseline and is colored in blue. If the observed count is less than expected, the rectangle falls below the baseline and is colored in red

The 3q29 locus contains three SD blocks within a 2-Mbp region. They are referred to as SDA (73 kbp), SDB (64 kbp), and SDC (41 kbp) identified by OGM labeling pattern and sequence identity, as described previously [34] (Fig. 1a, b; Additional file 2: Table S1). These SD blocks overlap with 21 protein-coding genes, noncoding RNAs, and other genomic elements. In this study, we used de novo assembled optical genome maps for individuals (*n* = 161) from the 1000GP and CIAPM cohorts, which had an average effective coverage of 102.4X and an average molecule N50 of 301.96 kbp (Additional file 2: Table S5).

We detected a total of 33 different haplotypes for the 3q29 locus among these 161 unaffected individuals (Fig. 1c). Two haplotypes carried large inversions between SDA and SDC (INV-1 and INV-2), and the remaining 31 haplotypes were based on different types and numbers of SVs observed within the SDA and SDB blocks across individuals. No variation was observed within the SDC block across samples. Some of the SVs within SDA and SDB, ranging from 1 bp to 550 kbp, were previously identified and reported on the Database of Genomic Variants (DGV) (Additional file 2: Table S6) and often overlap protein-coding genes (e.g., *MUC4* and *MUC20*), pseudogenes (e.g., *SDHAP1* and *SDHAP2*), and lincRNAs (e.g., lncRNA MUC20-OT1) (Fig. 1a, c). The haplotype size of the 3q29 locus ranged from 287 kbp (H28, HG03863-SAS) to 859 kbp (H26, BC02901-AMR, and BC03702-EUR) (Fig. 1c; Additional file 1: Figure S2; Additional file 2: Table S7). The smallest haplotype, H28, is missing the proximal 440 kbp and the distal 132.5 kbp of the largest haplotype, H26, and lacks five copies of the following segments: 26 kbp, 5 kbp, 11 kbp, and 33 kbp. Among our 161 unaffected samples, we detected 18 novel haplotypes, labeled H19-H36. The most common three haplotypes among all unaffected individuals were H3 (14.91%), H1 (14.29%), and H2 (12.42%) (Fig. 1d). The H3 haplotype represents the GRCh38 reference assembly haplotype, and the H2 haplotype represents the recent human genome assembly from the Telomere-to-Telomere (T2T) Consortium [51] (Fig. 1c). We also detected 14 singleton haplotypes, i.e., haplotypes observed only once among the 322 chromosomes examined from the 161 unaffected individuals (Fig. 1d).

We found significant differences in haplotype frequency between populations (Fig. 1d, e, p*-value* = *0.000001*; *Fisher’s exact test*; Additional file 1: Figure S3). One of the most common haplotypes, H3, was observed at ≥ 7% frequency in all five super populations and 14% in four populations (Fig. 1d). In the AFR population, three haplotypes, H2 (22%), H3 (15%), and H18 (11%), accounted for 48% of the haplotype pool, with H2 being significantly enriched compared to other haplotypes and all other populations (*p-value* = *0.009*, *Fisher’s exact test*) (Fig. 1e; Additional file 1: Figure S3a). Interestingly, the H8 haplotype, which contains the second-largest inversion observed in the 3q29 region (289 kbp), was observed only in the AFR population (*p-value* = *5.73972e − 06*, *Fisher’s exact test*). In the AMR population, H1 (18%), H3 (14%), and H5 (13%) represented the three most common haplotypes (Fig. 1e; Additional file 1: Figure S3b). In the EAS population, the H5 (41%) haplotype was enriched compared to the other haplotypes and all other populations (Fig. 1e; *p*-value = 7.731026e − 08, Fisher’s exact test; Additional file 1: Figure S3c). In the EUR population, the H6 (19%) haplotype was enriched compared to other haplotypes and all other populations (Fig. 1e; *p*-value = 0.0002, Fisher’s exact test; Additional file 1: Figure S3d). Finally, in the SAS population, the H1 (26%) haplotype was enriched compared to the other haplotypes and all other populations (Fig. 1e; *p*-value = 0.002, based on Fisher’s exact test; Additional file 1: Figure S3e).

To validate the structure composition of haplotypes identified by OGM data, we identified the number and order of 3q29 segments in each HPRC haplotype-resolved PA using *blastn* and GRCh38 3q29 segments. We also converted haplotype-resolved PA fasta files to in silico maps (see the “Methods” section) and overlaid PA (HPRC dataset, *n* = 44) onto the OGM-based haplotypes. We detected 20 haplotypes, 11 of which were previously identified [34] and 9 of which were novel, that were consistent between the OGM and PA datasets (Additional file 1: Figures S4-7; Additional file 2: Table S7). One discrepancy was detected between the OGM and PA haplotype structures for one chromosome in sample HG01928. The PA suggested that the haplotypes were H3/H5, and the OGM data suggested that the haplotypes were H4/H5. Single DNA molecules (*n* ≥ 10) from the OGM data, spanning at least 90% of the 3q29 locus, were anchored to the proximal and distal unique regions and confirmed the presence of the H4 haplotype in HG01928 (Additional file 2: Figure S8). Haplotype H4 had additional copies of the first five segments (magenta, blue, yellow, red, and maroon) compared to H3, and single molecules supported the presence of these copies in HG01928.

Furthermore, we used OGM data from three trios (HG00512, HG00513, and HG00514; HG00731, HG00732, and HG00733; NA19238, NA19239, and NA19240) of the 1000GP sample cohort to confirm that the 3q29 haplotype structures we observed from OGM data were not artifacts and not due to clonal variation in the cell lines. Findings from these analyses clearly showed that the haplotype structures we observed in the trio children, HG00514, HG00733, and NA19240, were indeed identical to the haplotype structures that were inherited from maternal and paternal chromosomes (Additional file 1: Figure S9). In addition, to investigate whether 3q29 haplotype structures are biased by the GRCh38 reference assembly during the alignment step, we aligned six unaffected individuals (BC00701, BC03702, HG01358, HG01573, HG02055, and HG03863) and two probands with del 3q29 to the T2T assembly. Results demonstrated that the structures of 3q29 haplotypes were consistent between the two alignments and were not biased by any reference assemblies (Additional file 1: Figure S10).

### Inversions among the 3q29 haplotypes

Previous research has provided evidence that certain inversions can have significant implications for human health. [52]. In fact, some studies have linked de novo deletions and duplications to inversions as a risk factor [53, 54]. The importance of inversions in the human genome is emphasized by the forces of natural selection and random drift that influence their impact, geographic distributions, and frequency in populations based on their phenotypic effects [52, 55, 56].

**Fig. 2:**
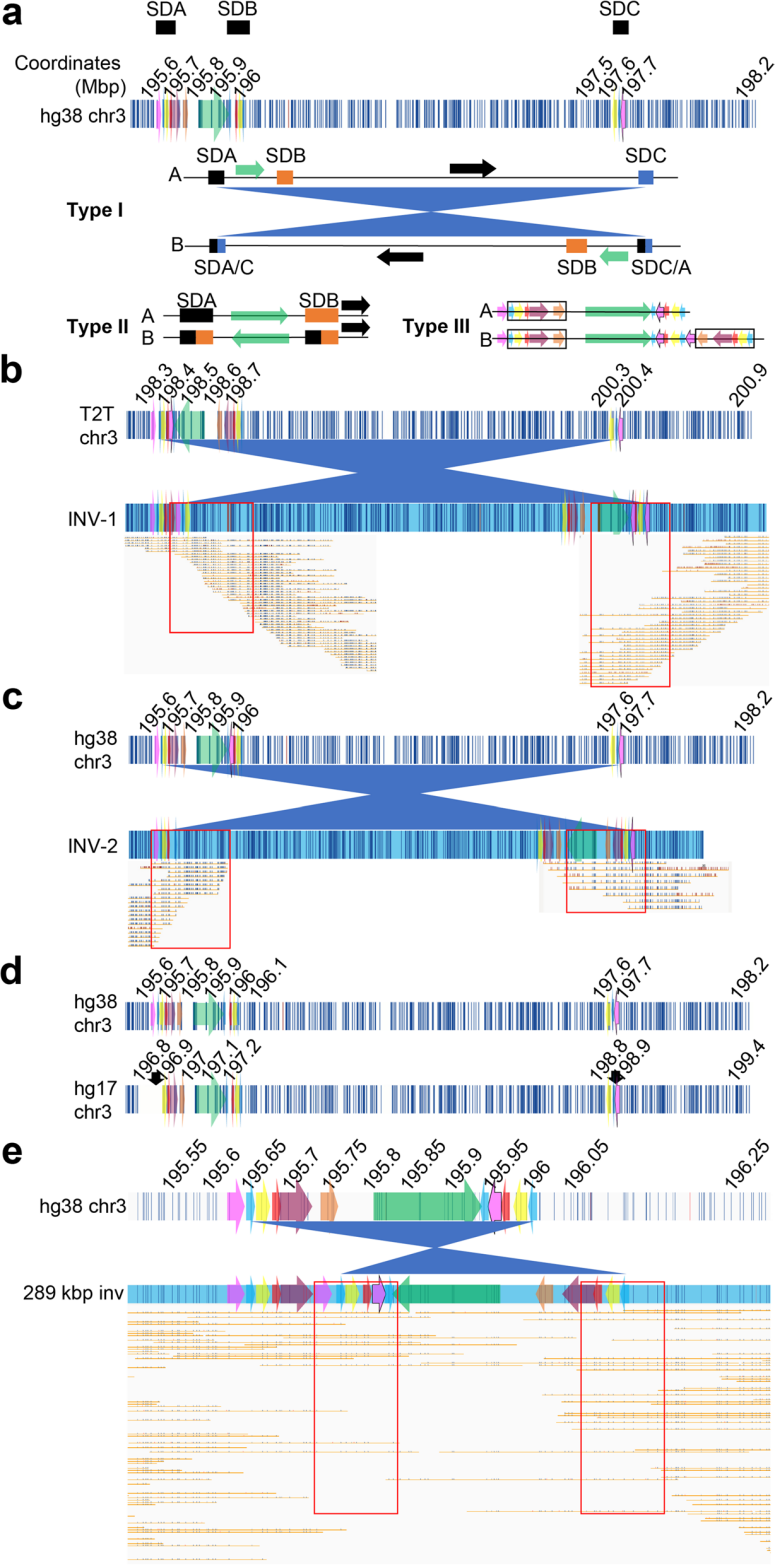
Inversions identified from the 1000GP and CIAPM samples. **a** The structure of in silico hg38 3q29 locus represented at the top with white background and dark vertical lines. The SD blocks were presented above, 3q29 segments (colored arrows) were overlaid on. Three types of inversions, Type I, Type II, and Type III, detected from this study were presented below the in silico map. Type I represents inversions > 2 mbp in size, Type II represents inversions ~ 289 kbp in size, and Type III represents inverted duplications of five 3q29 segments (black rectangles). Black, orange and blue boxes: 3q29 SD blocks; green arrow: 3q29 segment; black arrow: unique region between SDB and SDC. Strand A at the top, displays reference structures and strand B below represents inverted structures. **b** Upper rectangle with white background and vertical lines represents chm13/T2T in silico map of the 3q29 locus. The rectangle at the bottom represents the structure of INV-1 (~ 2.03 Mbp). Red rectangle: highlighting the molecules supporting the inversion breakpoints. **c** Upper rectangle with white background and vertical lines represents hg38 in silico map of the 3q29 locus. The rectangle at the bottom represents the structure of INV-2 (~ 2.13 Mbp). Red rectangle: highlighting the molecules supporting the inversion breakpoints. **d** hg38 and NCBI35/hg17 reference assemblies represented with white backgrounds and vertical blue lines. Black arrows in NCBI35/hg17 represent inversion breakpoints identified by Antonacci and colleagues [57]. Colored arrows in each panel represent 3q29 segments. **e** hg38 human genome reference assembly presented at the top and the 289-kbp inversion, including the 3q29 segments, represented at the bottom. The red rectangle highlights the molecules supporting the inversion breakpoints

We sought to determine if any 3q29 haplotypes with inversions are unique to any populations and whether any inverted haplotypes were detected in 3q29 probands or their parents. We detected three different types of inversions in 23% of 3q29 haplotypes (Fig. 2a; Additional file 1: Figure S11a). Type I inversions are > 2 Mbp in size, occur between SDA and SDC, and were identified among three unaffected individuals (Fig. 2b, c). The INV-1 haplotype carrying the 2.03 Mbp inversion was found in HG03470-AFR (Fig. 2b; Additional file 1: Figure S11b), and the INV-2 haplotype was found in two individuals, GM19984-AFR and BC04902-AMR (Fig. 2c; Additional file 1: Figure S11b, c). Both inversions are similar to the 2-Mbp inversion previously described by Antonacci and colleagues (Fig. 2d) [57]. The breakpoints of this 2-Mbp inversion were localized to a 15.5-kbp region (chr3:196,868,578–196,884,133 and chr3:198,832,975–198,848,521; hg17/UCSC 2004). In this study, we found that breakpoints for inversions > 2 Mbp were clustered to paralogous 5-kbp copies within SDA and SDC blocks (Fig. 2d). These paralogous, inverted copies share 98% sequence identity, suggesting NAHR as the mechanism causing these inversions.

The second type of inversion is 289 kbp in size and occurs between SDA and SDB (Fig. 2e). This is the most prevalent inversion and was observed at a frequency > 10% in all five super populations on 12 distinct haplotypes: H2, H8, H13, H16, H17, H22, H24, H25, H29, H34, H35, and H36. This previously reported inversion was observed on seven novel and five previously identified haplotypes among 69 unaffected individuals (76 chromosomes; Additional file 2: Table S7) [58, 59]. The third type of inversion is an inverted duplication of any size within the 3q29 segments (Fig. 2a; Additional file 1: Figure S11a).

The prevalence of type I and II inversion haplotypes in the five superpopulations ranges from 4% in the EAS populations to 49% among the AFR populations (Additional file 1: Figure S11b). Interestingly, although seven distinct haplotypes (H2, H13, H16, H24, H25, H35, and INV-2) with inversions were identified in the AMR population, the H2 haplotype was overwhelmingly the dominant haplotype (Additional file 1: Figure S11c). Strikingly, a majority of haplotypes identified in the EAS population were non-inverted (96%) and only two inversion haplotypes, H2 and H22, were observed, comprising only 4% of the total number of haplotypes in this population (Additional file 1: Figure S11d). Similar to the observation in the AMR population, H2 was the dominant inverted haplotype out of 24 percent of inverted haplotypes identified in the EUR population, with 18% occurrence (Additional file 1: Figure S11e). Finally, only three inverted haplotypes, H2, H13, and H17, comprising 13% of all the haplotypes, were identified in the SAS population (Additional file 1: Figure S11f).

**Fig. 3:**
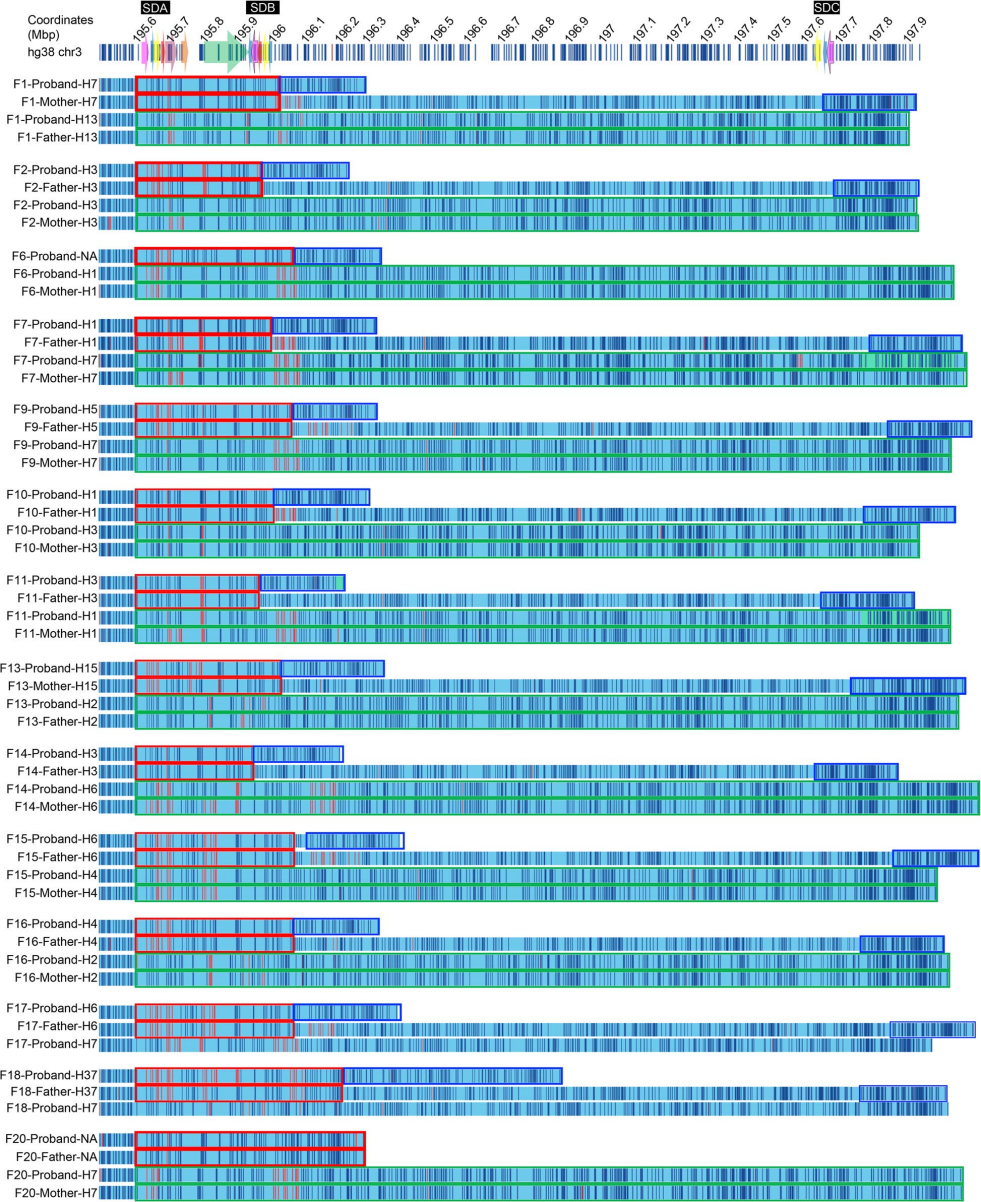
Haplotype structures observed in 3q29 probands and their parents. Red—proximal region in deleted chromosome and proximal region in parent of origin chromosome. Blue—distal region in deleted chromosome and distal region in parent of origin chromosome. Green—intact chromosome in proband and chromosome of the parent which transmitted the intact chromosome. F, family

We also evaluated inversions in the 3q29 Project cohort as well. Strikingly, none of the haplotypes identified in parent-of-origin chromosomes (H1, H3, H4, H5, H6, and H7) carry INV-1, INV-2, or the most common 289-kbp inversion (Fig. 3; Additional file 2: Table S8). Haplotypes identified in paternal or maternal inherited chromosomes (inherited as an intact chromosome) were H2 (2/10), H3 (2/10), H7 (2/10), H1 (1/10), H4 (1/10), H6 (1/10), and H13 (1/10). Haplotype structures of parent-of-origin chromosomes from families where only one parent’s sample was available were H6, H37, and H2 (Family 6, 17, and 18). We could not detect the exact structure of the 3q29 haplotype in four probands. Two probands (Family 3 and 4) did not have family samples available, one proband (Family 6) had only one available parental sample, who was not the parent-of-origin, and one proband’s (Family 20) deletion was inherited from the father. Nevertheless, haplotype structure in the proximal region of deletions suggested that none of these samples have INV-1, INV-2, or the common 289-kbp inversion haplotype.

### Identification of potential risk factor haplotypes in 3q29 deletion/duplication probands

In this study, we analyzed probands with the del3q29S (*n* = 16) and the dup3q29S (*n* = 2) from the 3q29 Project using OGM technology with an average effective coverage of assembly 102.8X and an average molecule N50 of 299.14 kbp (Additional file 2: Table S9).

The recurrent ~ 1.6-Mbp deletion is known to localize to SDB and SDC blocks [60]. On the other hand, the unique genomic sequence residing between SDA and SDB is typically left intact. All 16 patients had the recurrent del 3q29 de novo, except one, who inherited the deletion from his father. This father appeared to be affected by schizophrenia, a generalized disorder, and ADHD. In another proband, we identified a smaller ~ 1.2-Mbp deletion lying completely within the ~ 1.6-Mbp del 3q29 region (Fig. 3, Family 18) and containing 19 of the 21 protein-coding genes. The proximal breakpoint falls within the *TFRC* gene, deleting the promoter and the first four exons. On the distal side, the *BDH1* gene is intact. Interestingly, despite its smaller deletion, there is no discernible difference in phenotypic severity in this proband compared to the probands with ~ 1.6 Mbp of deletion (Additional file 2: Table S4).

Furthermore, we studied two probands (proband only: Family 12 and 19) with the dup3q29S. One proband (Family 19—proband only) had a recurrent ~ 1.6-Mbp duplication, which appears to be a reciprocal duplication of the recurrent ~ 1.6-Mbp deletion causing the del3q29S. The 3q29 haplotypes identified in this proband were H5 and H7 (Additional file 1: Figure S12; Additional file 2: Table S8). The second proband had a nonrecurrent duplication, and the 3q29 haplotypes in this proband were H1 and H2 (Family 12—proband only; Additional file 1: Figure S13; Additional file 2: Table S8).

In order to investigate whether certain haplotypes are predisposed to the del3q29S or dup3q29S, we identified 3q29 haplotypes in probands (Fig. 3b, Additional file 2: Table S8) and available parents. Where possible, we determined the parent-of-origin chromosome and which chromosome was inherited in an intact state. We identified a novel haplotype, H37, in one father (Family 18), who was the parent of origin of the deletion observed in the proband (Additional file 1: Figure S14). Although we did not observe a significant enrichment of del 3q29s or duplications with any particular haplotype, analysis results in trios showed that H3 (3/10) and H1 (2/10) were the two most prominent haplotypes identified in parent-of-origin chromosomes (Fig. 3; Additional file 2: Table S8). Furthermore, we evaluated each of the 3q29 haplotypes that were identified in probands and disease phenotypes. Results showed that all six probands with H2 (*n* = 4) and H13 (*n* = 2) haplotypes, except for one proband (Family 3), had graphomotor weakness, and all six probands with H2 (*n* = 4) and H13 (*n* = 2) haplotypes, except for one proband (Family 18), had ADHD. On the other hand, none of the probands with H2 and H13 haplotypes, except for one proband (Family 18), had an intellectual disability.

Our results demonstrated that H1 (25%), H3 (17%), and H6 (17%) haplotypes (Fig. 3; Additional file 2: Table S8) were observed more often than other haplotypes in the proband’s deleted chromosomes. Similarly, H2 (28%) and H7 (22%) haplotypes were observed at a higher frequency compared to other haplotypes detected in the proband’s intact chromosomes. Although every participant in the 3q29 Project was of EUR descent, surprisingly, the H4 haplotype, which is the most enriched haplotype in unaffected individuals of EUR descent, was not the most frequent haplotype detected in proband deletion chromosomes. In addition, we did not observe any difference in the frequency of del 3q29s or dup 3q29s among the 3q29 Project population under study. Together, these findings suggest that certain 3q29 haplotypes, some of which are specific to ancestral populations, may be practical in identifying risk factors for del3q29S.

### Long-read sequencing reveals breakpoint junction in Family 15

**Fig. 4:**
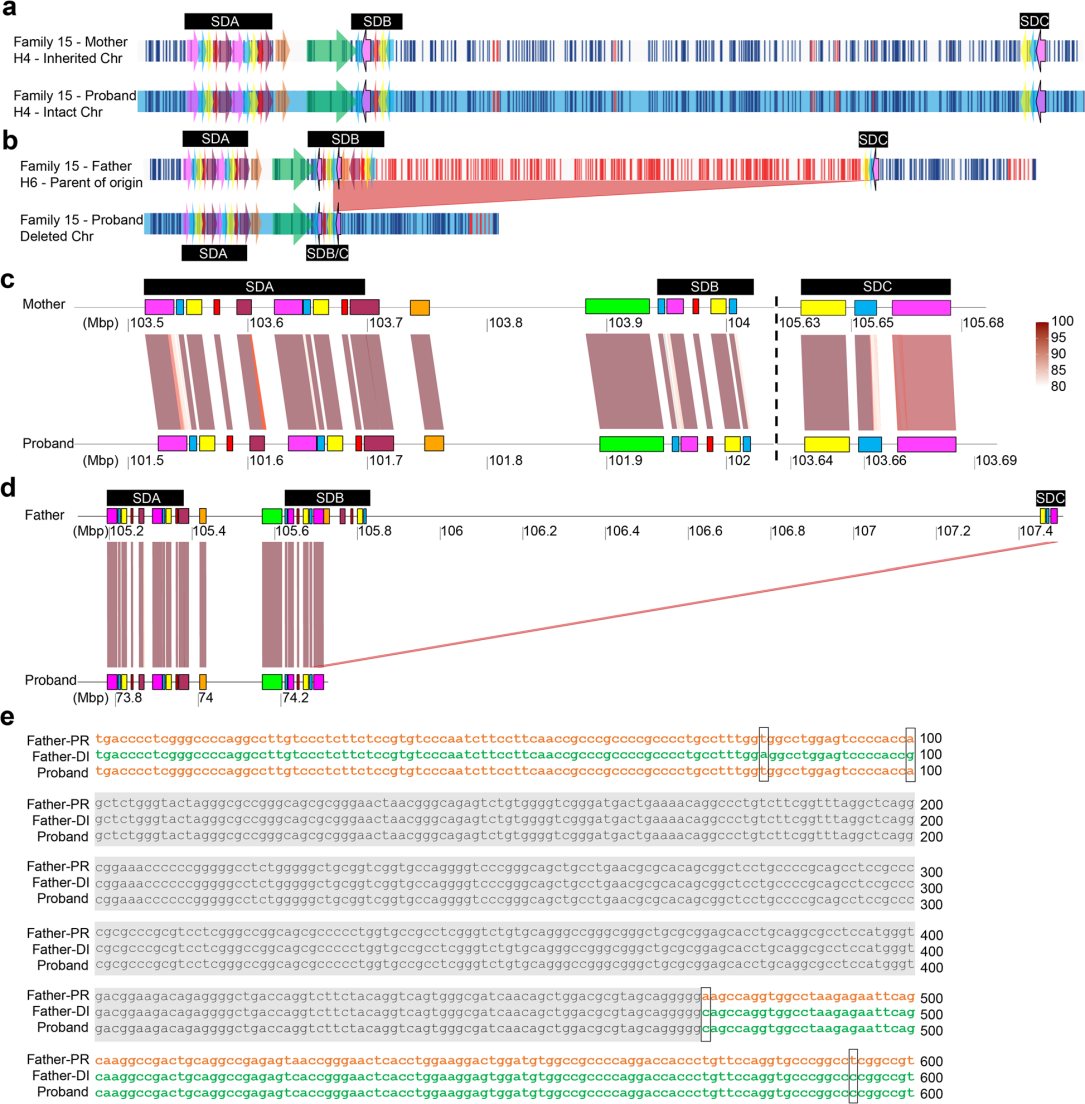
Breakpoint analysis in Family 15 proband. **a** OGM data representing the intact chromosome that was inherited from mother. **b** OGM data representing the parent of origin, Father, and deleted chromosome. Red shaded area represents the deleted region. **c**, **d** Homology plots generated using genoPlotR based on PacBio HiFi sequence data depicting the structure of the 3q29 locus: **c** in the inherited chromosome of the mother and chromosome of the proband. **d** in the parent of origin chromosome of the father and the deleted chromosome of the proband (deletion breakpoint junction: 74,235,310 to 74,299,419). **e** Sequence alignment of the proximal (orange font) and distal region (green font) of the parent-of-origin chromosome of the father to the deletion breakpoint sequence of the proband, likely generated by a the putative cross-over event happened during NAHR. PR, proximal; DI, distal; gray shaded area—breakpoint junction; black boxes—sequence variants identified in parent-of-origin and deleted chromosomes

While it is known that the del 3q29 and dup 3q29 breakpoints mostly occur within two SD blocks, SDB and SDC, the breakpoint locations have not yet been precisely mapped. In order to resolve breakpoints at the nucleotide level, we generated 30X PacBio HiFi LRS data for the Family 15 trio and constructed de novo assemblies for each individual. Firstly, we confirmed that the de novo assembled haplotype-resolved 3q29 haplotypes were consistent with the haplotypes from the OGM data. The haplotypes identified in this family were proband: H6 (paternal deleted chromosome) and H4 (maternal intact chromosome); mother: H6 (not transmitted) and H4 (transmitted); and father: H6 (transmitted) and H2 (not transmitted) (Fig. 4a–d). The structure, order, copy number, and orientation of all segments in each 3q29 haplotype in this family were 100% consistent between the two technologies. Next, we confirmed the parent-of-origin and non-inherited chromosomes and narrowed down the deletion breakpoints by comparing the de novo assembled haplotypes at the sequence level. We performed multiple sequence alignment of the sequence (250 kbp) spanning the deletion breakpoints in the proband and sequences from parent-of-origin (father), H6, proximal (286 kbp), and distal (185 kbp) regions that appear to be localizing to the deletion breakpoints. Then, we evaluated alignment results by using the genotypes of paralogous sequence variants (e.g., SNVs, indels) within SDs and refined the deletion breakpoints to a 374-bp interval (Fig. 4e; Additional file 2: Table S10). Highly similar sequences within the 15-kbp segment suggested that this deletion was mediated by NAHR (Fig. 4d). This was further supported by the presence of a predicted secondary structure showing a hairpin formation (Additional file 1: Figure S15) which was potentially enhanced by the presence of simple repeat elements in the region ((GCGT)n (1–121) and (GCGCCC)n (1–54)). To the best of our knowledge, this is the most precisely determined deletion breakpoint for any del 3q29 case currently available.

### Defining five breakpoint classes for the 3q29 deletion and duplication syndromes

**Fig. 5:**
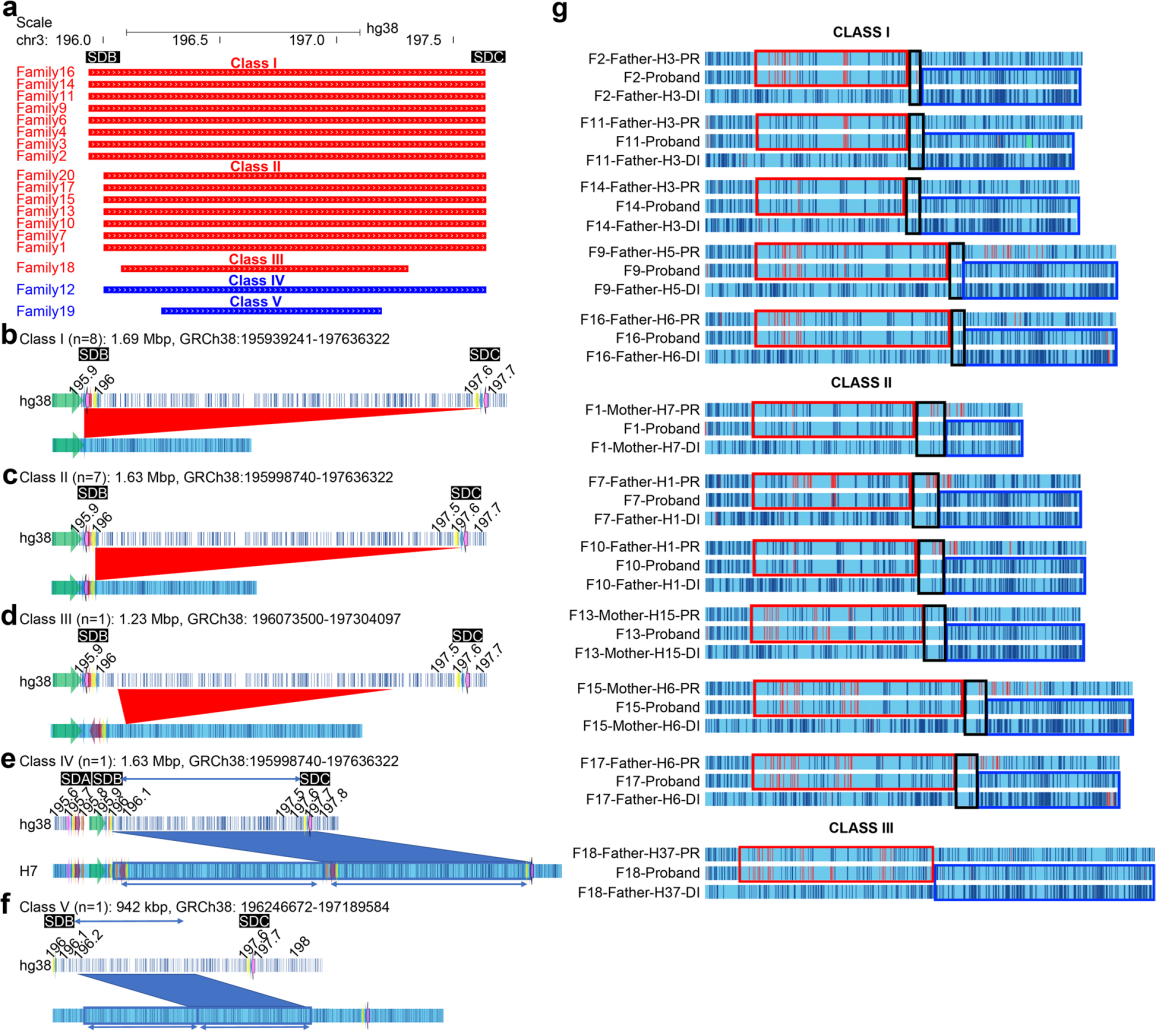
Deletion and duplication breakpoint classes identified in our study. **a** Size and breakpoint location of deletion and duplication patients from the 3q29 Project. Red—3q29 deletion, blue—3q29 duplication. **b–f** The breakpoint structures of deletions in probands that were categorized as: **b** Class I. **c** Class II. **d** Class III, and the breakpoint structure of duplications in probands were categorized as: **e** Class IV. **f** Class V. Colored arrows represent the 3q29 segments. Red triangles—depicting the deleted region. Blue rectangles—depicting the duplicated region. **g** In silico maps of trios showing the NAHR region in each deletion breakpoint class. Red rectangles—proximal deletion region, blue rectangles—distal deletion region, green—intact chromosome in probands and the chromosomes inherited to the proband by the transmitting parent. PR—proximal region with respect to deletion, DI—distal region with respect to deletion

Our in-depth investigation of probands with the del3q29S (*n* = 16) and the dup3q29S (*n* = 2) allowed us to identify five distinct breakpoint classes, based on the size of the deletion or duplication and the breakpoint position, either in SDB or SDC (Additional file 2: Table S11). A majority of the breakpoints (3/5) localized to paralogous segments with ≥ 98% sequence identity (Fig. 5a). The deletion breakpoints among the probands with the del 3q29 can be divided into three deletion classes: Class I (8 probands), Class II (7 probands), and Class III (1 proband) (Fig. 5a–d). In Class I deletions (1.69 Mbp, 195.94–197.64 Mbp; GRCh38), the proximal breakpoint localized to the first blue 5-kbp segment in SDB and the distal 5-kbp paralogous copy in SDC. Although OGM data indicated that the deletion breakpoints localized to the 5-kbp segments, the possibility of breakpoint localization to the 15-kbp segments (magenta, partial copies of the SDA-26 kbp segment) in SDB and SDC cannot be excluded. The OGM resolution within this 15-kbp segment in SDB and SDC was insufficient as only one label resides within the 26-kbp segment (magenta) in SDA and no labels within the 15-kbp segment. Therefore, sequence-level resolution is required to determine whether paralogous copies of 5 kbp or 15 kbp cause NAHR in deletions classified as Class I. In Class II deletions (1.63 Mbp, 195.99–197.64 Mbp; GRCh38), the proximal breakpoint localized to the second 5-kbp segment in SDB and the distal breakpoint localized to the paralogous 5-kbp segment in SDC. However, LRS data revealed that although long homology around the deletion breakpoint includes 5-kbp (blue) segments, the breakpoint junction was within the partial 15-kbp segment (Fig. 5c). Results from LRS data analysis of Family 15 (see above) revealed that the crossover event happened at the 374-bp breakpoint junction, which suggests probands with the Class II deletion breakpoints might have a similar breakpoint junction. However, resolution at the sequence level is still required to confirm it. In Class III deletions (1.23 Mbp, 196.01–197.3 Mbp; GRCh38), the proximal and distal breakpoints did not overlap with any SDs (Fig. 5d).

Additionally, we analyzed two probands with duplications and categorized breakpoints into two classes: Class IV (1.6 Mbp; GRCh38; chr3:196–197.6 Mbp) and Class V (942 kbp; GRCh38; chr3:196.25–197.2 Mbp) (Fig. 5e, f). The Class IV duplication appears to be a reciprocal duplication of the recurrent 1.6-Mbp deletion (Class II) (Fig. 5c). The proximal breakpoint in Class IV localized to the second 5-kbp segment (blue) in SDB and the distal breakpoint to the paralogous 5-kbp segment in SDC (Fig. 5e), therefore likely caused by NAHR. In Class V, the proximal and distal breakpoints localized to a unique region between SDB and SDC (Fig. 5f), suggesting a different mechanism [60].

We investigated breakpoint classes to evaluate whether there was a correlation between the phenotypes of del 3q29 and dup 3q29 probands. We did not detect any significant associations between the proband’s 3q29 haplotypes and phenotypes (Additional file 2: Table S12). For instance, we observed ADHD in at least half of the probands in both Class I (4 out of 8) and Class II (6 out of 7), indicating no significant associations with either one of the breakpoint classes.

In summary, breakpoints identified in three classes (I, II, and IV) are localized to the paralogous copies of the same segment and occurred as a result of NAHR. Sequence analysis of the paralogous segments in SDB and SDC in the GRCh38 showed high sequence identity to each other (≥ 98%) suggesting that they can facilitate NAHR. On the other hand, the proximal and distal breakpoints in nonrecurrent deletion in Class III and duplication in Class V were not localized to SDs, and the sequence identity between breakpoints was not high (< 80%), indicating that these rearrangements occurred as a result of a different mechanism, such as non-homologous end joining or replication-based mechanism [60, 61].

## Discussion

Chromosome-scale haplotype-resolved de novo genome assemblies enable characterization of the fine structure of SDs and of the rearrangements caused by SD segments, ultimately allowing the construction of haplotype maps of these complex regions. Using these maps to detect haplotypes in samples provides an unparalleled opportunity to investigate whether certain haplotypes serve as risk or protective factors that impact individuals’ predisposition to genomic disorders.

In this study, we constructed de novo assemblies of the 3q29 locus to identify distinct haplotypes using OGM technology. Ebert and colleagues [34] previously reported 18 haplotypes in 30 unaffected individuals. We have now identified an additional 19 novel haplotypes in unaffected individuals, one of whom is the unaffected father of a proband with the del 3q29, and bringing the total number of known haplotypes for this region to 37. Furthermore, six haplotypes are either population-specific or have low frequency in other populations. For instance, the H8 haplotype was enriched in 11% of the AFR population, the H5 haplotype was enriched in 41% of the EAS population, the H6 haplotype was enriched in 19% of the EUR population, and the H1 haplotype was enriched in 26% of the SAS population. The 1000GP trio samples (HG00512, HG00513, HG00514, HG00731, HG00732, HG00733, NA19238, NA19239, and NA19240) showed undeniably which haplotypes were inherited from maternal and paternal chromosomes and indicated that the complexity of this locus is not due to clonal variation in the cell lines (Additional file 1: Figure S8).

The 3q29 haplotypes contain inversions and copy number changes of 3q29 segments that overlap with protein-coding genes, pseudogenes, and non-coding RNAs, such as *MUC20* and *MUC4*, lncRNA MUC20-OT1, *SDHAP1*, *SDHAP2*, *SMBD1P*, and miRNAs (Additional file 2: Table S1). A recent study showed that lncRNA *SDHAP1* upregulated the expression of *EIF4G2* by reducing miR-4465 levels in ovarian cancer cells [62], which suggests that the pseudogenes may regulate gene expression through microRNAs [63]. Therefore, additional copies of the pseudogenes may impact patients’ phenotypes by regulating the protein-coding genes in the 3q29 region. However, further studies need to be conducted to investigate the function of the pseudogenes in the etiology of del3q29S and dup3q29S.

We analyzed the 3q29 haplotype structures in probands with del3q29S (*n* = 16) and dup3q29S (*n* = 2) to determine the breakpoint locations more precisely and to identify the molecular mechanisms responsible for the deletions. In 16 of 18 probands, the deletion breakpoints (Class I, Class II and Class IV) overlapped paralogous copies within SDB and SDC, located in the same orientation. High sequence identity (> 98%) between the paralogous segments suggests that an NAHR caused the deletions and duplications in these probands, consistent with previous findings [10]. The Class III and Class V breakpoints did not occur within any SD blocks, indicating that this is a nonrecurrent rearrangement in the region and implying that alternative molecular mechanisms, such as NHEJ, fork stalling and template switching, microhomology-mediated break-induced replication, or retrotransposition, are involved in their formation. [60, 61].

While certain 3q29 haplotypes may be useful in identifying risk factors for del3q29S, our analysis did not reveal any significant associations between haplotypes and breakpoint classes. However, the H3 (3/10) and H1 (2/10) haplotypes were detected more frequently, suggesting that these haplotypes could still be potential contributors to an increased risk for del3q29S and dup3q29S.

Inversions have previously been hypothesized to be a risk factor for some de novo deletions and duplications [53]. It is thought that inversions can interfere with synapsis during meiosis, potentially causing DNA loops that are susceptible to misalignment and/or breakage [2]. For example, at the Williams-Beuren syndrome locus, there is an enrichment of inversions in the parents, where the de novo deletion arises [64, 65]. However, in 2003, a group studying the 22q11.2 deletion syndrome showed that none of the parents of 18 probands with the 22q11.2 deletion syndrome carried an inversion at the 22q11.2 locus [66]. For the 3q29 locus, two types of inversions, 289 kbp and 2 Mbp, have previously been reported [57, 58]. The breakpoints of these inversions were localized to paralogous copies of 3q29 segments between SDA and SDB, and SDA and SDC. Among the 22 parents analyzed in the current study, six were found to carry the 289 kbp inversion, and only three (H2: *n* = 2, H13: *n* = 1) were inherited by the proband in an intact state. Strikingly, none of the parent-of-origin chromosomes had carried INV-1 or INV-2, or the common 289 kbp inversion. Thus, our data do not support the notion that any of these inversions predisposes to deletion or duplication at the 3q29 locus. On the contrary, inversions in this locus may protect individuals from del3q29S or dup3q29S. In addition, results from our study indicate that ordering a whole genome sequencing panel to screen for inversions may not be the most efficient step to take as part of the genetic counseling procedure.

One limitation of our study is the lack of resolution of deletion and duplication breakpoints at the sequence level in samples with OGM data, except for Family 15. Nevertheless, with OGM, we refined the deletion and duplication breakpoints to specific paralogous copies within SD blocks. Family 15 LRS data showed that breakpoints can be refined at the nucleotide level and proved the added value of LRS in elucidating breakpoints. Further studies could be pursued to get a better understanding of the underlying structures and sequence content surrounding breakpoints and breakpoint junctions. Furthermore, larger cohorts of samples are needed to determine whether the underlying haplotype structures predispose individuals to del3q29S or dup3q29S or whether specific 3q29 haplotypes in particular populations are associated with these genomic disorders.

## Conclusions

In summary, we have identified a total of 19 novel haplotypes in the 3q29 region of the human genome, among unaffected individuals (18/19) and the 3q29 Project samples (1/19). Some of these haplotypes have significant enrichment in certain populations. Further, we have defined five breakpoint classes and find no evidence for inversions predisposing individuals to del3q29S and dup3q29S. Defining the haplotypes in this complex chromosome region allows clinicians to define breakpoints more accurately and conduct future genotype–phenotype association studies.

## Supplementary Information

**Additional file 1: Supplementary Methods and Figures. Figure S1.** Description of samples included in this study. **Figure S2.** The smallest and the largest haplotypes. **Figure S3.** The AQpopulation distribution of 3q29 haplotypes. **Figure S4.** HPRC haplotype comparison – Part 1. **Figure S5.** HPRC haplotype comparison – Part 2. **Figure S6.** HPRC haplotype comparison – Part 3. **Figure S7.** HPRC haplotype comparison – Part 4. **Figure S8.** HG01928 3q29 haplotype comparison. **Figure S9.** 1000GP trio representing the 3q29 haplotypes. **Figure S10.** GRCh38 and T2T alignment comparisons. **Figure S11.** Inversion Frequencies. **Figure S12.** Molecule support for duplication in Family 19 – proband only sample. **Figure S13.** Molecule support for duplication in Family 12 – proband only sample. **Figure S14.** Novel 3q29 haplotype. Figure S15. Predicted secondary structure of the breakpoint.

**Additional file 2: Table S1.** The 3q29 Segments. **Table S2. **Segment Sequence Identity. **Table S3.** Samples and Data Collection Summary. **Table S4.** The 3q29 Project Sample Cohort Phenotypes. **Table S5.** 1000 Genomes Project and California Initiative to Advance Precision Medicine Samples Optical Genome Mapping Statistics. **Table S6.** Database of Genomic Variants genomic variants Overlapping 3q29 segments. **Table S7.** 1000 Genomes Project and California Initiative to Advance Precision Medicine Samples Haplotypes. **Table S8.** The 3q29 Project Samples Haplotypes. **Table S9.** The 3q29 Project Samples Optical Genome Mapping Statistics. **Table S10.** Variants Identified in Family 15 Proband and Father. **Table S11.** The 3q29 Project Proband Breakpoint Classes. **Table S12.** The 3q29 Project Proband Breakpoint Classes and Phenotype Comparison.

**Additional file 3.** Molecule support for the 3q29 Project samples.

## Data Availability

The 3q29 Project optical mapping and PacBio HiFi datasets are available under the following accession number PRJEB60229 in the NCBI BioProject database (https://www.ebi.ac.uk/ena/browser/view/PRJEB60229) [43], HGSVC dataset is available under the following accession number PRJEB41077 in the NCBI BioProject database (https://www.ebi.ac.uk/ena/browser/view/PRJEB41077), UCSF dataset (including 52 1000GP samples and 65 CIAPM samples) were available under the following accession number PRJNA588278 in the NCBI BioProject database (https://www.ebi.ac.uk/ena/browser/view/PRJNA588278), HPRC dataset is available on cloud services (https://s3-us-west-2.amazonaws.com/human-pangenomics/index.html?prefix=working/), HGSVC samples molecule support is available from figshare (https://doi.org/10.6084/m9.figshare.16899313.v1) [57], and UCSF samples molecule support is available from figshare (https://doi.org/10.6084/m9.figshare.16886500.v3) [41].

## Abbreviations

SV: Structural variants
CNV: Copy number variants
SNV: Single nucleotide variants
NAHR: Non-allelic homologous recombination
SD: Segmental duplications
PA: Phased assembly
1000GP: 1000 Genomes Project
HGSVC: Human Genome Structural Variation Consortium
HPRC: Human Pangenome Reference Consortium
CIAPM: California Initiative to Advance Precision Medicine
LRS: Long-read sequencing
BG: Bionano Genomics
OGM: Optical genome mapping
