# Supplementary material for: High level of complexity and global diversity of the 3q29 locus revealed by optical mapping and long-read sequencing
Source: Genome Med. 2023 May 10;15:35. doi: 10.1186/s13073-023-01184-5 (PMC10170684; doi:10.1186/s13073-023-01184-5)
Supplement: Supplementary file 1 — Additional file 1: Supplementary Methods and Figures. Figure S1. Description of samples included in this study. Figure S2. The smallest and the largest haplotypes. Figure S3. The AQpopulation distribution of 3q29 haplotypes. Figure S4. HPRC haplotype comparison – Part 1. Figure S5. HPRC haplotype comparison – Part 2. Figure S6. HPRC haplotype comparison – Part 3. Figure S7. HPRC haplotype comparison – Part 4. Figure S8. HG01928 3q29 haplotype comparison. Figure S9. 1000GP trio representing the 3q29 haplotypes. Figure S10. GRCh38 and T2T alignment comparisons. Figure S11. Inversion Frequencies. Figure S12. Molecule support for duplication in Family 19 – proband only sample. Figure S13. Molecule support for duplication in Family 12 – proband only sample. Figure S14. Novel 3q29 haplotype. Figure S15. Predicted secondary structure of the breakpoint. [file 13073_2023_1184_MOESM1_ESM.pdf]

Additional file 1

Supplementary Methods and Figures for

“High level of complexity and global diversity of the 3q29 locus revealed by optical mapping and long-read sequencing”

Feyza Yilmaz[1,9], Umamaheswaran Gurusamy[2,9], Trenell J. Mosley[3], Pille Hallast[1], Kwondo Kim[1], Yulia Mostovoy[2], Ryan Purcell[4], Tamim H. Shaikh[5], Michael E. Zwick[6], Pui-Yan Kwok[2,7], Charles Lee[1,*], Jennifer G. Mulle[8,*]

*Corresponding authors: charles.lee@jax.org, jm2618@cabm.rutgers.edu

The file includes:

Supplementary Methods

Supplementary Figures S1 to S15

Supplementary Methods

3q29 segment detection within segmental duplication blocks **3**

3q29 haplotype association analysis **3**

Breakpoint mapping and trio analysis of the 3q29 Project samples using optical maps **4**

PacBio HiFi De novo assembly and breakpoint detection in Family 15 **4**

Comparison and validation using orthogonal data **5**

Supplementary Figures

Figure S1. Description of samples included in this study. **6**

Figure S2. The smallest and the largest haplotypes. **7**

Figure S3. The population distribution of 3q29 haplotypes. **8**

Figure S4. HPRC haplotype comparison – Part 1. **9**

Figure S5. HPRC haplotype comparison – Part 2. **10**

Figure S6. HPRC haplotype comparison – Part 3. **11**

Figure S7. HPRC haplotype comparison – Part 4. **12**

Figure S8. HG01928 3q29 haplotype comparison. **13**

Figure S9. 1000GP trio representing the 3q29 haplotypes. **14**

Figure S10. GRCh38 and T2T alignment comparisons. **15**

Figure S11. Inversion Frequencies. **16**

Figure S12. Molecule support for duplication in Family 19 – proband only sample. **17**

Figure S13. Molecule support for duplication in Family 12 – proband only sample. **18**

Figure S14. Novel 3q29 haplotype. **19**

Figure S15. Predicted secondary structure of the breakpoint. **20**

References **21**

Supplementary Methods

3q29 segment detection within segmental duplication blocks

To identify distinct 3q29 segments within three segmental duplication (SD) blocks and to detect 3q29 segments in long-read sequencing dataset, we used blastn (BLASTN 2.9.0+) (1,2) using the following command:

```
blastn -query $file1 -subject $file2 -subject_besthit -outfmt '6
qstart qend sstart send qseqid sseqid pident length mismatch gaps
evalue bitscore sstrand qcovs qcovhsp qlen slen' -out ${filename}.out
```

3q29 haplotype association analysis

Fisher's exact test was used to test the significance of the 3q29 haplotypes in R Studio (version 1.4.1717). 100,000 replicates were used for the Monte Carlo test. The expected value for each haplotype was calculated by the following command:

```
tab.exp <- round(chisq.test(data)$expected)
```

Then we used expected values for Fisher's exact test:

```
fisher.test(data, simul=T, B =100000)$p.value
```

To evaluate if there is a correlation between haplotypes and populations, we used Cohen-Friendly association plots depicting the relationship between haplotypes and populations. Each cell has a rectangle with height proportional to the difference between observed and expected and width proportional to the square root of expected counts so that the area of the rectangle is proportional to the difference in observed and expected counts. If the observed count is greater than expected, the rectangle rises above the baseline and is colored in orange. Finally, we annotated all haplotypes detected in our study with GENCODE v37 to identify overlapping genomic elements, including protein-coding genes and noncoding RNAs.

Association plots were generated with the following command:

```
tab.sel <- as.matrix(data[freq$Total >5,])

assocplot(tab.sel, shade=TRUE,legend=TRUE
```

Breakpoint mapping and trio analysis of the 3q29 Project samples using optical maps

The sequence identity of approximate breakpoints and the corresponding sequence in GRCh38 was determined by the NCBI *blastn* tool (BLASTN 2.9.0+) (3) with the following command:

```
blastn -query $file1 -subject $file2 -num_alignments 5 -
num_descriptions 5 -out $alignmentoutputfile
```

Only “the best alignment” was included in the final output file.

PacBio HiFi *De novo* assembly and breakpoint detection in Family 15

De novo assembly of Family15 trio samples constructed using raw reads by hifiasm (4) (0.16.1-r375, https://github.com/chhylp123/hifiasm).

```
hifiasm -o $outputdir -t 32 $inputfile
```

Resulting assemblies were used for variant calling by svim-asm (5) (v1.0.2, https://github.com/eldariont/svim-asm).

```
minimap2 -a -x asm5 --cs -r2k -t 16 $ref $hap1 > $aln_hap1.sam

minimap2 -a -x asm5 --cs -r2k -t 16 $ref $hap2 > $aln_hap2.sam

samtools sort -m4G -@4 -o $aln_hap1_sorted.bam $aln_hap1.sam

samtools sort -m4G -@4 -o $aln_hap2_sorted.bam $aln_hap2.sam

samtools index $aln_hap1.sam

samtools index $aln_hap2.sam

svim-asm diploid ${outputdir} $aln_hap1_sorted.bam
$aln_hap2_sorted.bam ${ref}
```

Comparison and validation using orthogonal data

Human Pangenome Reference Consortium phased assembly fasta files were converted to *in silico* maps using a script that comes with Bionano Solve v3.5.1.

```
perl
Solve3.5.1_01142020/HybridScaffold/1.0/scripts/fa2cmap_multi_color.pl
```

Similarly, resulting in silico maps were aligned back to the GRCh38 human genome reference assembly using the “refAligner” alignment tool that comes with Bionano Solve v3.5.1.

```
Solve3.5.1_01142020/RefAligner/1.0/RefAligner -ref ${referenceFile} \
-maxthreads ${threads} \
-i ${inputfile} \
-o ${outputdir}EXP_REFINEFINAL1
```

Supplementary Figures

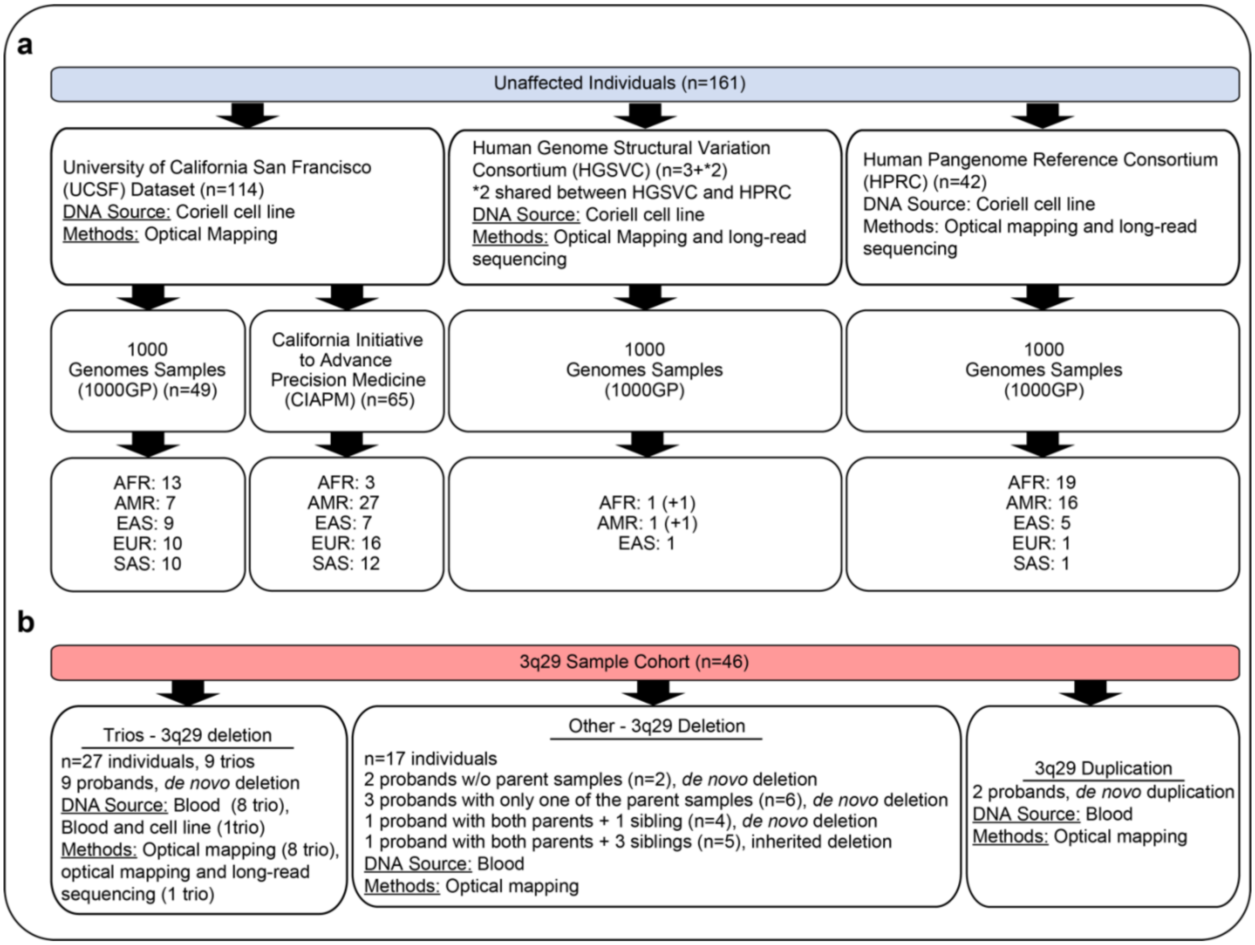

Figure S1 Description of samples included in this study. **a** Unaffected individuals from 1000GP, CIAPM, HGSVC and HPRC. **b** 3q29 sample cohort, including probands with 3q29 deletion or duplication, and their parents.

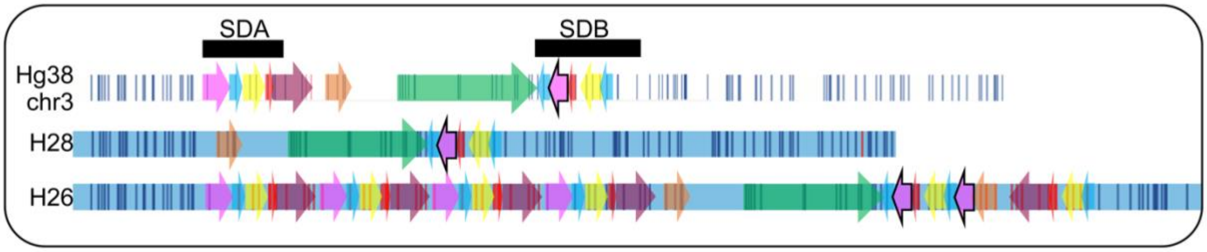

Figure S2 The smallest and the largest haplotypes. Colored arrows represent 3q29 segments, white rectangles with vertical lines represent Hg38/GRCh38 in silico map, and blue rectangles with vertical lines represent H28 and H26-oldH24 haplotypes.

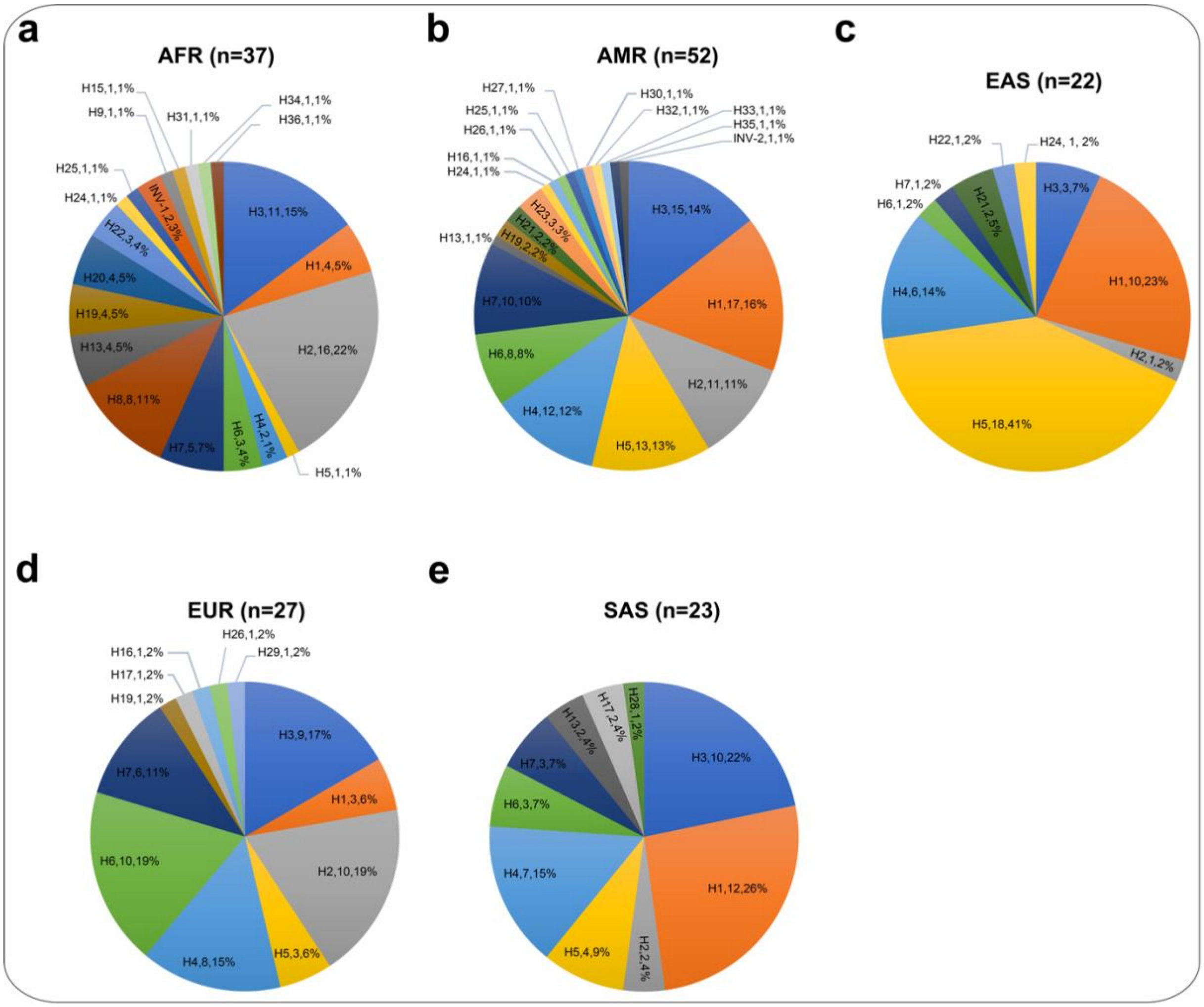

Figure S3 The population distribution of 3q29 haplotypes. Percentages of haplotypes were represented in: **a** AFR population, **b** AMR population, **c** EAS population, **d** EUR population, **e** SAS population. AFR, African; AMR, American; EAS, East Asian; EUR, European; SAS, South Asian. Each color in the pie charts represents a distinct 3q29 haplotype. The labels in the pie charts indicate, in order, haplotype ID, count, and prevalence. H9, H15, H27-H36, and INV-2 are singleton haplotypes. n represents the number of samples.

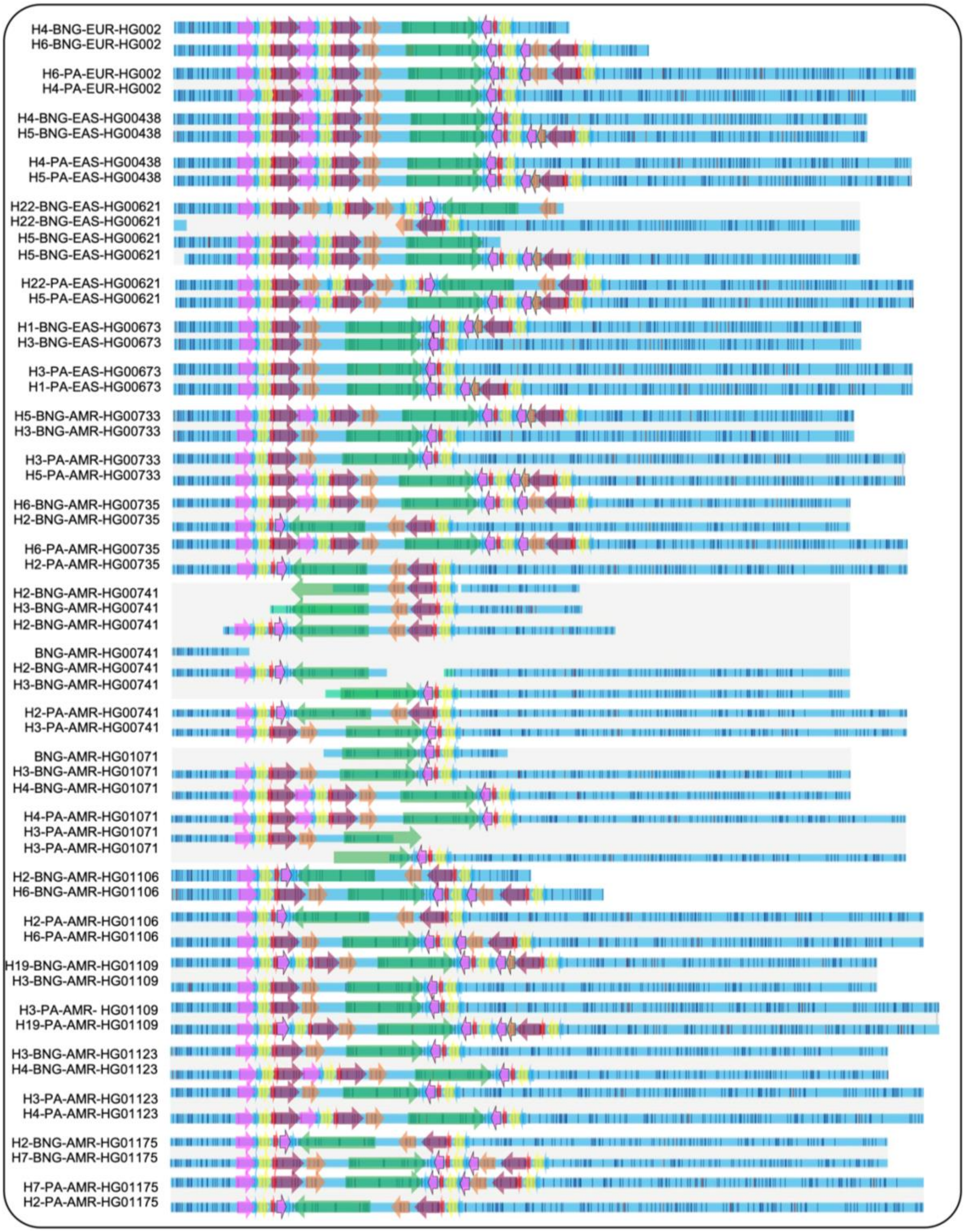

Figure S4 HPRC haplotype comparison – Part 1. EUR: European ancestry, EAS: East Asian ancestry, AMR: American ancestry, BNG: Bionano Genomics optical mapping data, PA: Phased assembly in silico mapping data, colored arrows depicting the 3q29 segments.

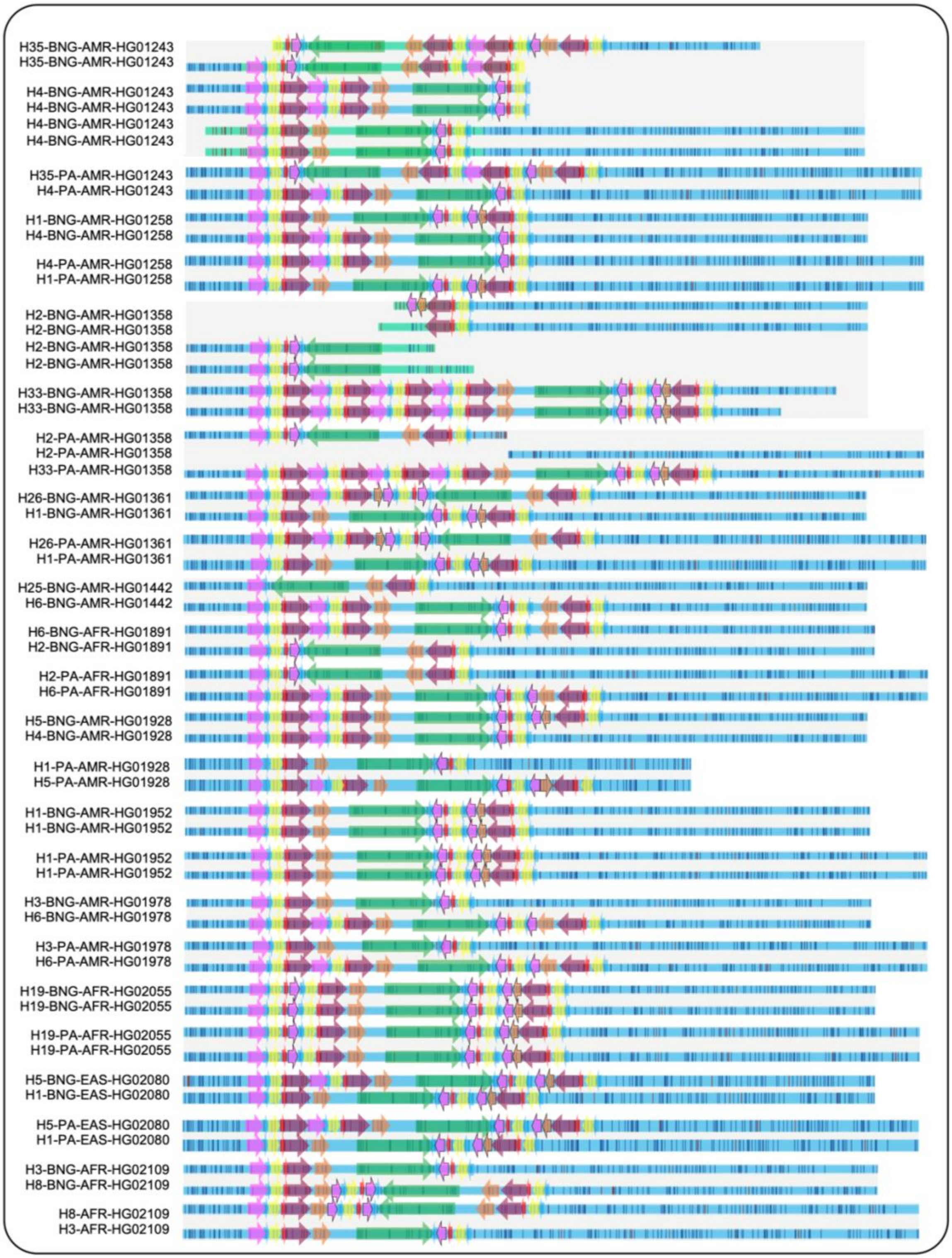

Figure S5 HPRC haplotype comparison – Part 2. AMR: American ancestry, EAS: East Asian ancestry, AFR: African ancestry, BNG: Bionano Genomics optical mapping data, PA: Phased assembly in silico mapping data, colored arrows depicting the 3q29 segments.

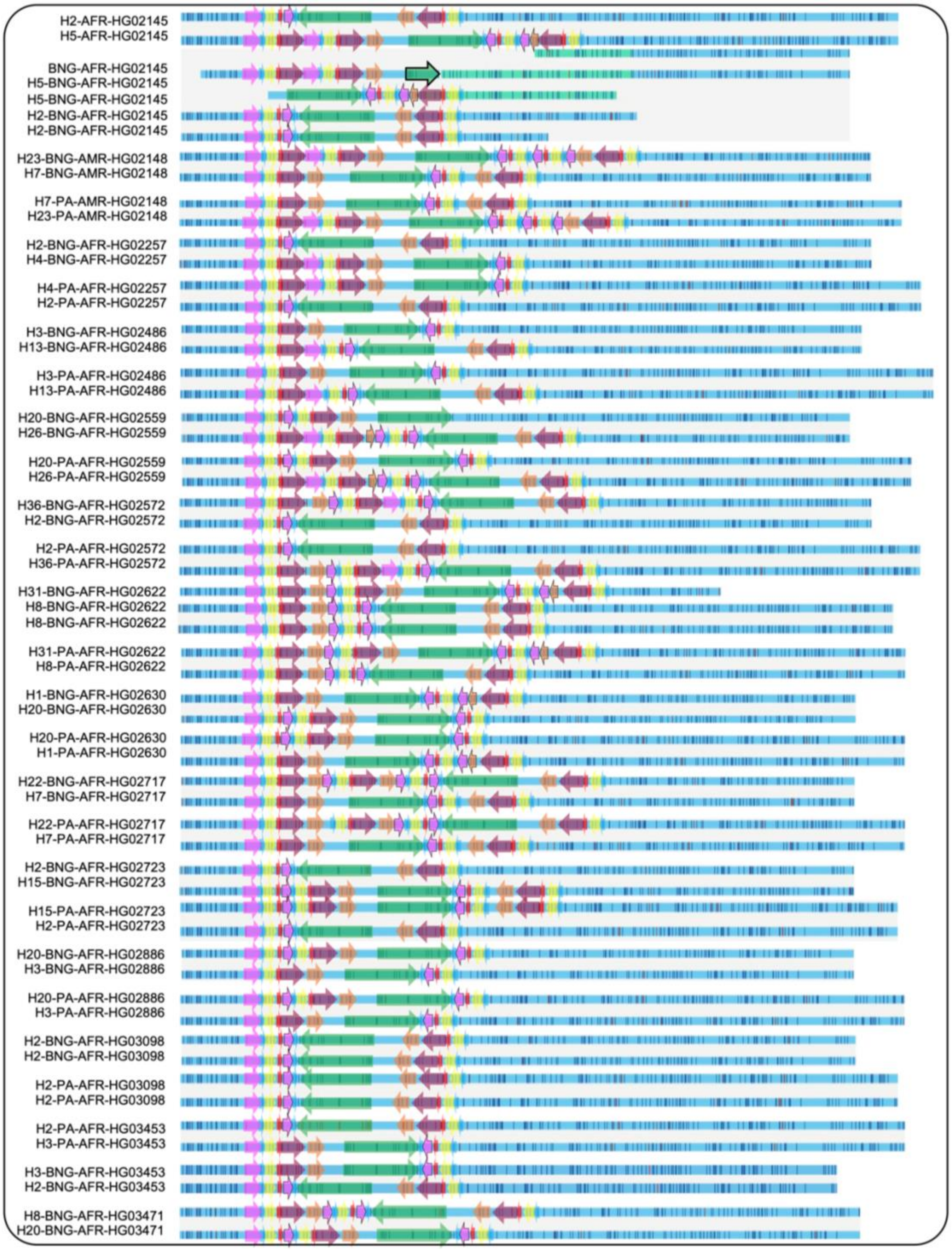

Figure S6 HPRC haplotype comparison – Part 3. AFR: African ancestry, AMR: American ancestry, BNG: Bionano Genomics optical mapping data, PA: Phased assembly in silico mapping data, colored arrows depicting the 3q29 segments.

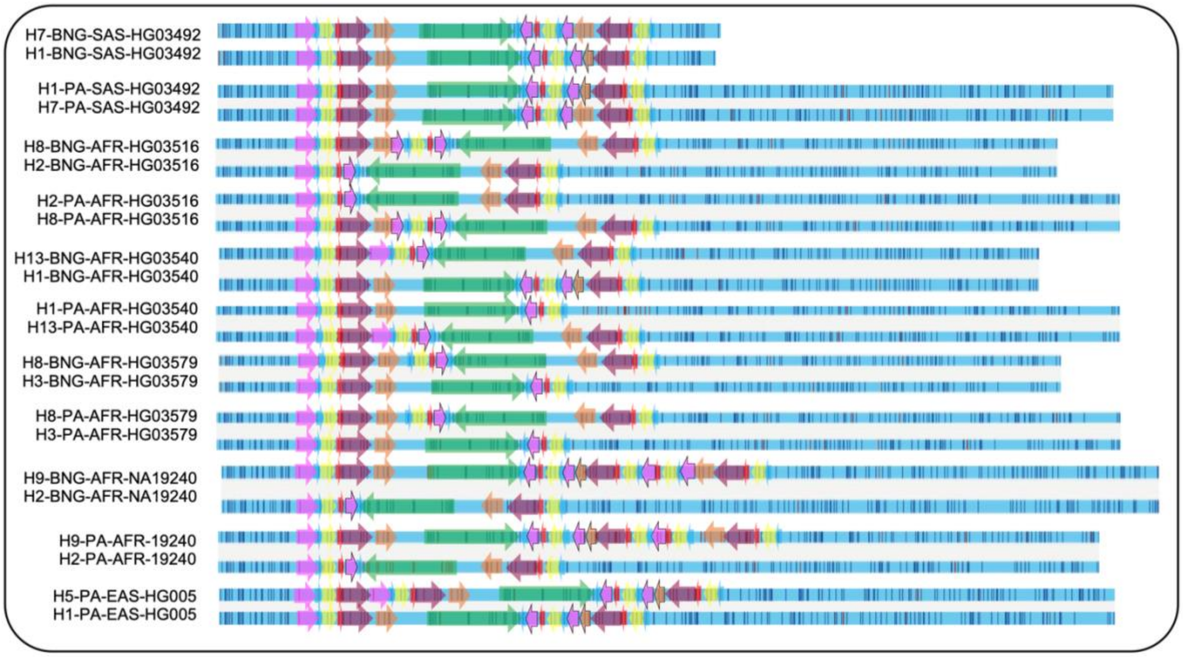

Figure S7 HPRC haplotype comparison – Part 4. AFR: African ancestry, AMR: American ancestry, BNG: Bionano Genomics optical mapping data, PA: Phased assembly in silico mapping data, colored arrows depicting the 3q29 segments.

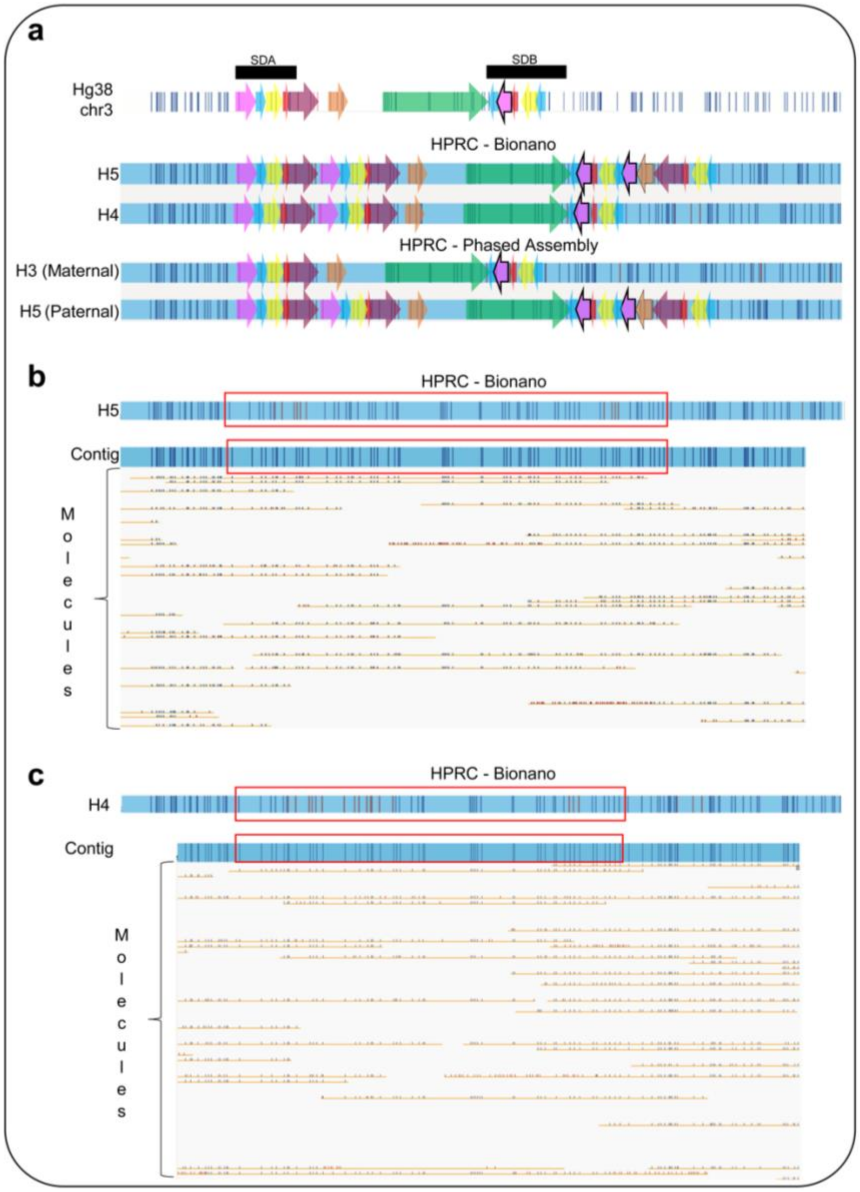

Figure S8 HG01928 3q29 haplotype comparison. **a** hg38 *in silico* map represented at the top, OM haplotypes, H5 and H4 respectively, represented in the middle, and phased assembly maternal and paternal haplotypes are represented at the bottom. **b** OM of the H5 haplotype contigs represented in blue and OM molecules represented as yellow lines showing single molecule support for the H5 haplotype. **c** OM of the H4 haplotype contigs are represented in blue and OM molecules represented as yellow lines showing single molecule support for the H4 haplotype. The red boxes in b and c show the 3q29 region in each haplotype.

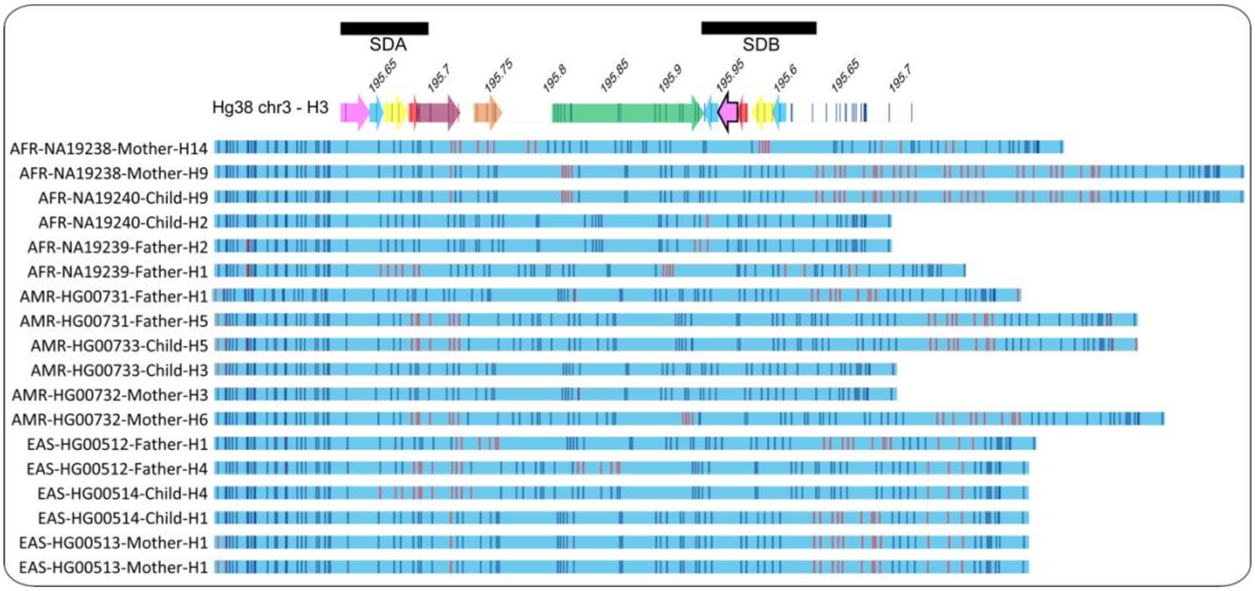

Figures S9 1000GP trio representing the 3q29 haplotypes. Probands' haplotypes were presented in the middle and inherited paternal and maternal chromosomes were placed right below or above the proband chromosomes.

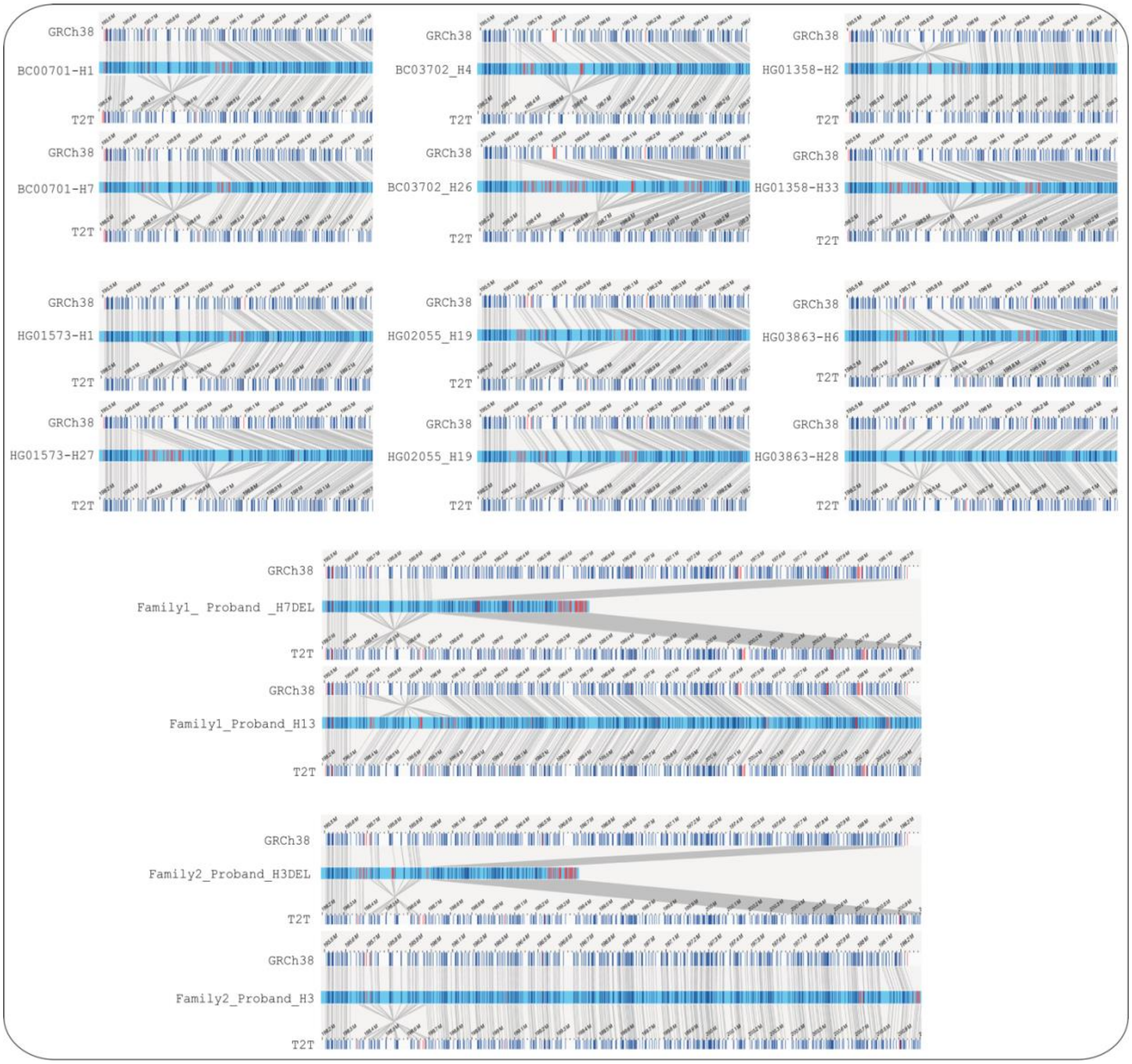

Figure S10 GRCh38 and T2T alignment comparisons. The following samples were included: BC00701, BC03702, HG01358, HG01573, HG02055, HG03863, Family 1 and Family 2. Top panel represents displaying the consistency of 3q29 haplotypes.

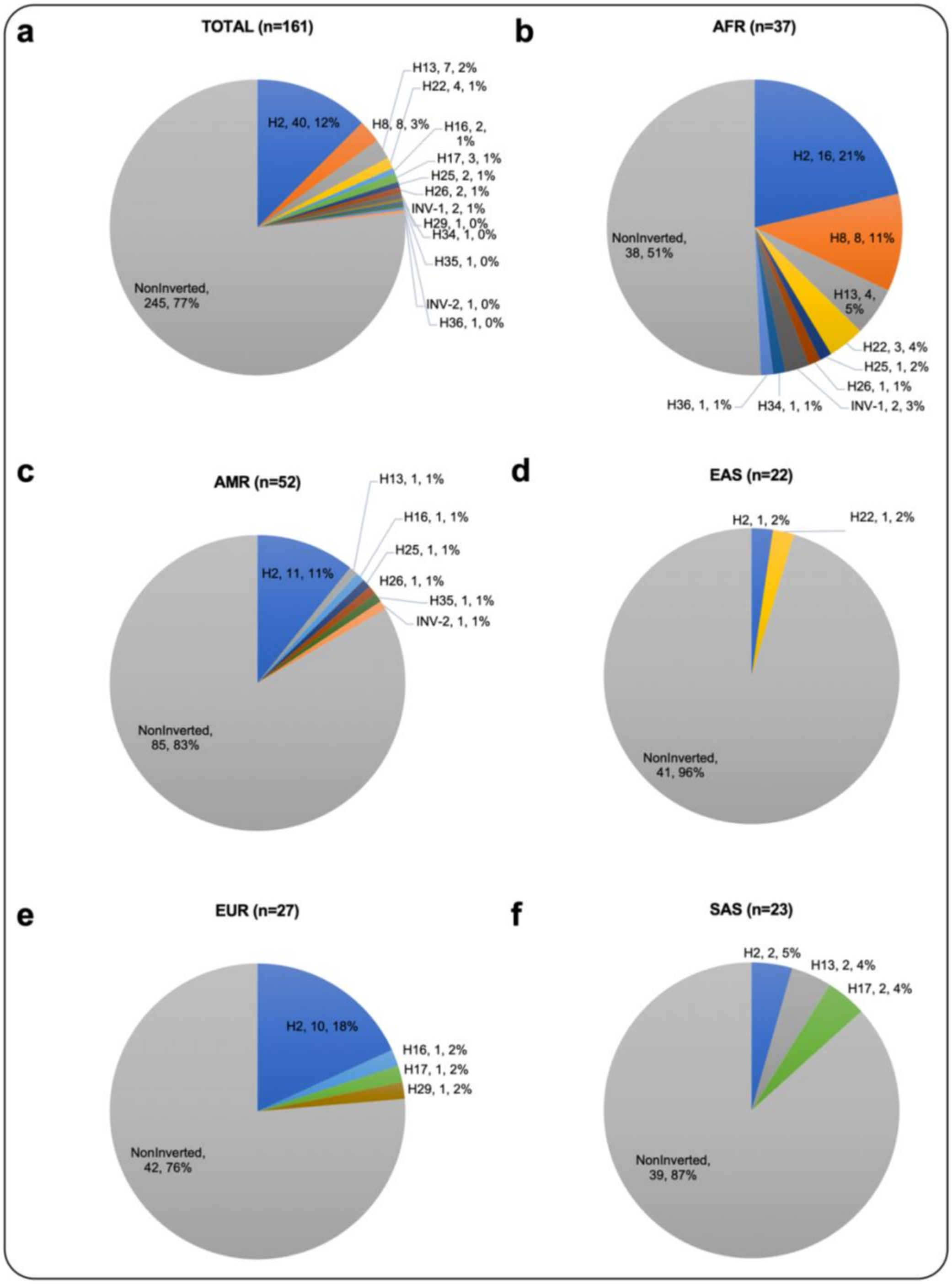

Figure S11 Inversion Frequencies. The proportion of inversion haplotypes: **a** among all studied samples **b** in the AFR population **c** in the AMR population. **d** in the EAS population **e** in the EUR population **f** in the SAS population. The labels in the pie charts indicate, in order, haplotype ID, count, percentage.

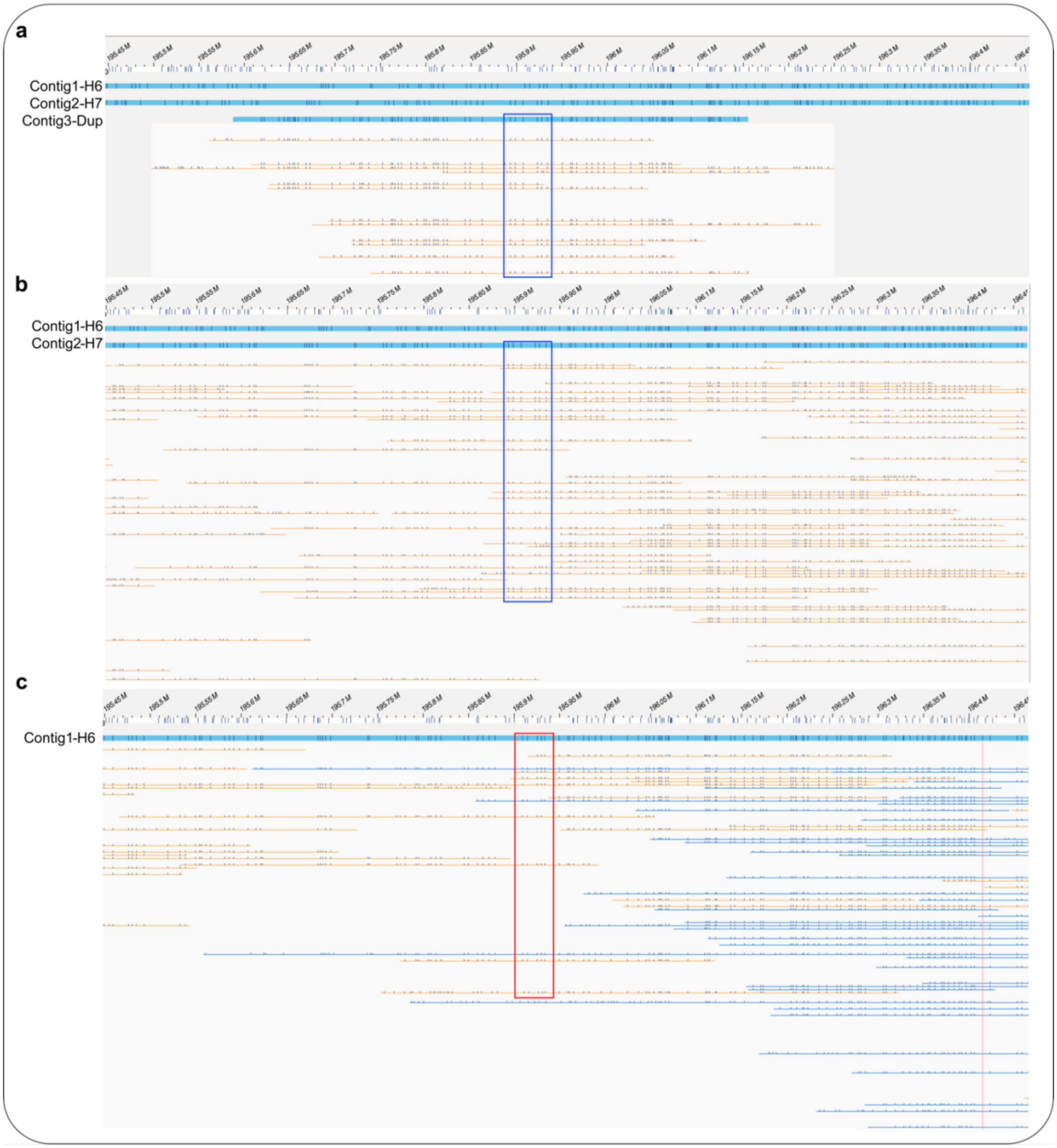

Figure S12 Molecule support for duplication in Family 19 – proband only sample. **a** Contigs identified in this sample, H7 with the duplication. Blue rectangle highlights single molecules supporting the duplication observed. **b** Duplication haplotype, H7, with molecules supporting the structure of duplicated region. **c** Haplotype, H6, without duplication, red rectangle highlighting the molecules which proves that H6 wasn't the duplicated haplotype.

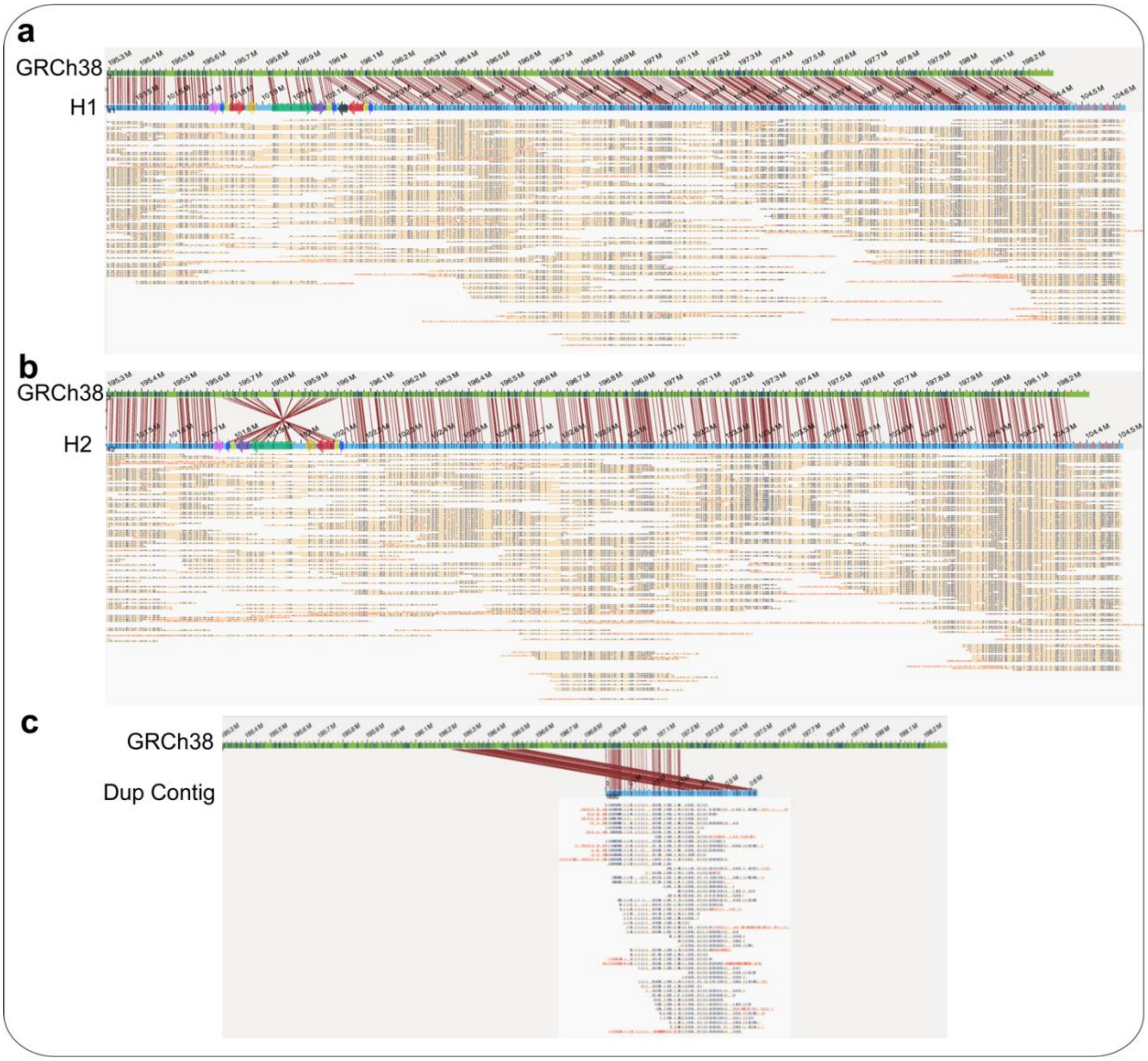

Figure S13 Molecule support for duplication in Family 12 – proband only sample. **a** H1 haplotype and molecules supporting the haplotype structure. **b** H2 haplotype and molecules supporting the haplotype structure. **c** Duplication contig and molecules supporting the haplotype structure. Green horizontal rectangle, GRCh38 *in silico* map; blue horizontal rectangle, haplotype contigs; yellow horizontal lines with dark blue labels, OM single molecules.

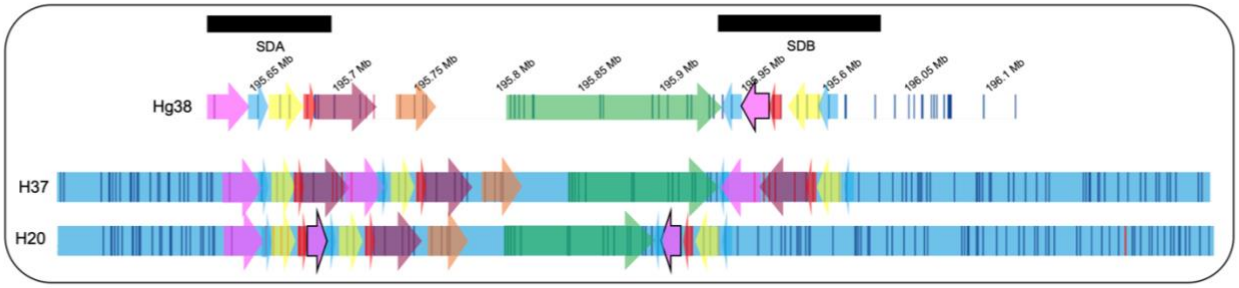

Figure S14 Novel 3q29 haplotype. OM contigs in blue rectangles with vertical dark lines. Colored arrows represent the 3q29 segments in each haplotype.

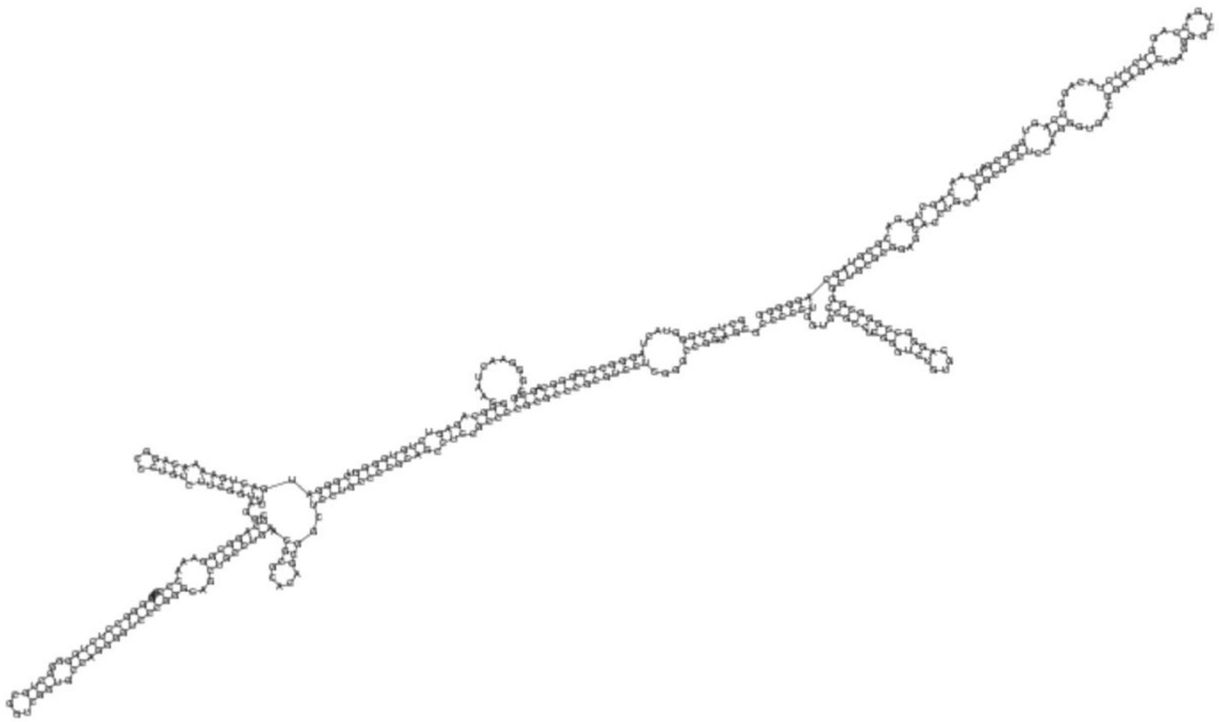

Figure S15 Predicted secondary structure of the breakpoint. Breakpoint junction (374 bp) identified in Family 15 proband with the 3q29 deletion syndrome used for secondary structure prediction.

References

1. Altschul SF, Gish W, Miller W, Myers EW, Lipman DJ. Basic local alignment search tool. J Mol Biol. 1990 Oct 5;215(3):403–10.

2. Camacho C, Coulouris G, Avagyan V, Ma N, Papadopoulos J, Bealer K, et al. BLAST+: architecture and applications. BMC Bioinformatics. 2009 Dec 15;10(1):421.

3. Chen Y, Ye W, Zhang Y, Xu Y. High speed BLASTN: an accelerated MegaBLAST search tool. Nucleic Acids Res. 2015 Sep 18;43(16):7762–8.

4. Cheng H, Concepcion GT, Feng X, Zhang H, Li H. Haplotype-resolved de novo assembly using phased assembly graphs with hifiasm. Nat Methods. 2021 Feb;18(2):170–5.

5. Heller D, Vingron M. SVIM-asm: Structural variant detection from haploid and diploid genome assemblies. Bioinformatics [Internet]. 2020 Dec 21; Available from: http://dx.doi.org/10.1093/bioinformatics/btaa1034
