# Supplementary material for: High level of complexity and global diversity of the 3q29 locus revealed by optical mapping and long-read sequencing
Source: Genome Med. 2023 May 10;15:35. doi: 10.1186/s13073-023-01184-5 (PMC10170684; doi:10.1186/s13073-023-01184-5)
Supplement: Supplementary file 3 — Additional file 3. Molecule support for the 3q29 Project samples. [file 13073_2023_1184_MOESM3_ESM.pdf]

- Family 1 - Proband
- Family 1 - Father
- Family 1 - Mother
- Family 2 - Proband
- Family 2 - Father
- Family 2 - Mother
- Family 3 - Proband
- Family 4 - Proband
- Family 6 - Proband
- Family 6 - Mother
- Family 7 - Proband
- Family 7 - Father
- Family 7 - Mother
- Family 9 - Proband
- Family 9 - Father
- Family 9 - Mother
- Family 10 - Proband
- Family 10 - Father
- Family 10 - Mother
- Family 11 - Proband
- Family 11 - Father
- Family 11 – Mother
- Family 12 – Proband
- Family 13 - Proband
- Family 13 - Father
- Family 13 - Mother
- Family 14 - Proband
- Family 14 - Sibling
- Family 14 - Father
- Family 14 - Mother
- Family 15 - Proband
- Family 15 - Father
- Family 15 - Mother
- Family 16 - Proband
- Family 16 - Father
- Family 16 - Mother
- Family 17 – Proband
- Family 17 – Father
- Family 18 – Proband
- Family 18 – Father
- Family 19 – Proband
- Family 20 – Proband
- Family 20 – Sibling
- Family 20 – Sibling
- Family 20 – Father
- Family 20 – Mother

Family 1 - Trio

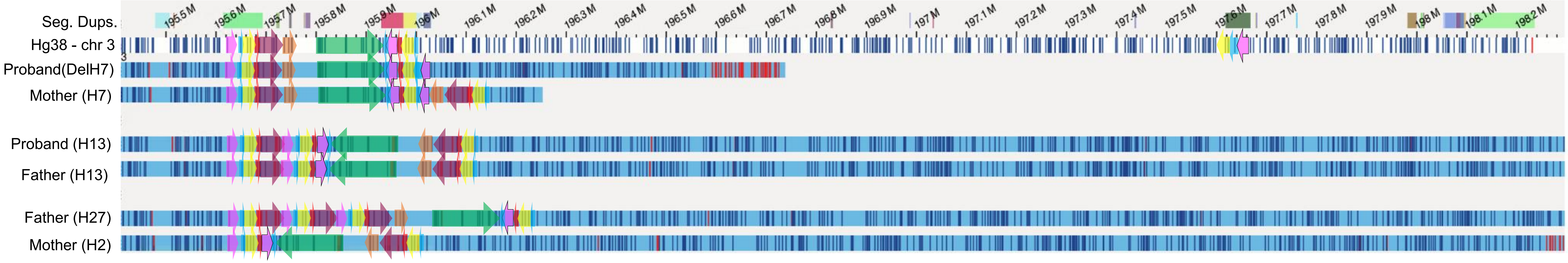

Go to the first page

Family 1 - Proband

H7

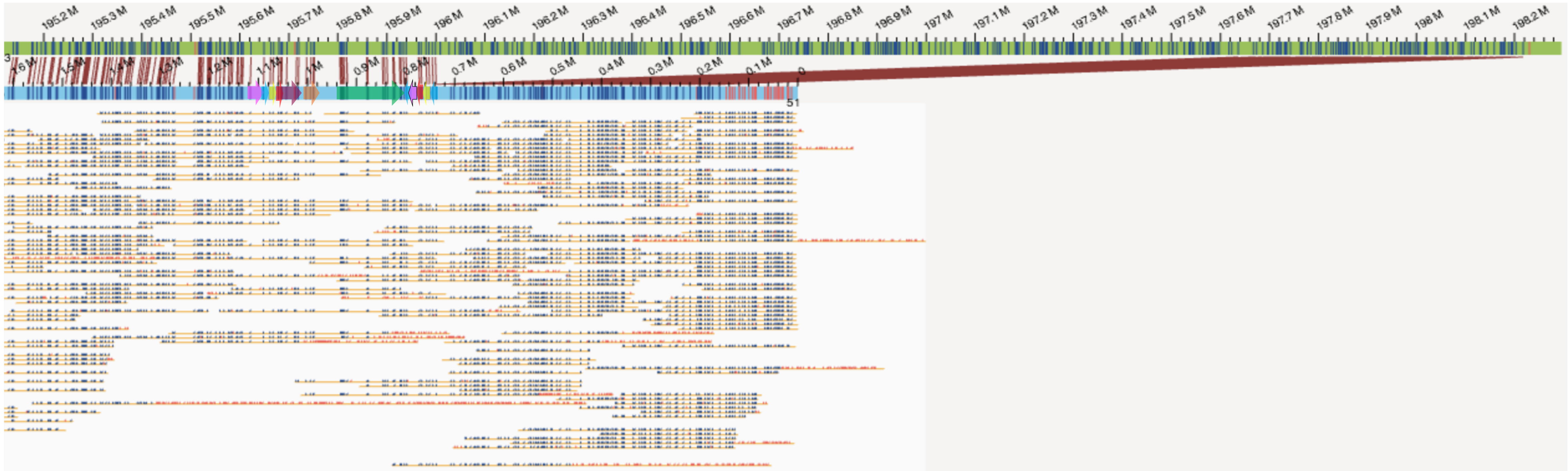

H13

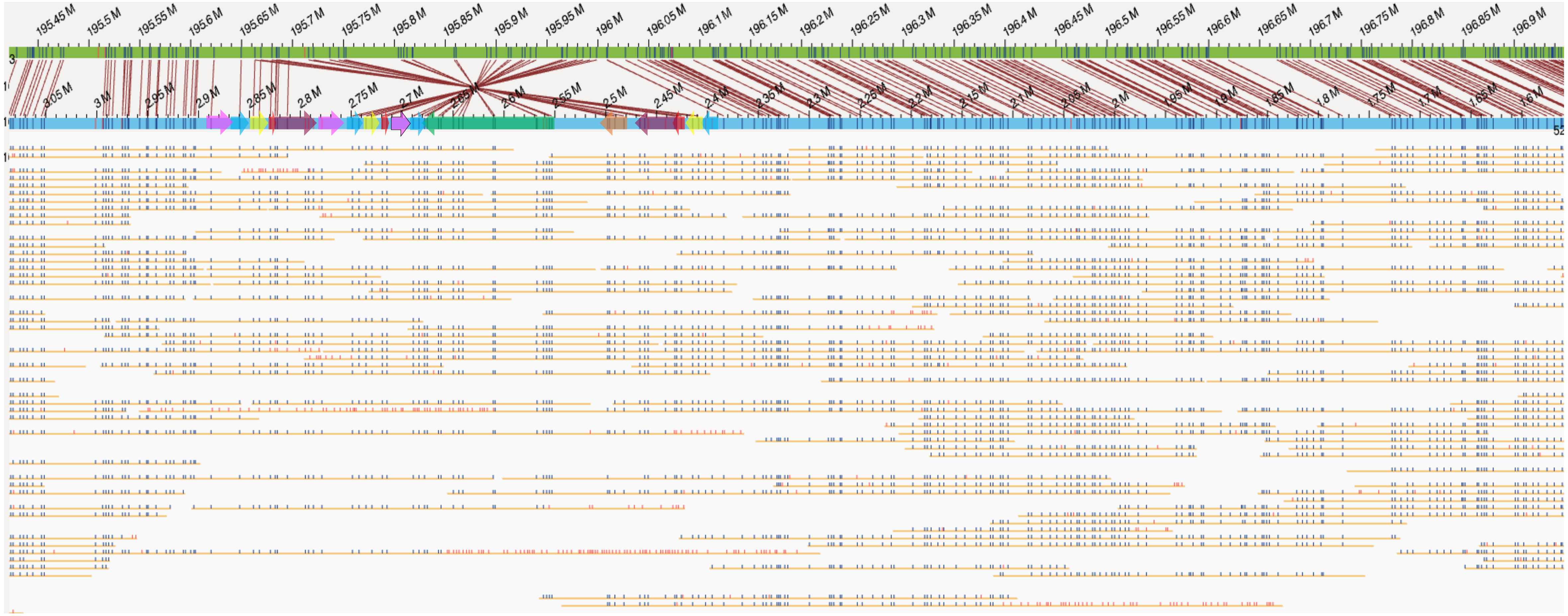

Go to the first page

Family 1 - Father

H13

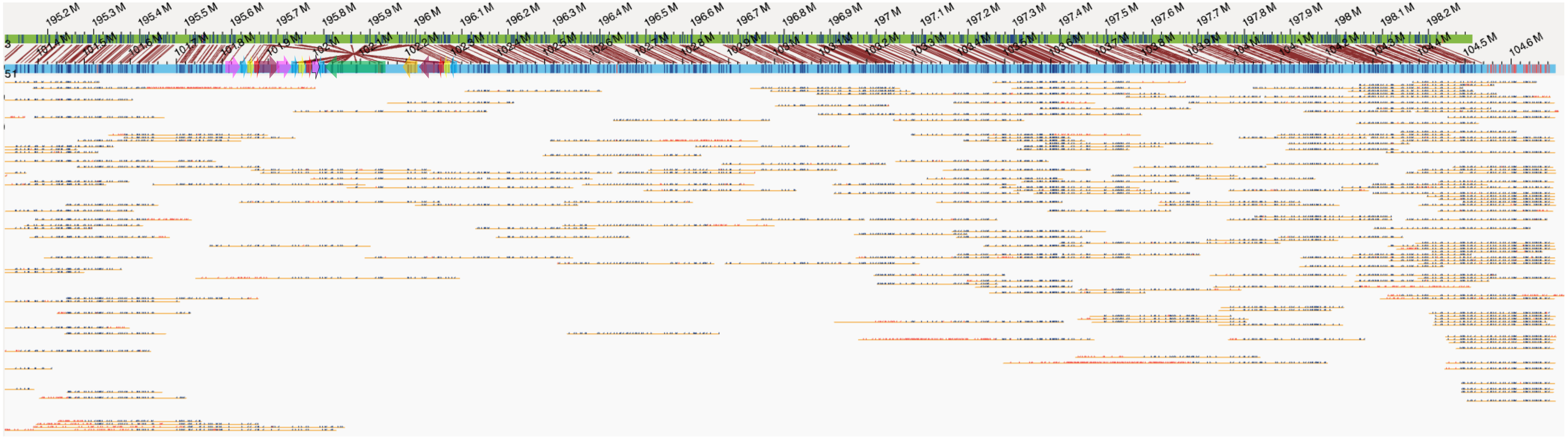

H27

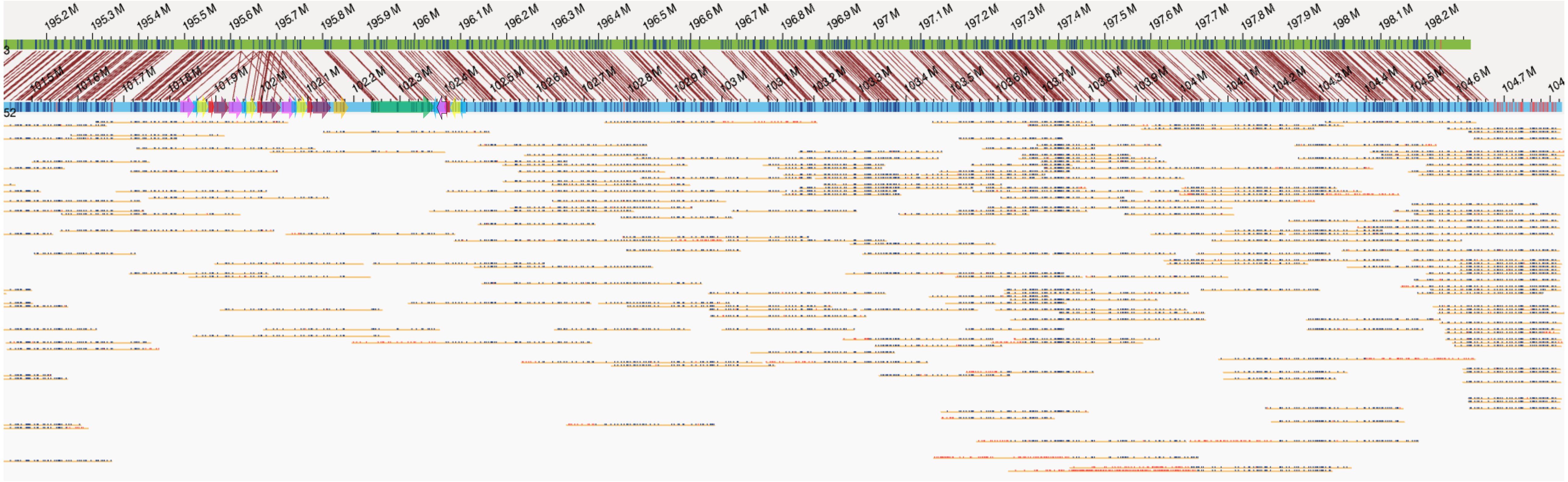

Go to the first page

Family 1 - Mother

H2

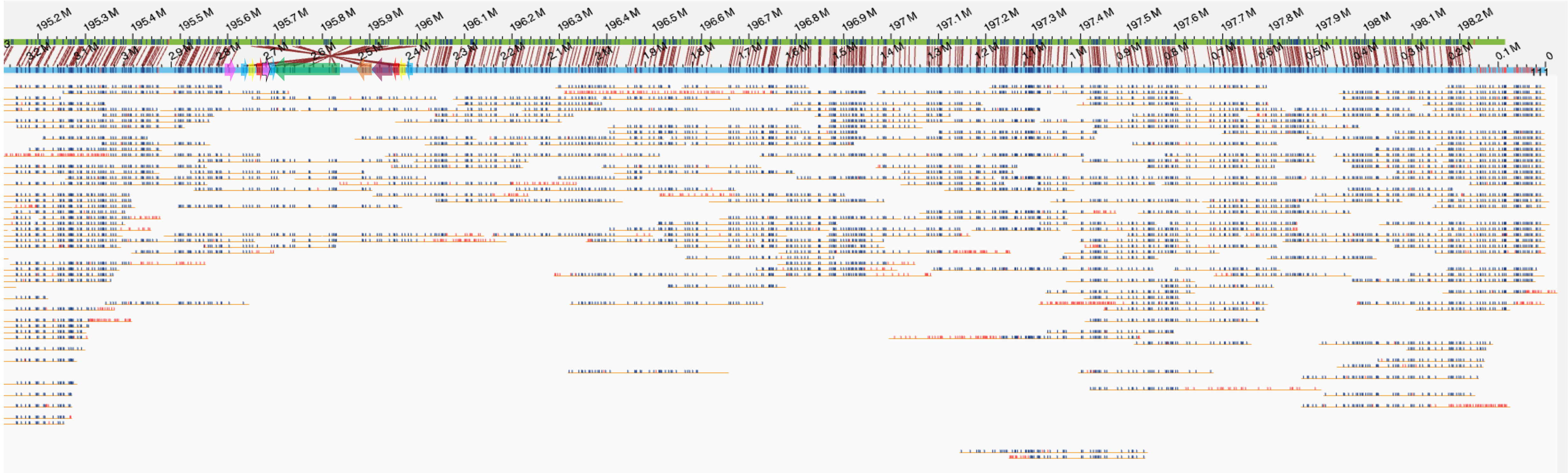

H7

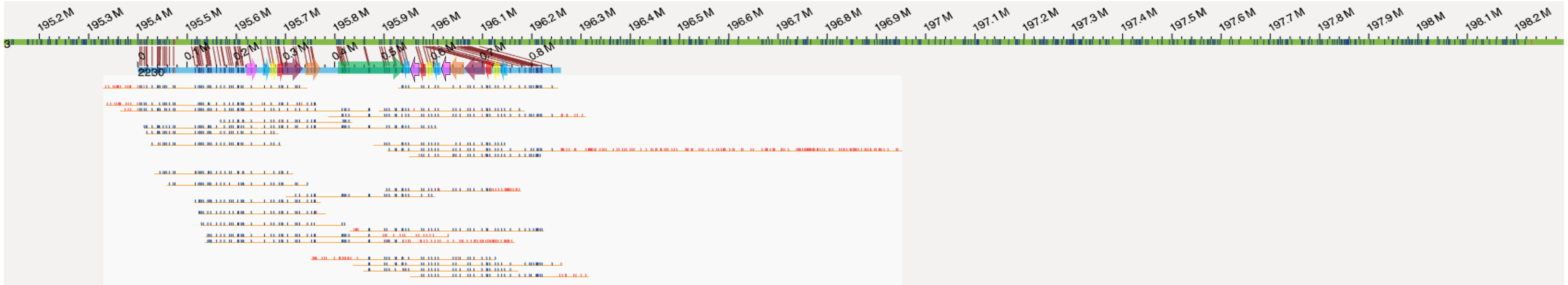

Go to the first page

Family 2 - Trio

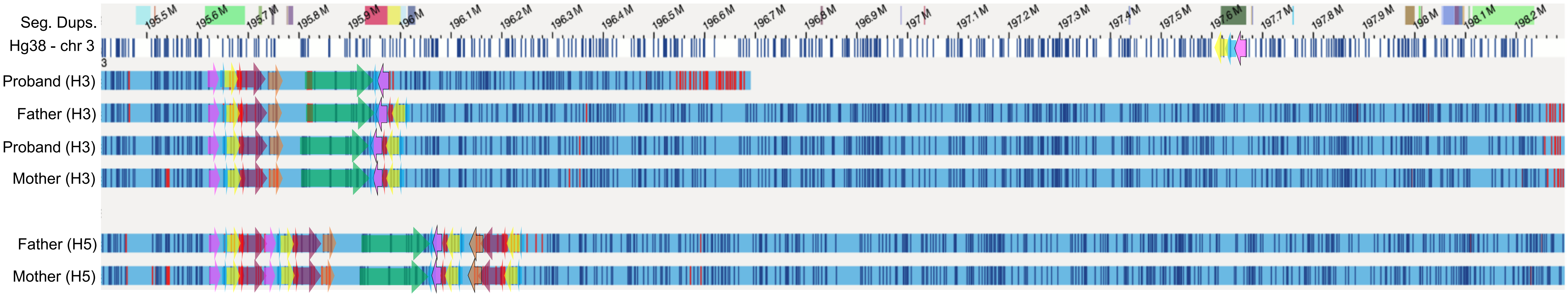

Go to the first page

Family 2 - Proband

H3

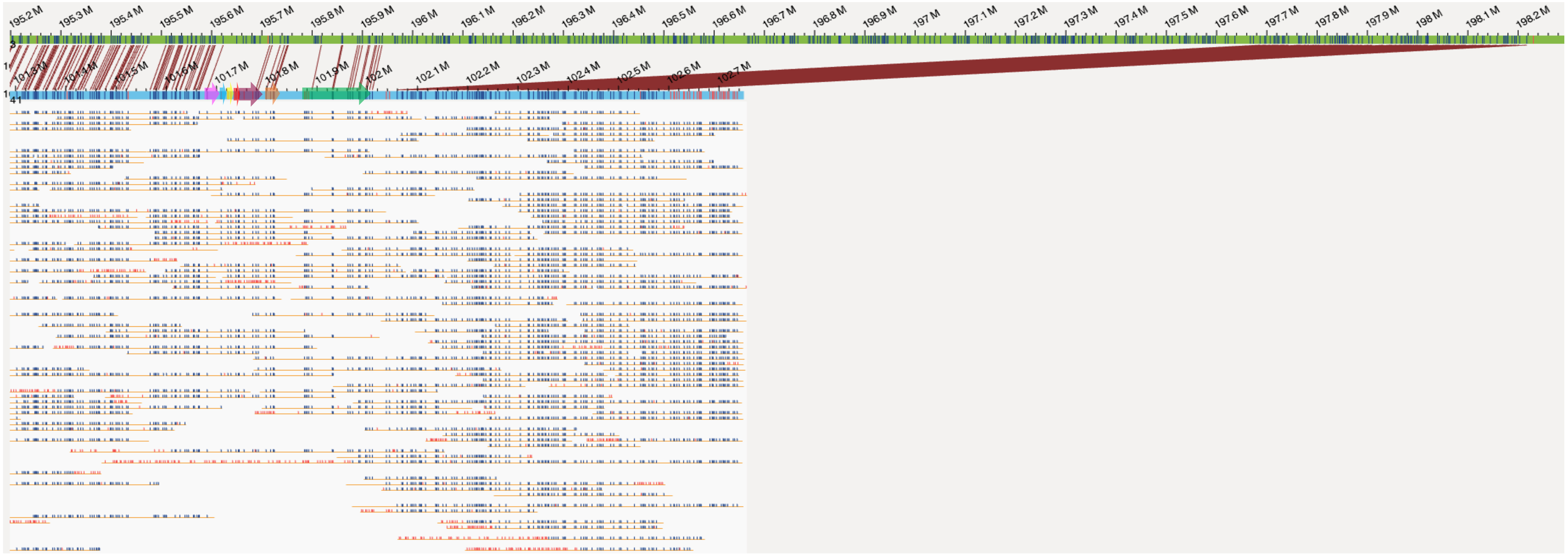

H3

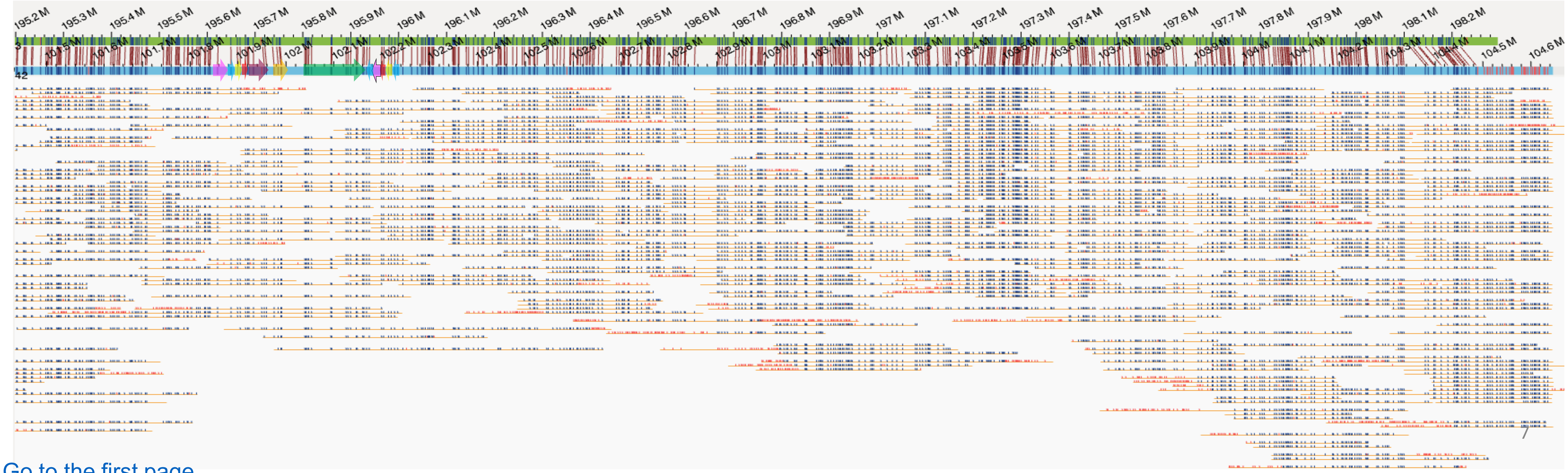

Go to the first page

Family 2 - Father

H3

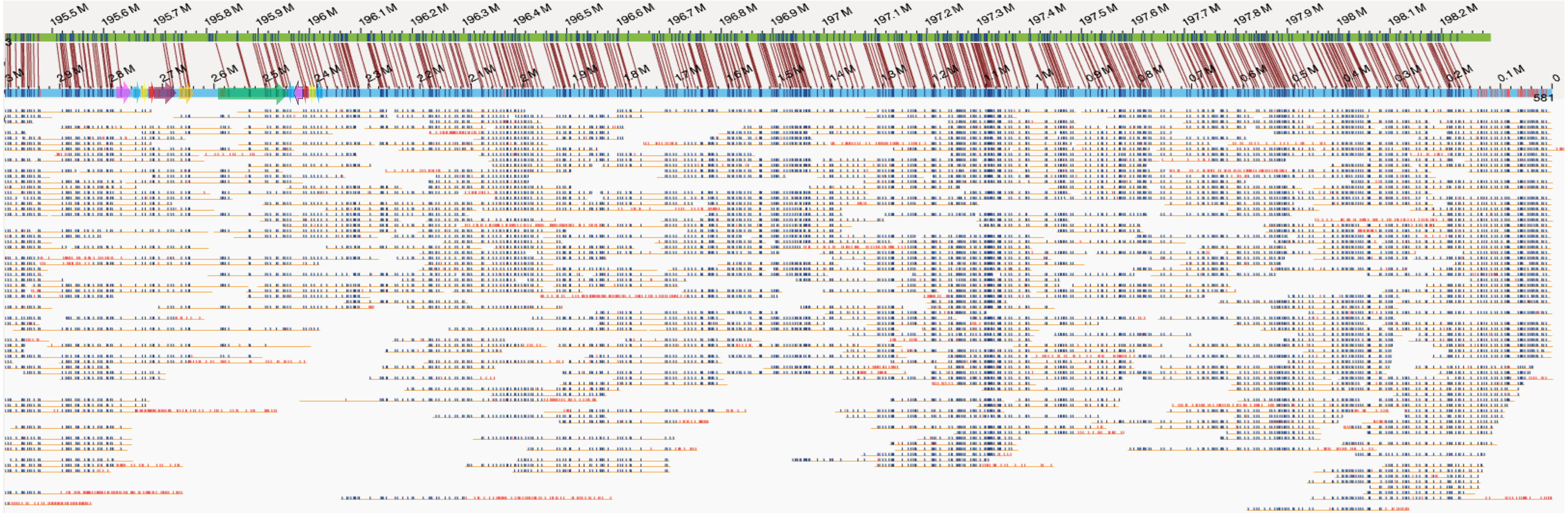

H5

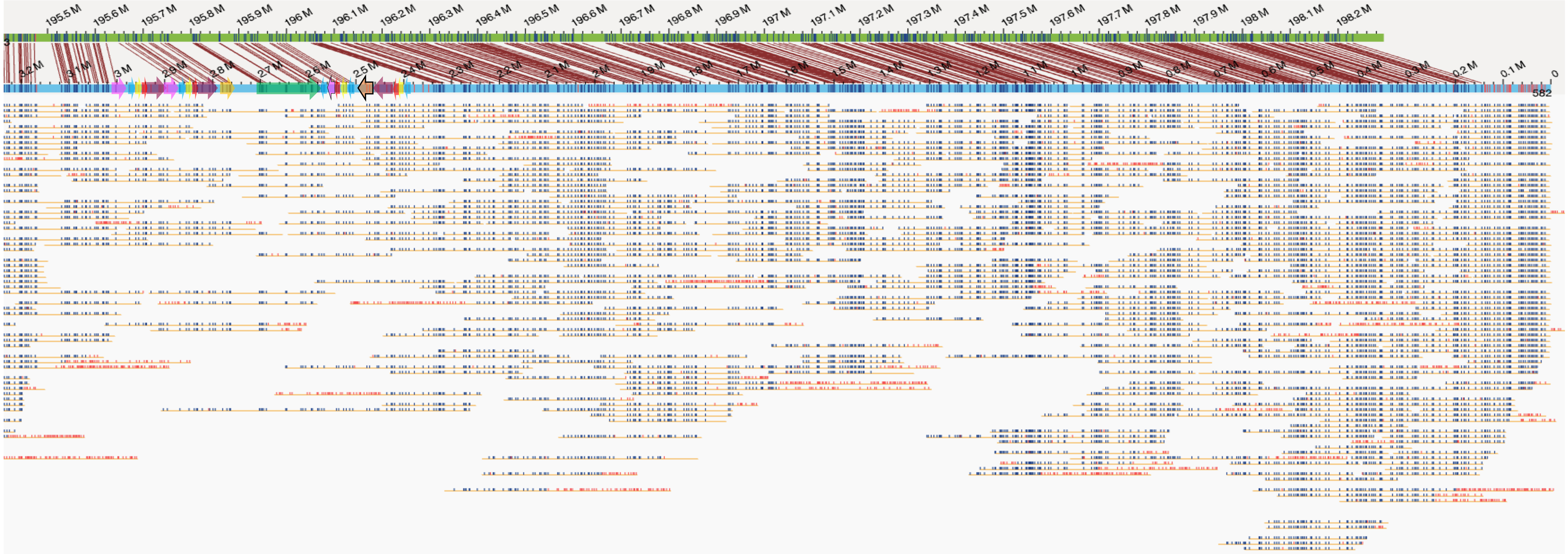

Go to the first page

Family 2 - Mother

H5

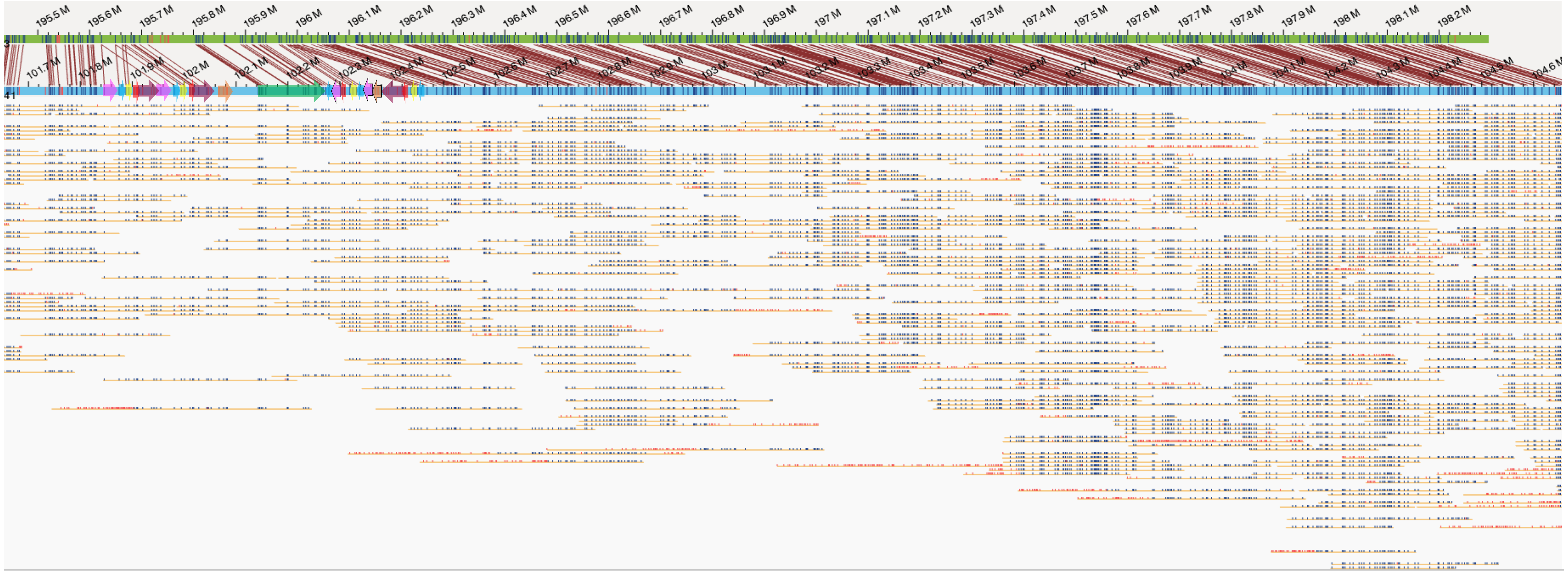

H3

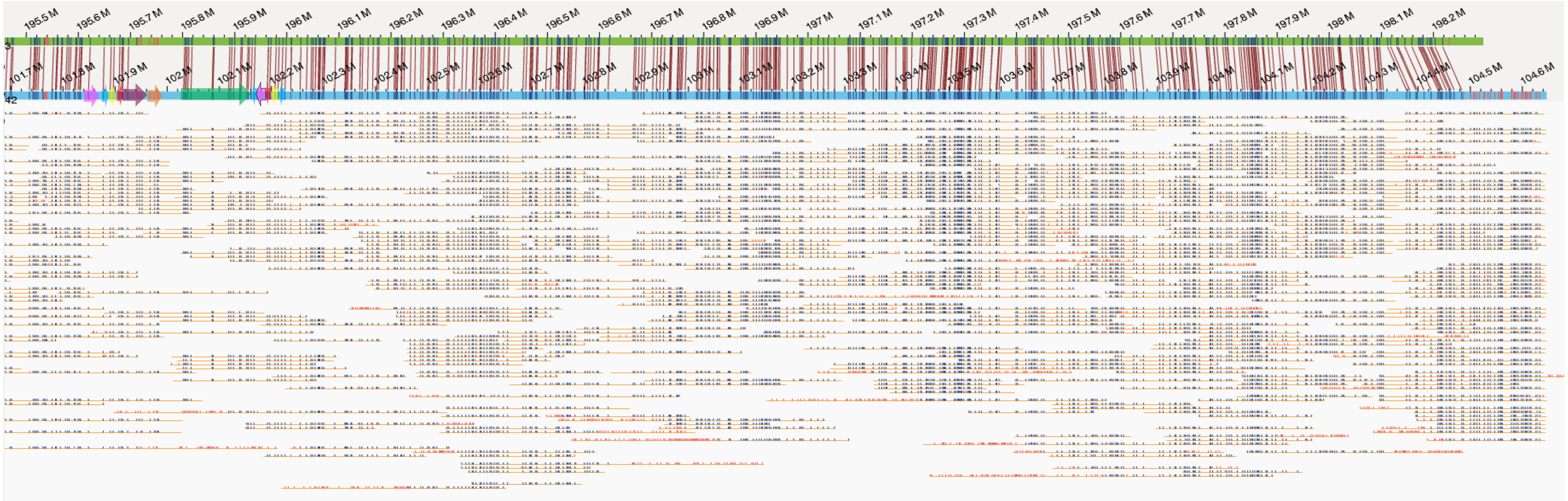

Go to the first page

Family 3 – Proband Only

NA-H1,3,7,9,10,12

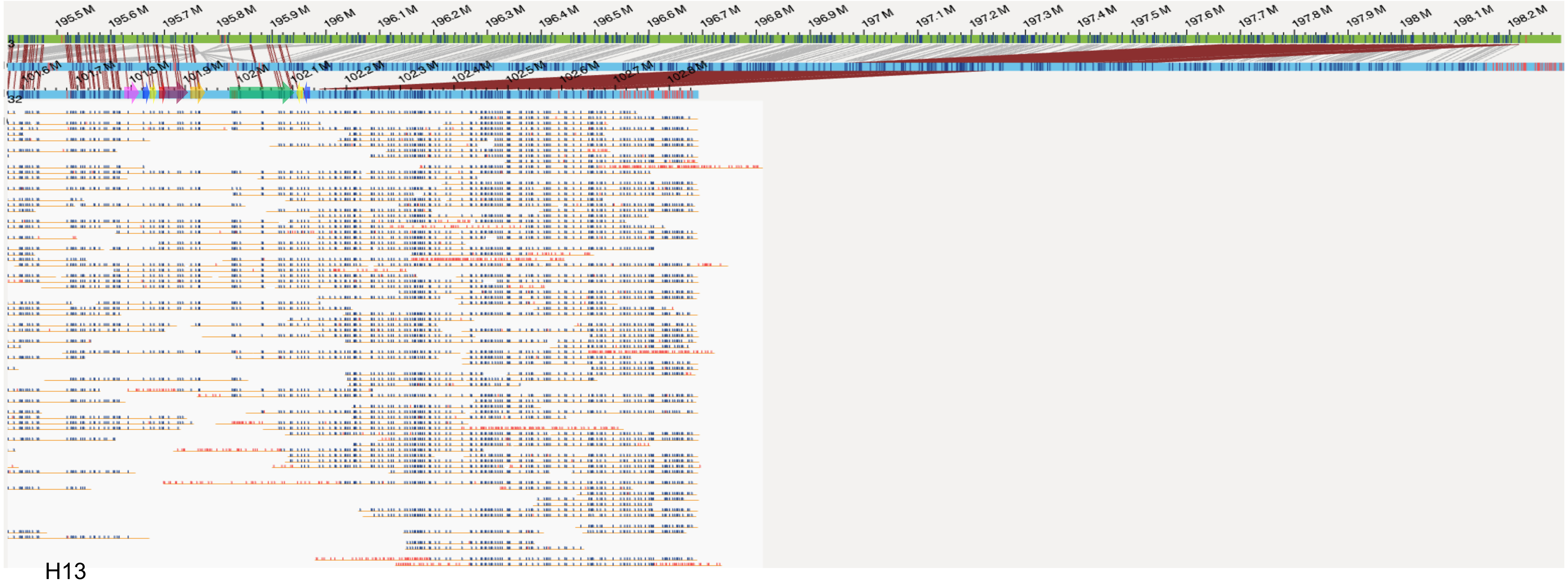

H13

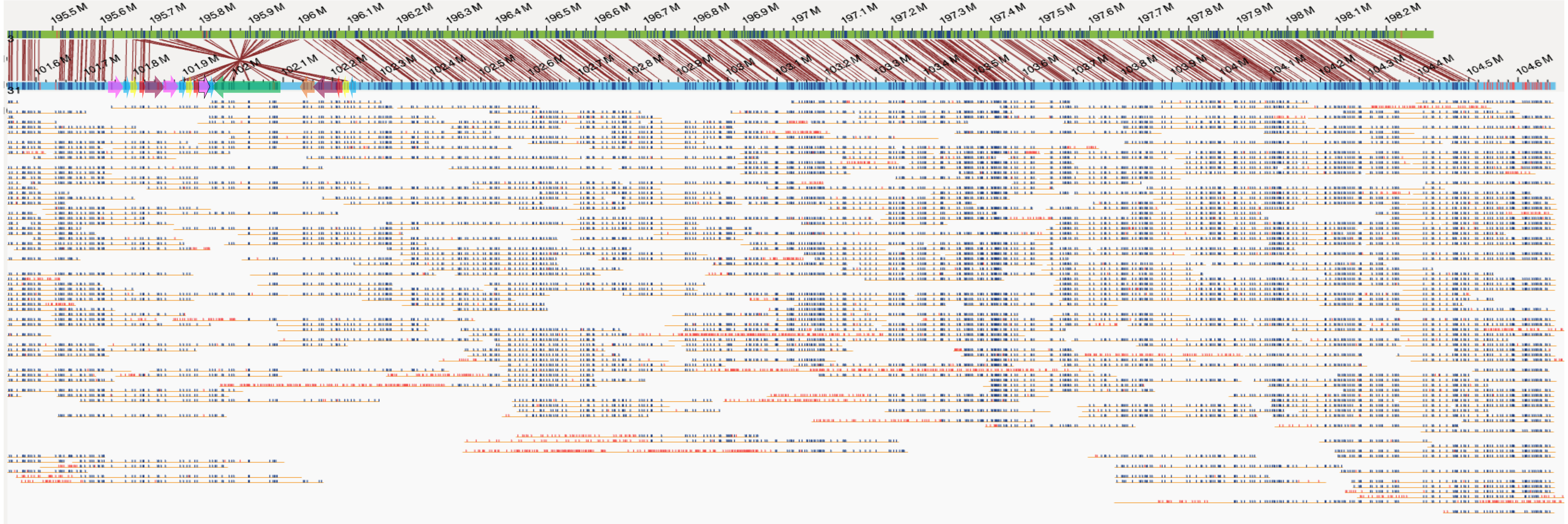

Go to the first page

Family 4 – Proband Only

NA-H1,3,7,9,10,12

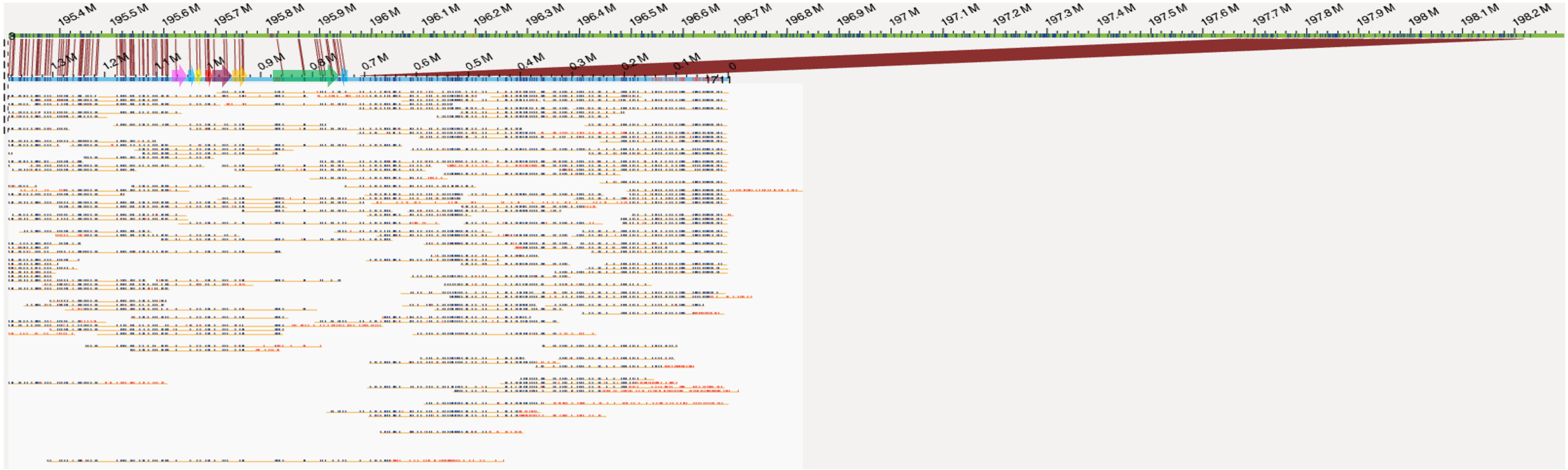

H2

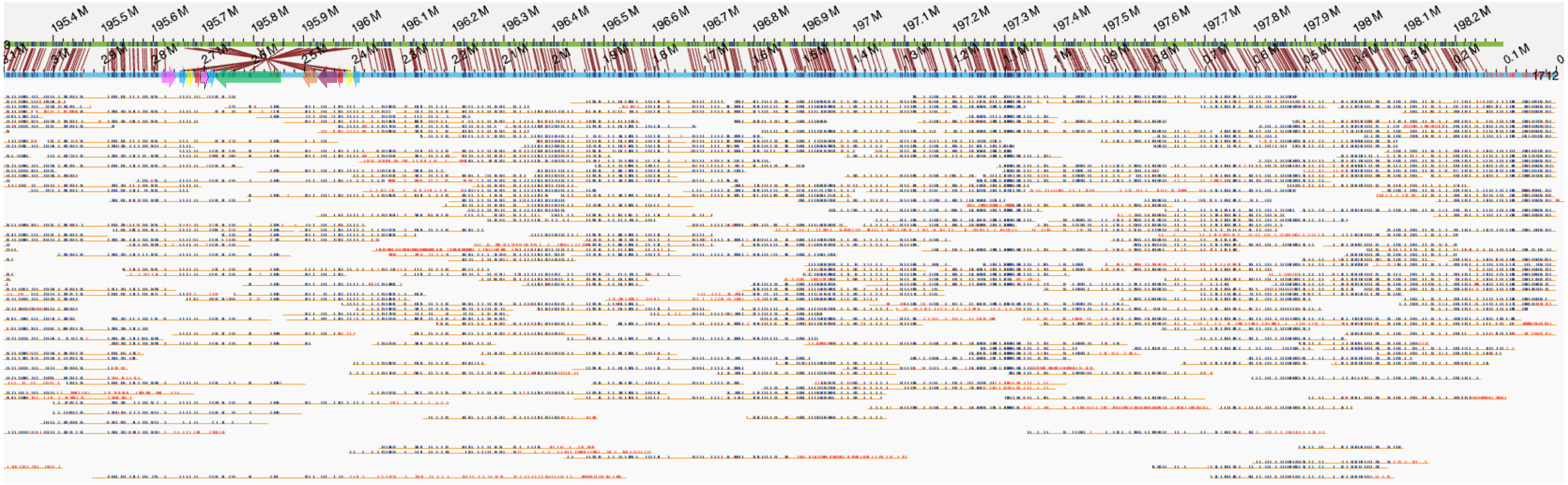

Go to the first page

Family 6 – Proband & Mother

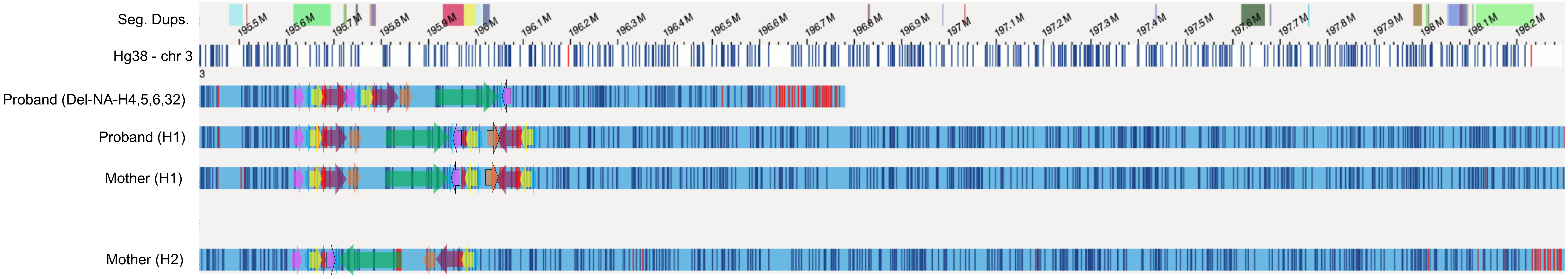

Go to the first page

Family 6 - Proband

NA-H4,5,6

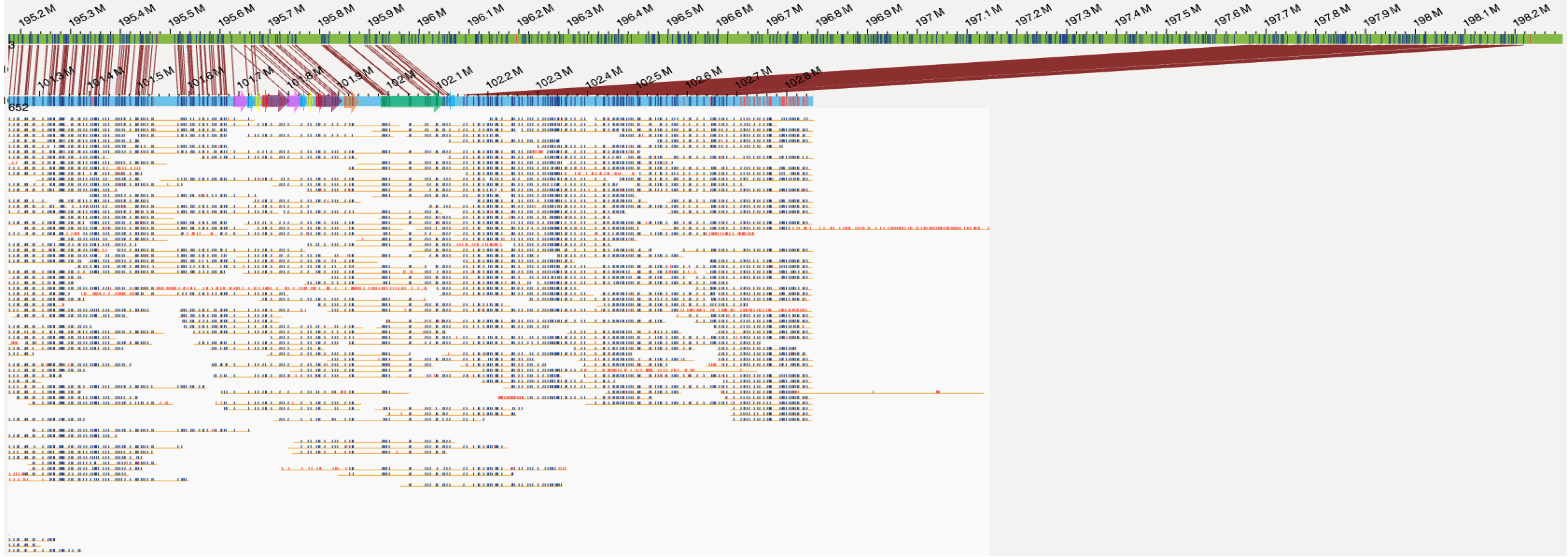

H1

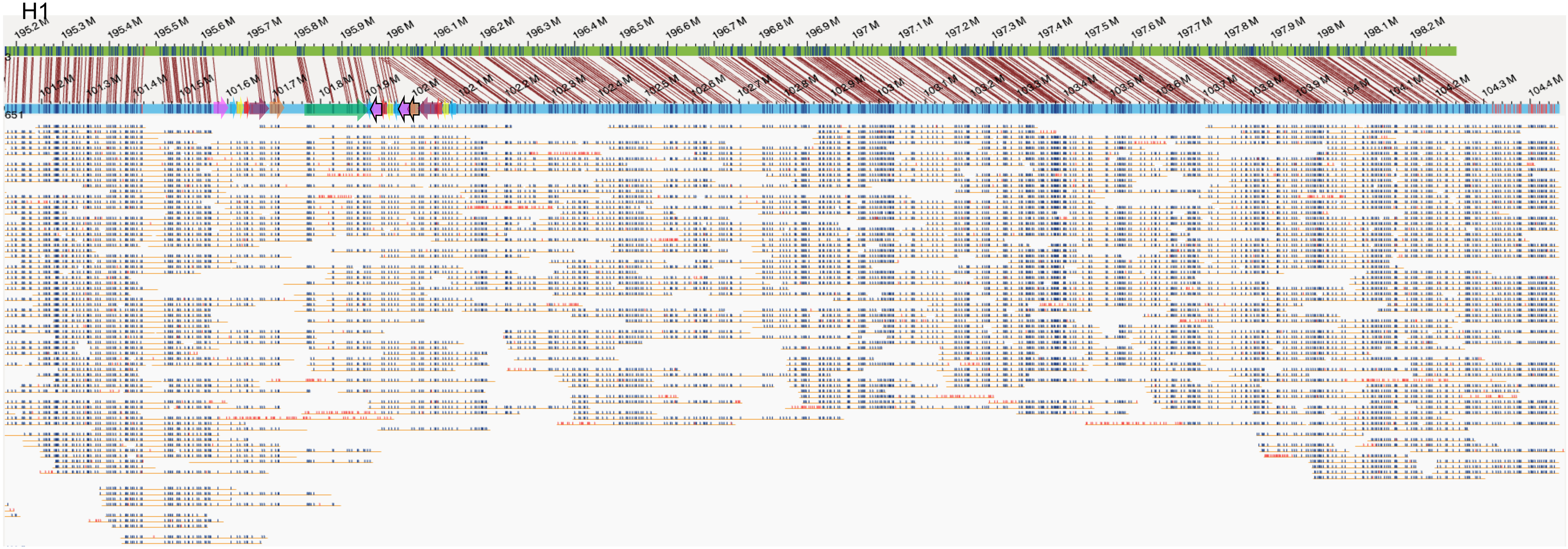

Go to the first page

Family 6 - Mother

H1

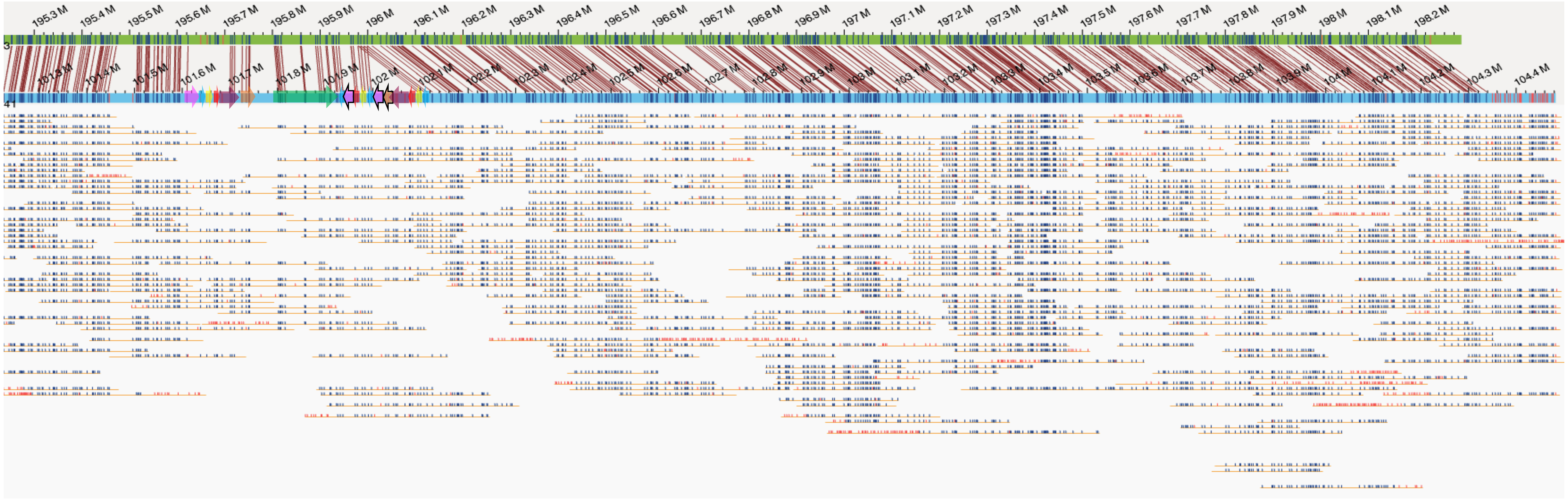

H2

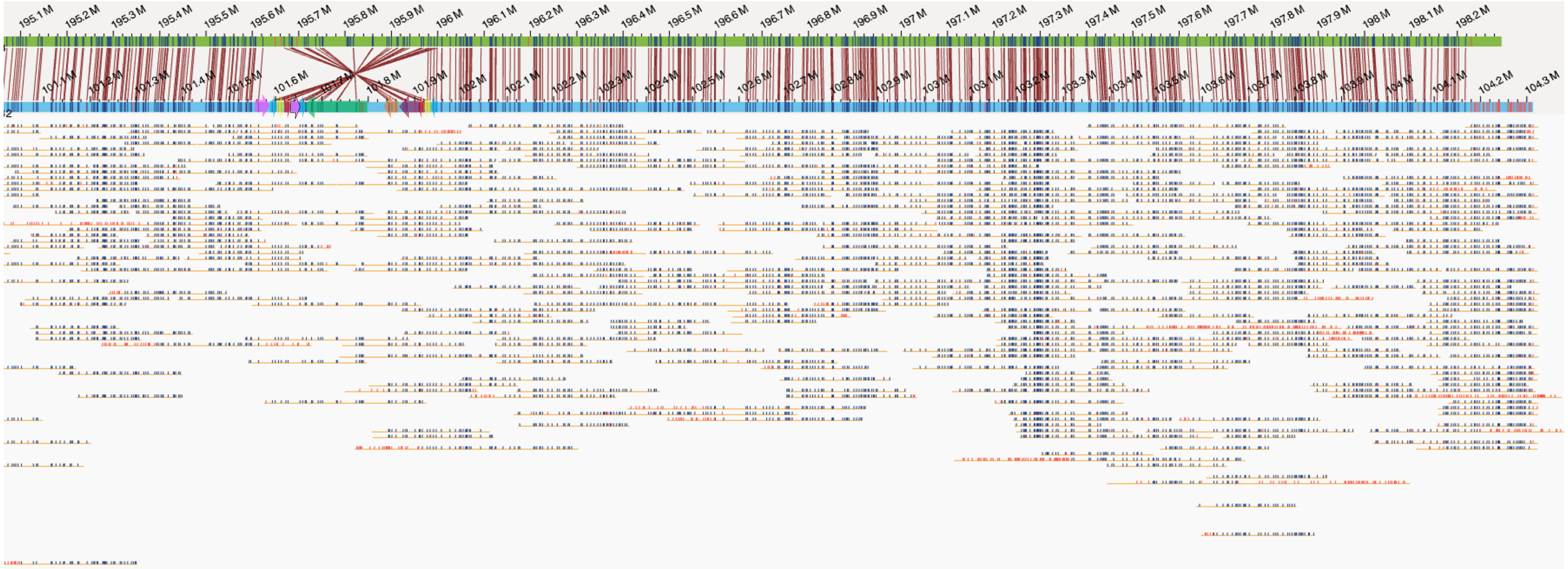

Go to the first page

Family 7

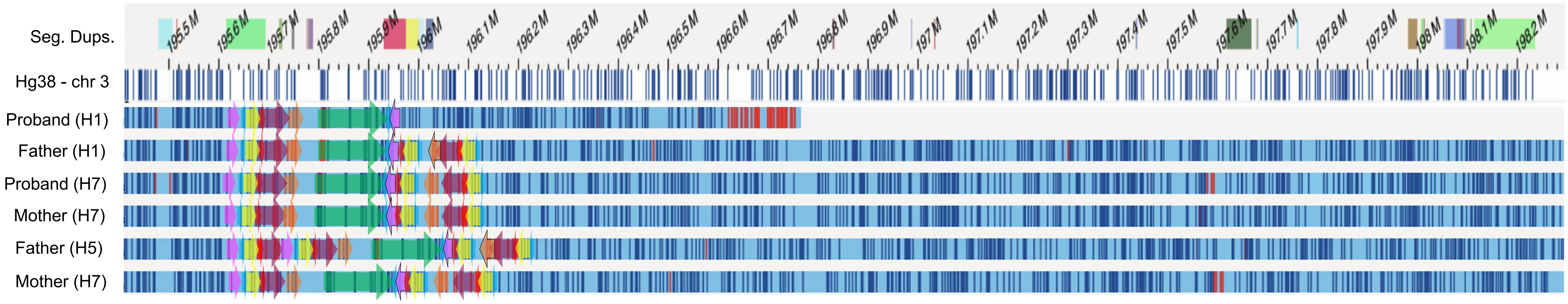

Go to the first page

Family 7 – Proband – Part 1

H1

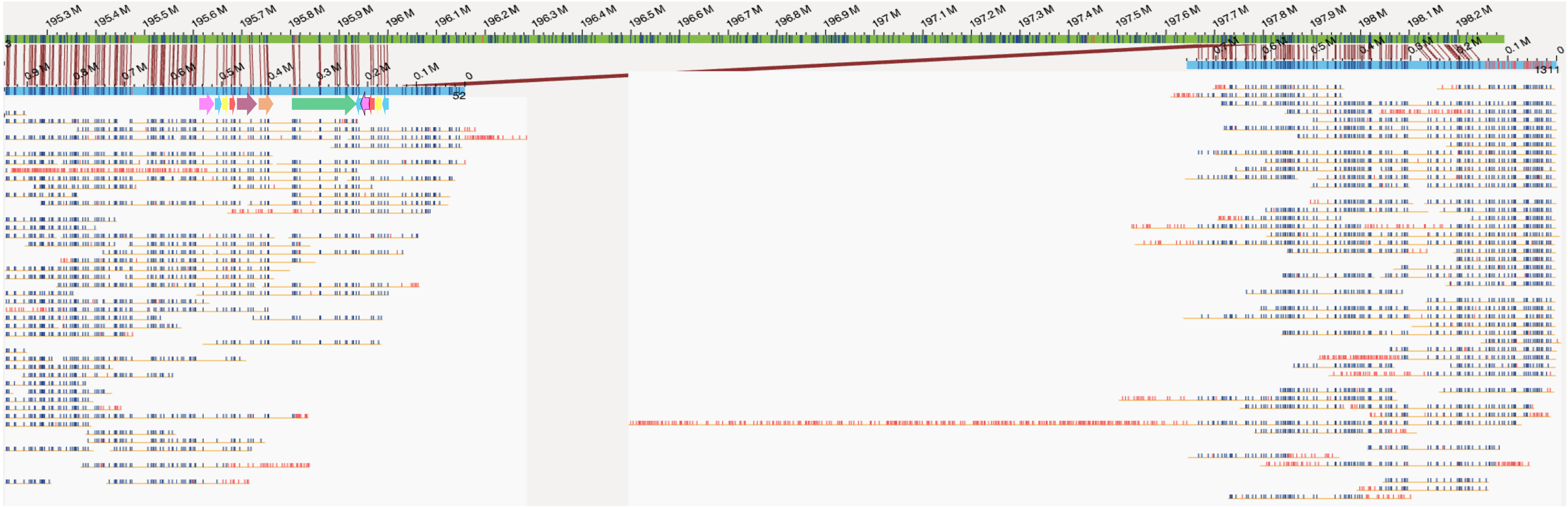

Go to the first page

Family 7 – Proband – Part 2

H7

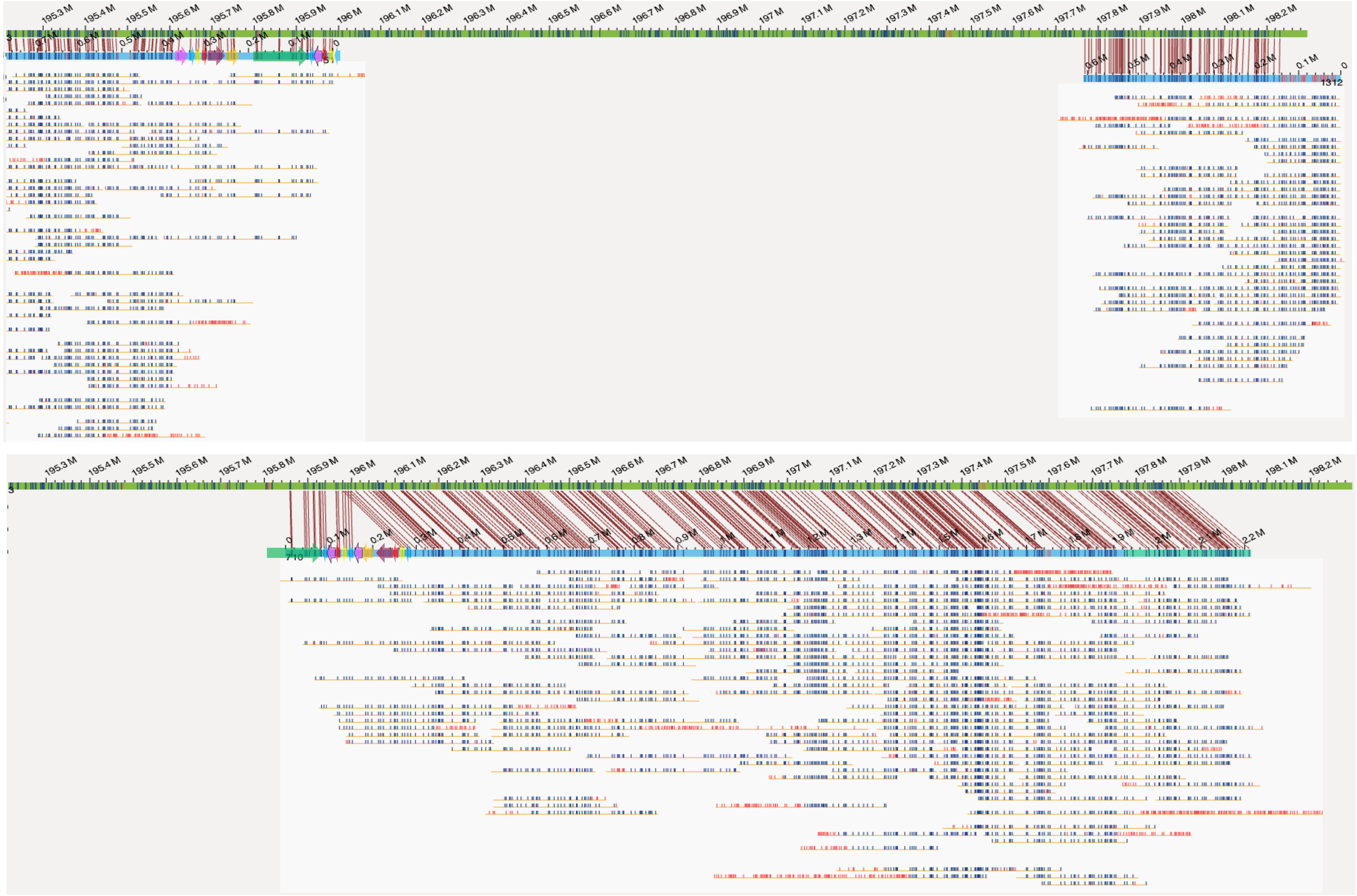

Go to the first page

Family 7 – Father

H5

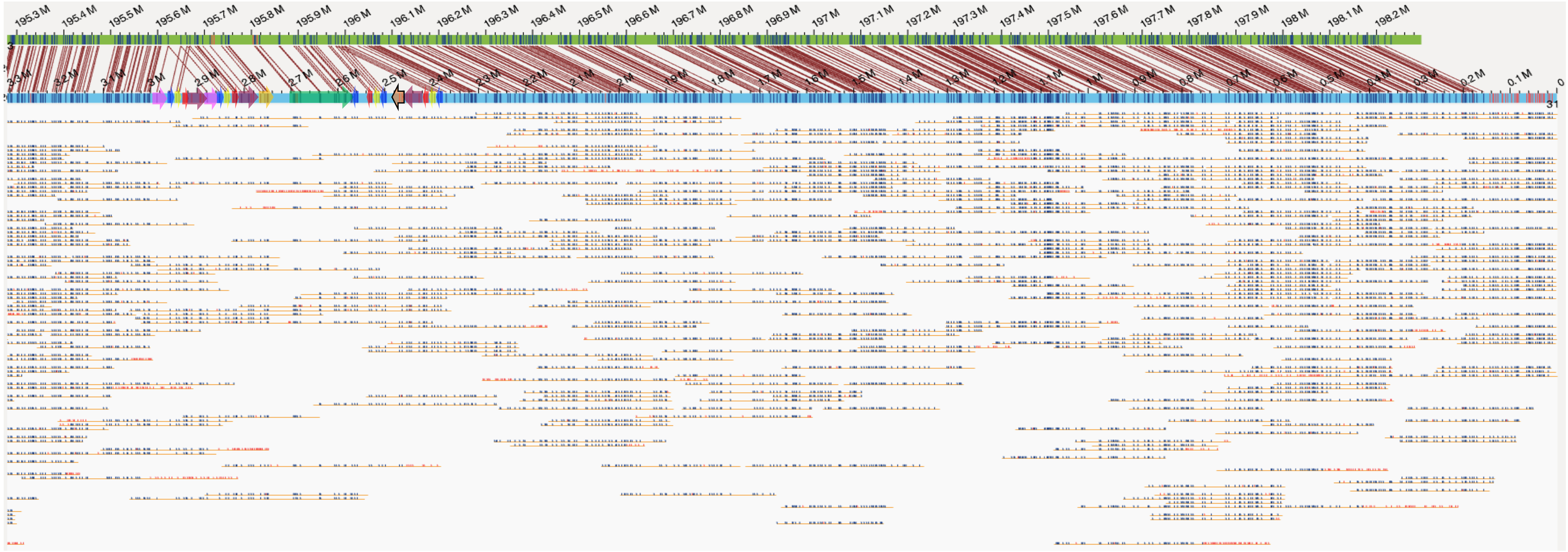

H1

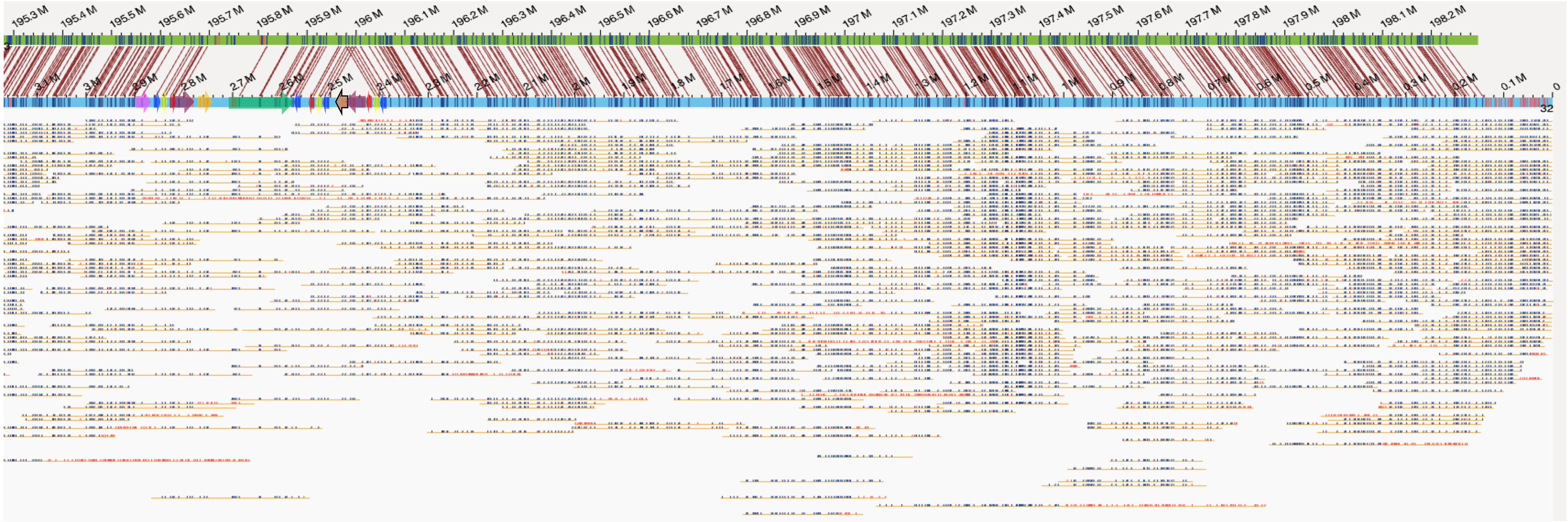

Go to the first page

Family 7 – Mother

H7

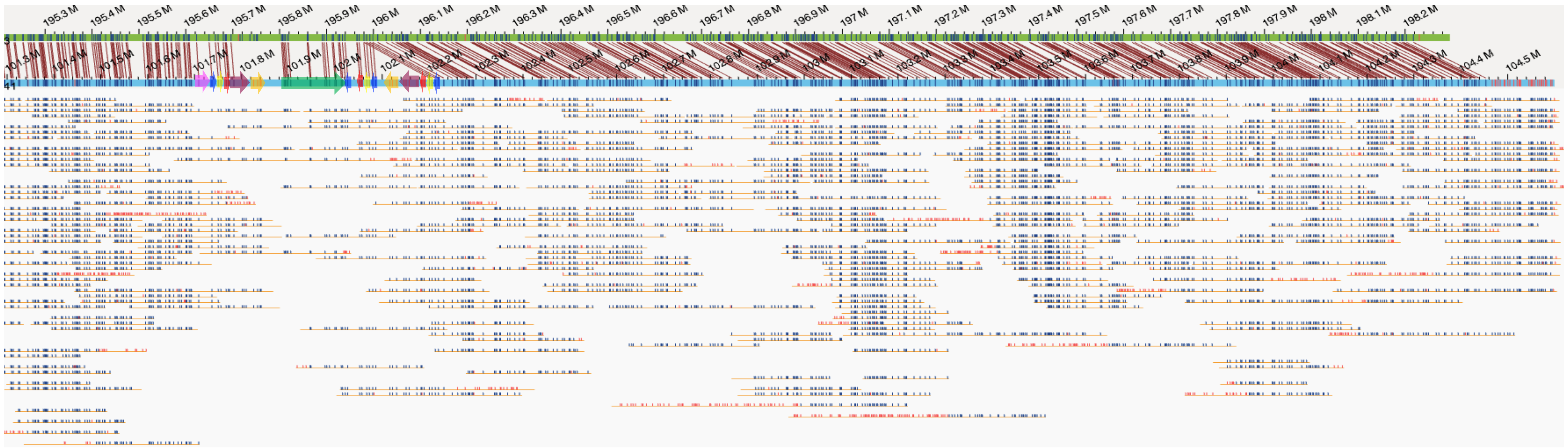

H7

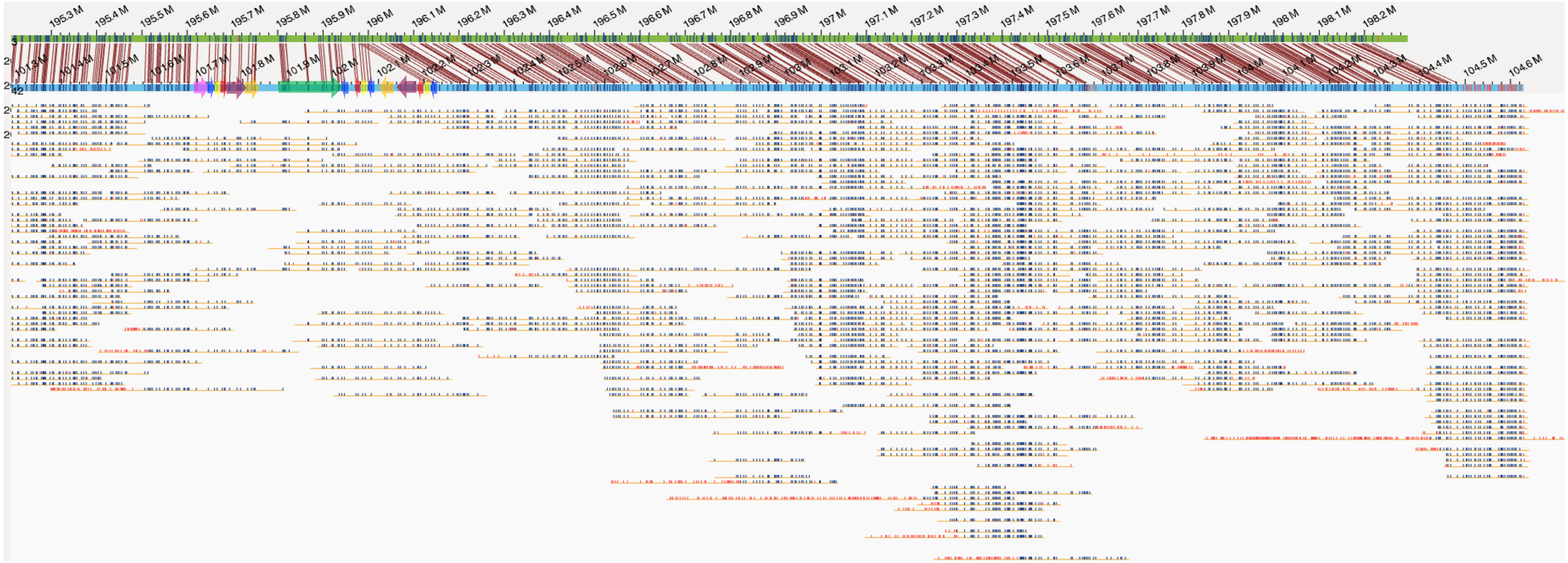

Go to the first page

Family 9 - Trio

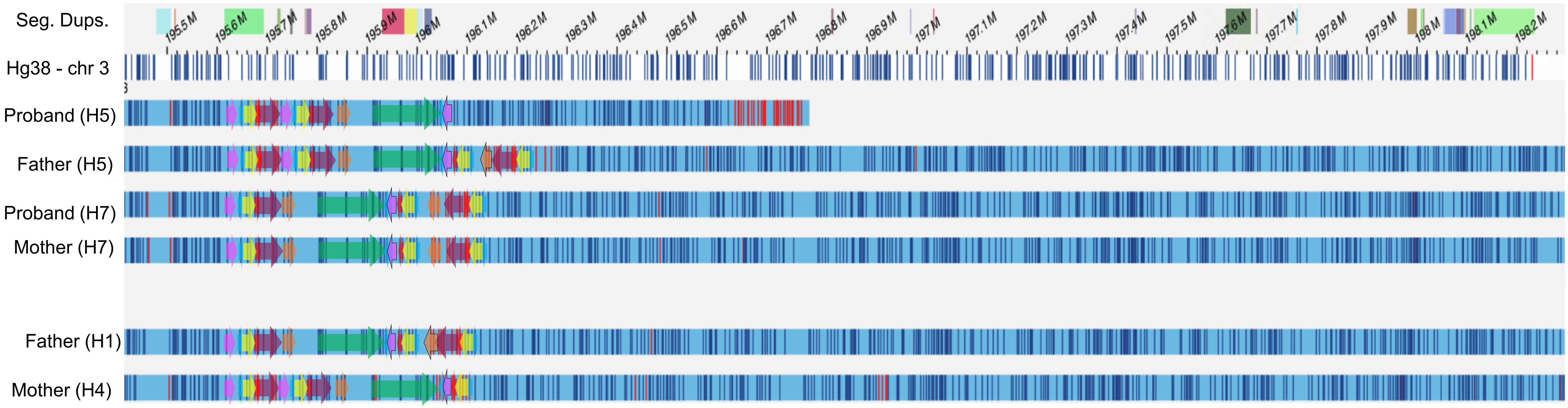

Go to the first page

Family 9 - Proband

H5

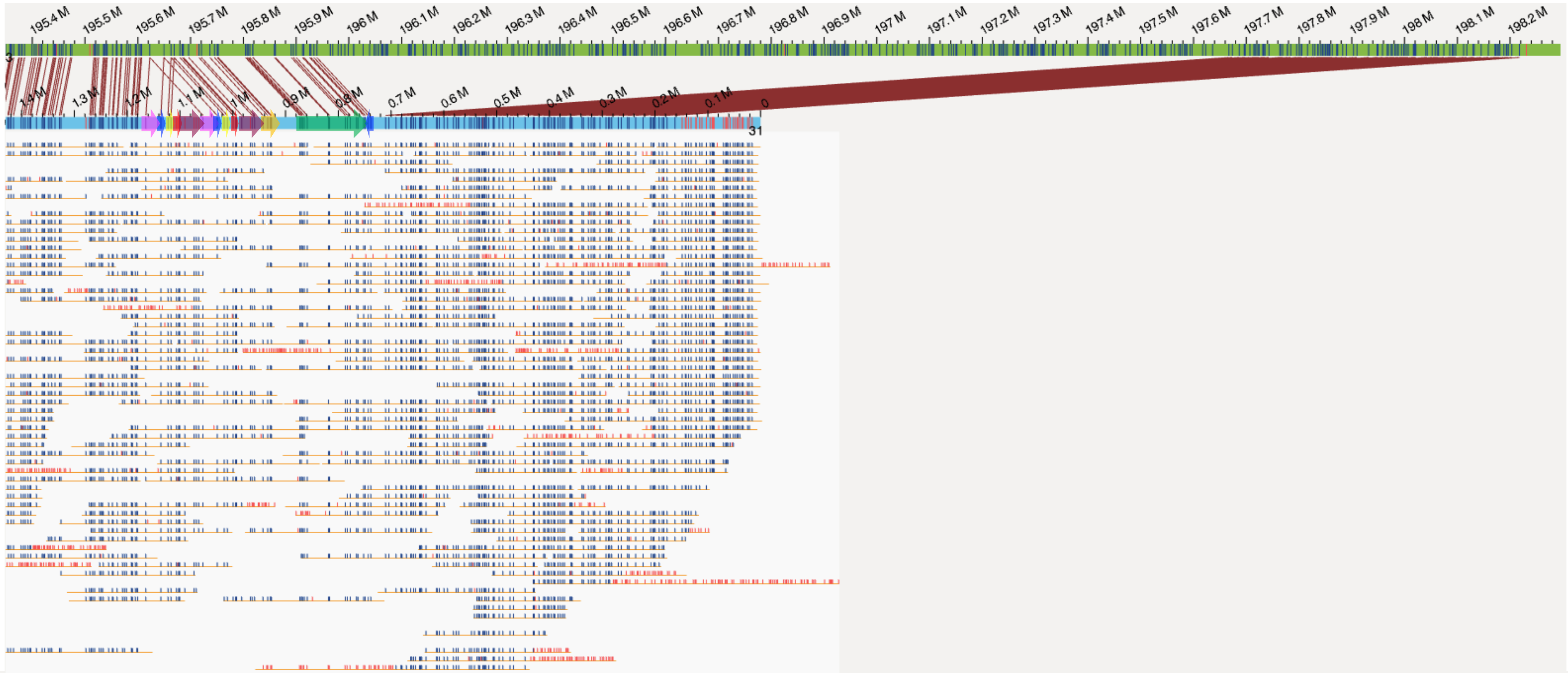

H7

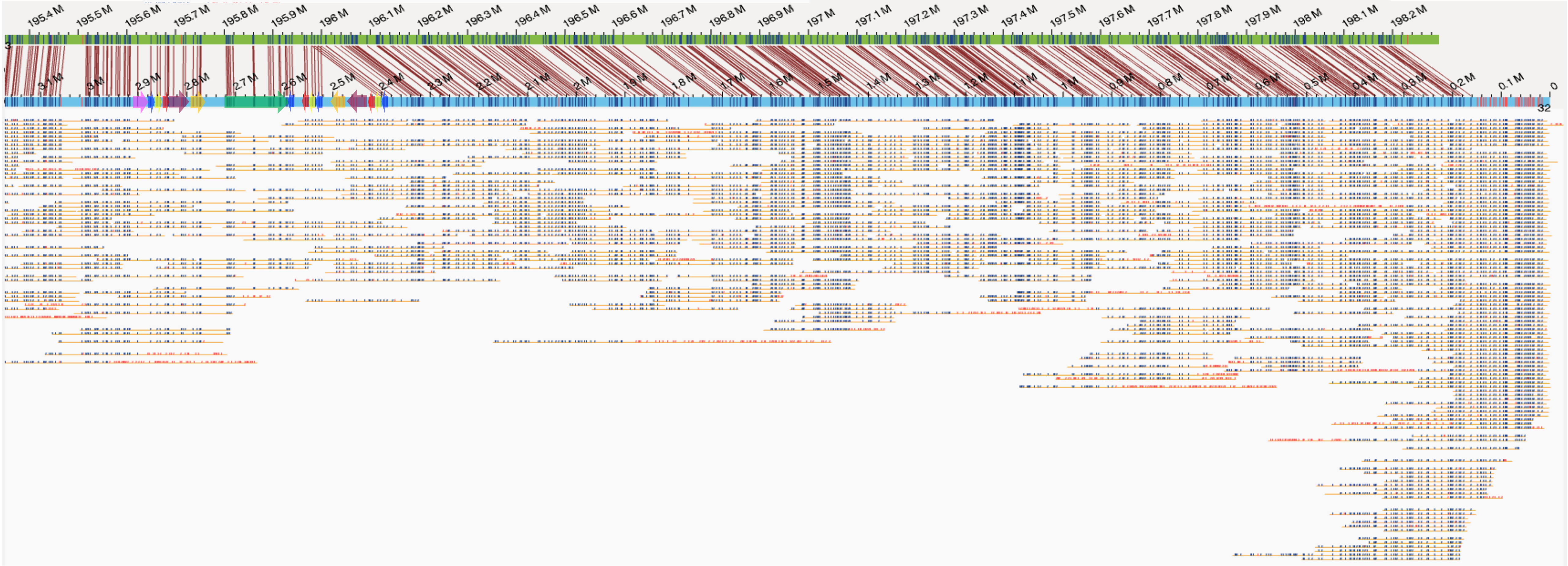

[Go to the first page](#)

Family 9 - Father

H1

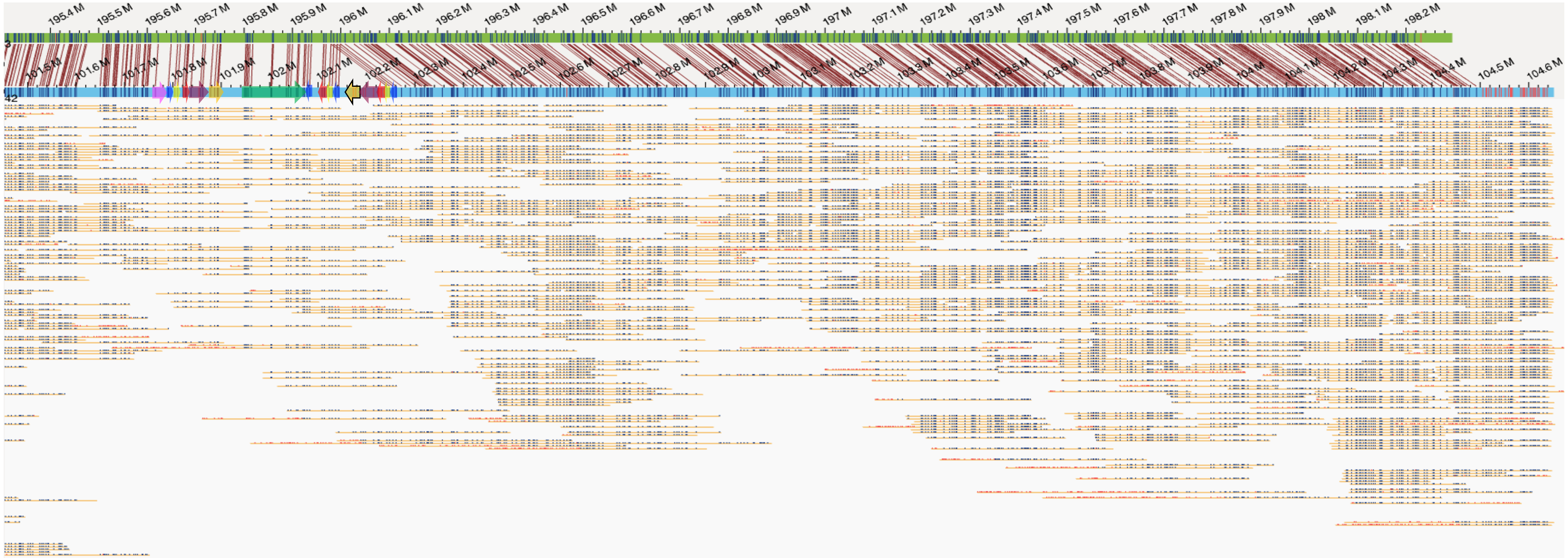

H5

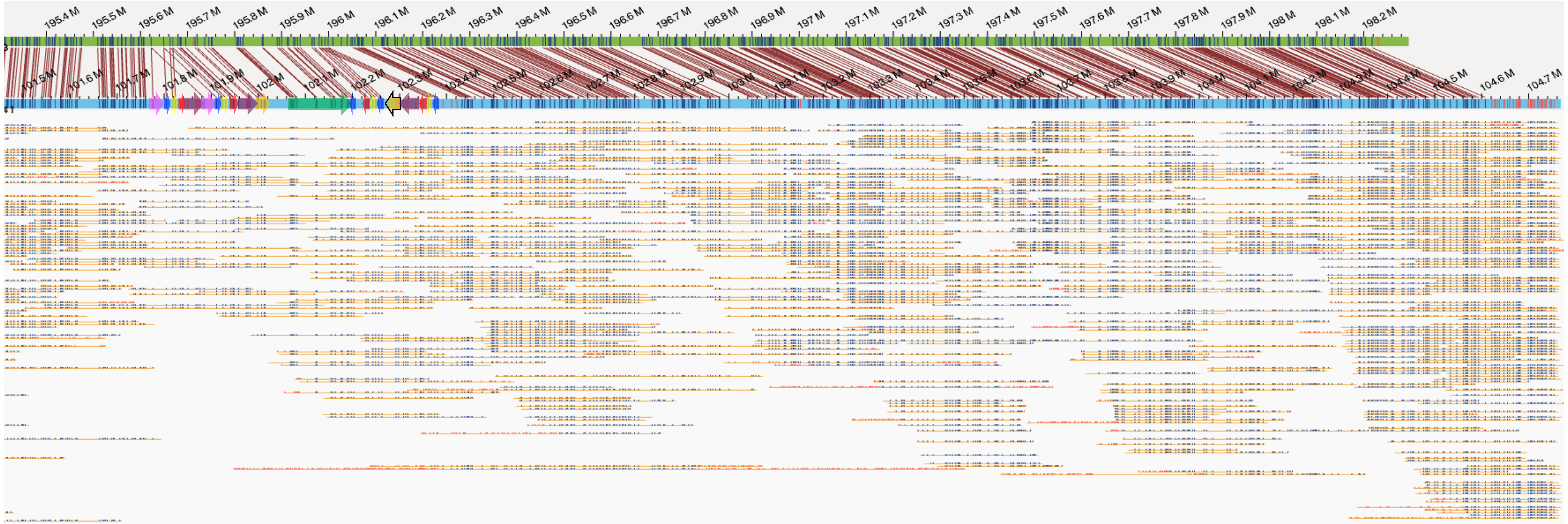

Go to the first page

Family 9 - Mother

H7

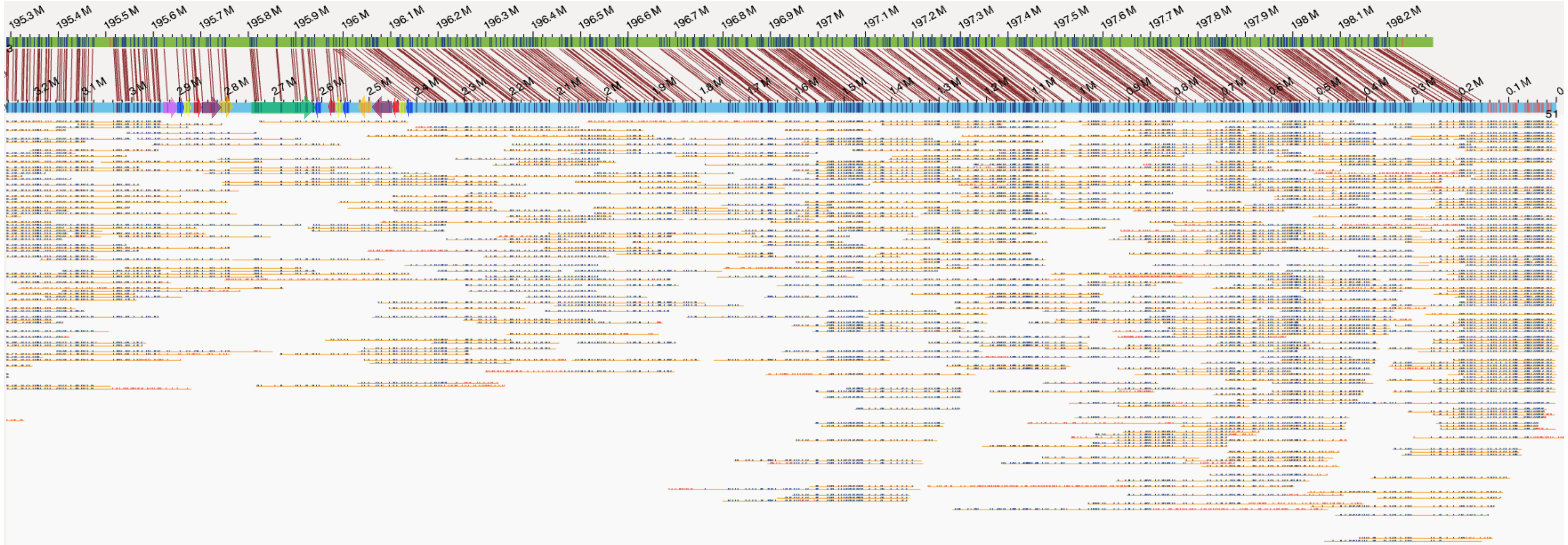

H4

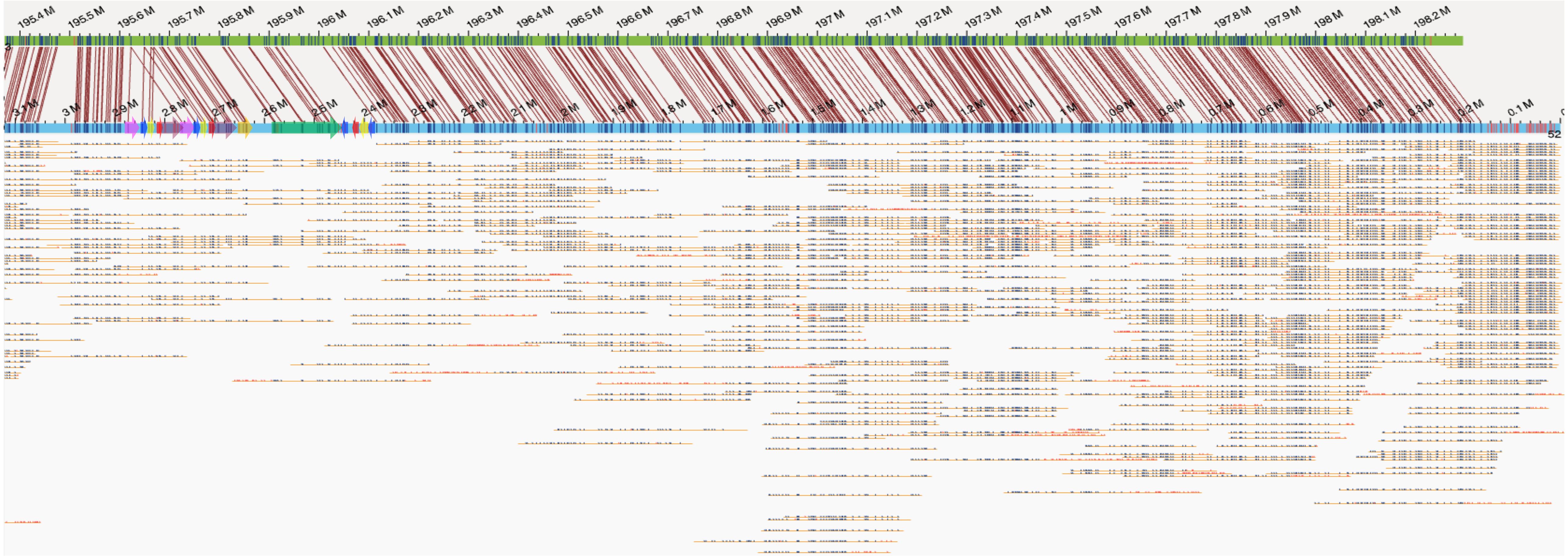

Go to the first page

Family 10 - Trio

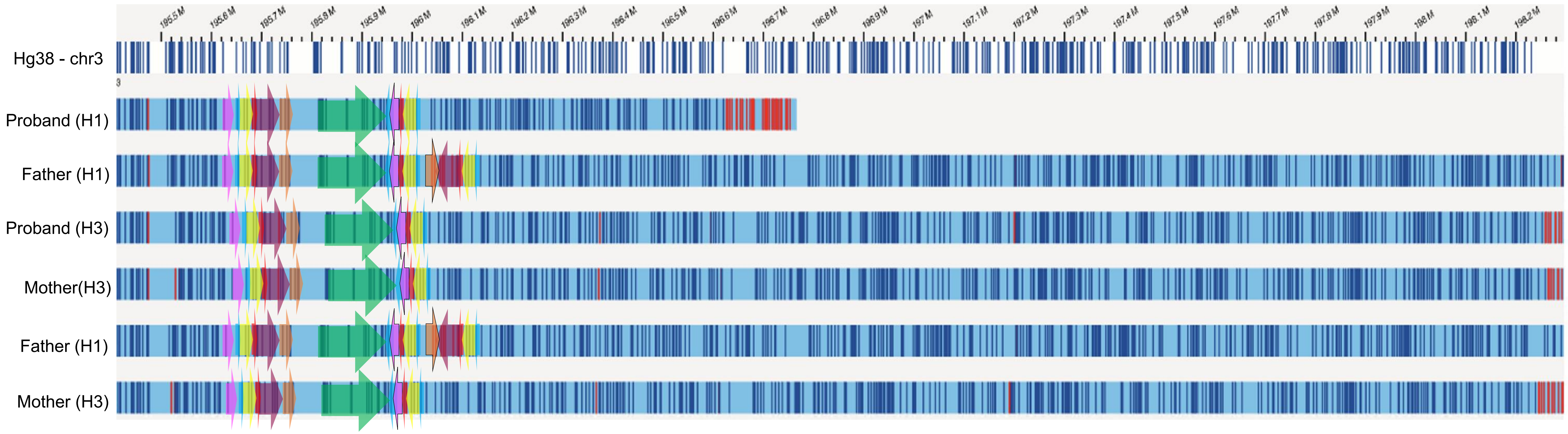

Go to the first page

Family 10 – Proband

H3

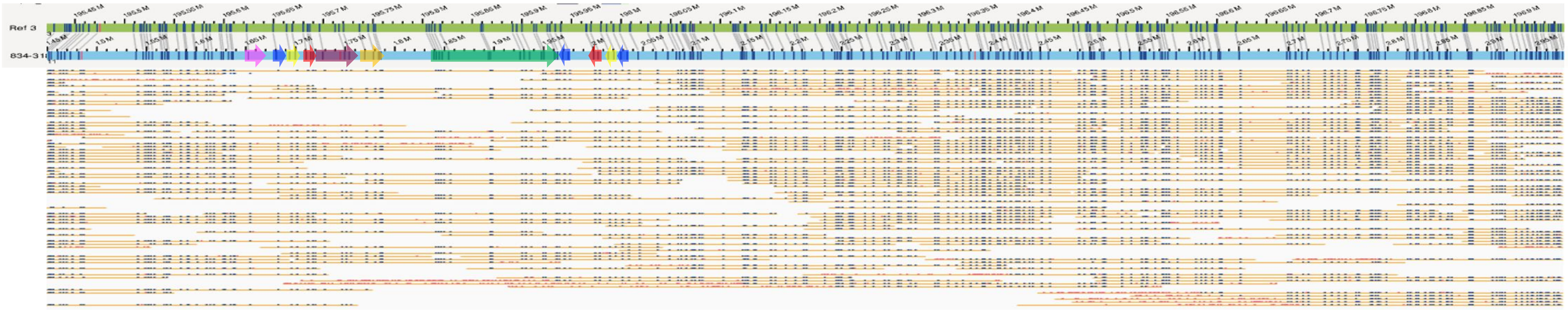

H1

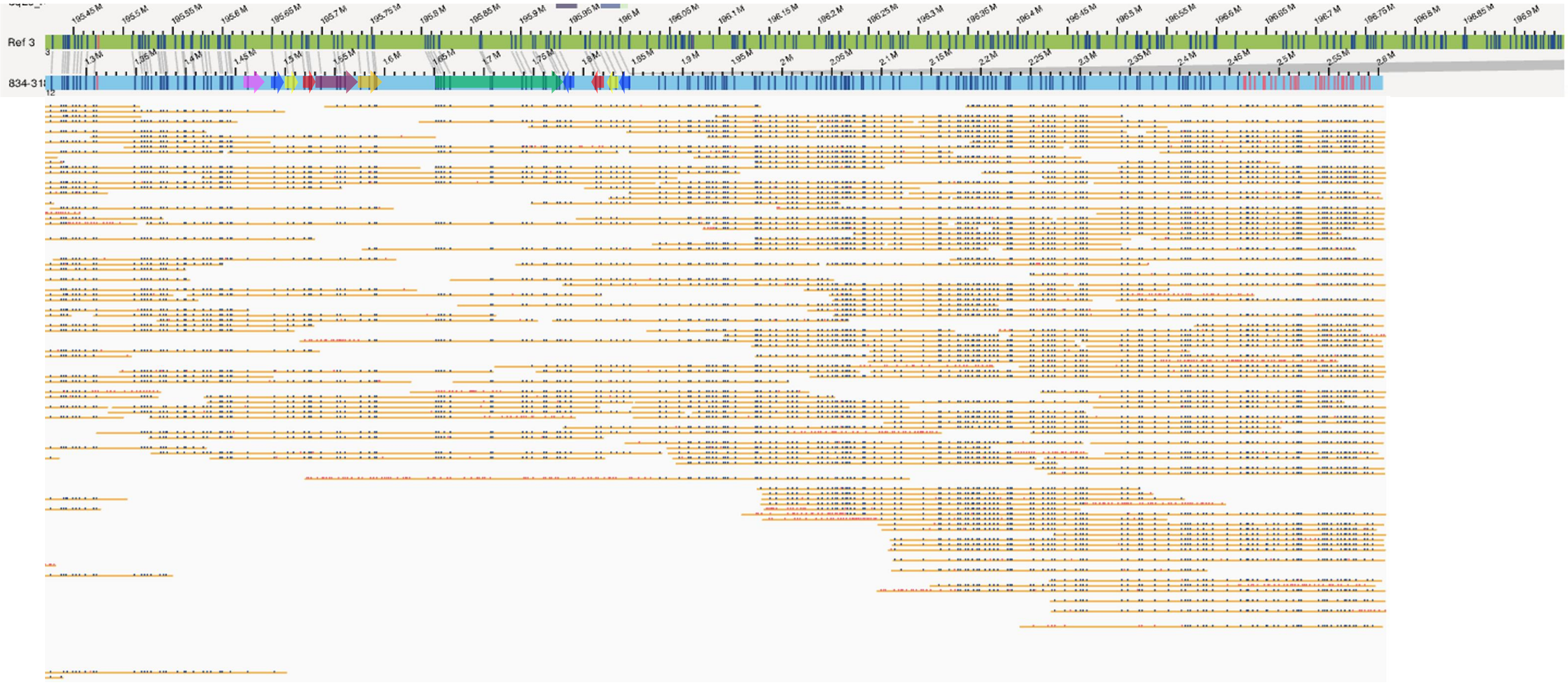

[Go to the first page](#)

Family 10 – Father

H1

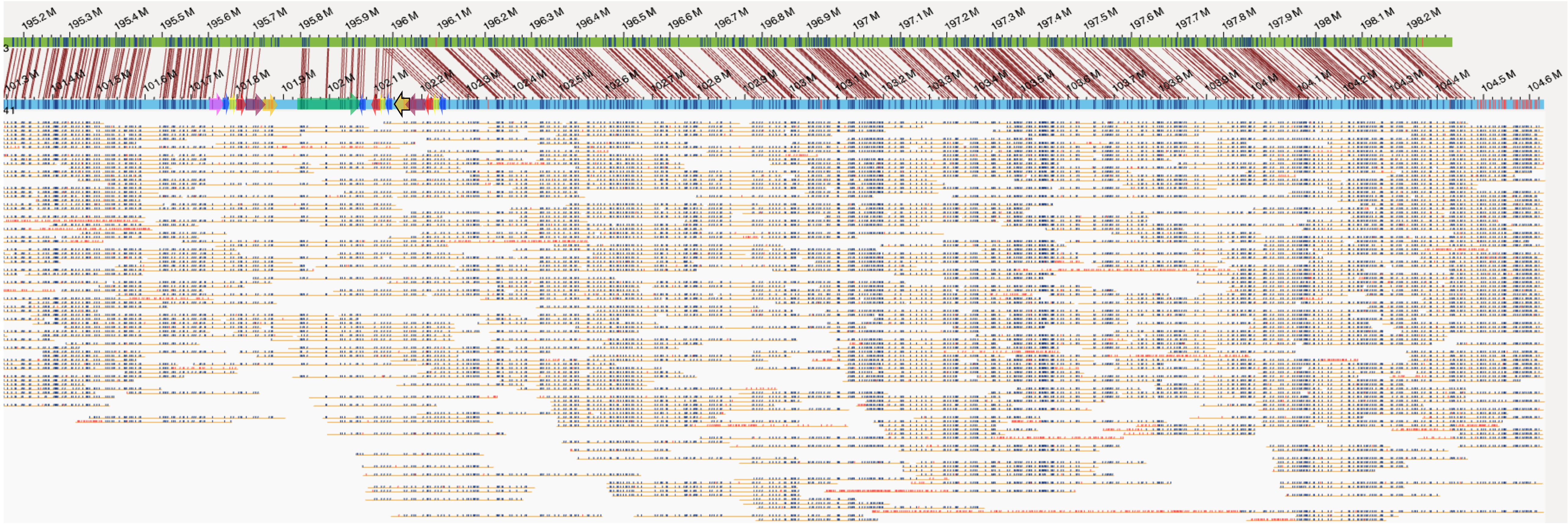

H1

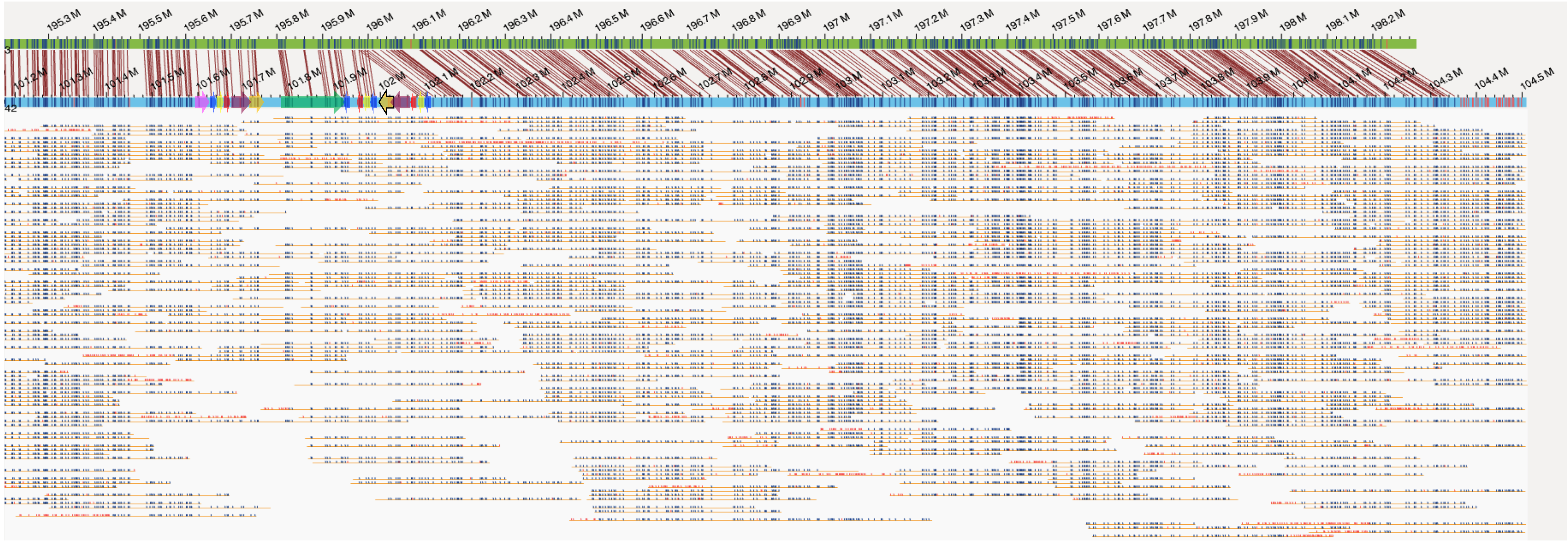

Go to the first page

Family 10 - Mother

H3

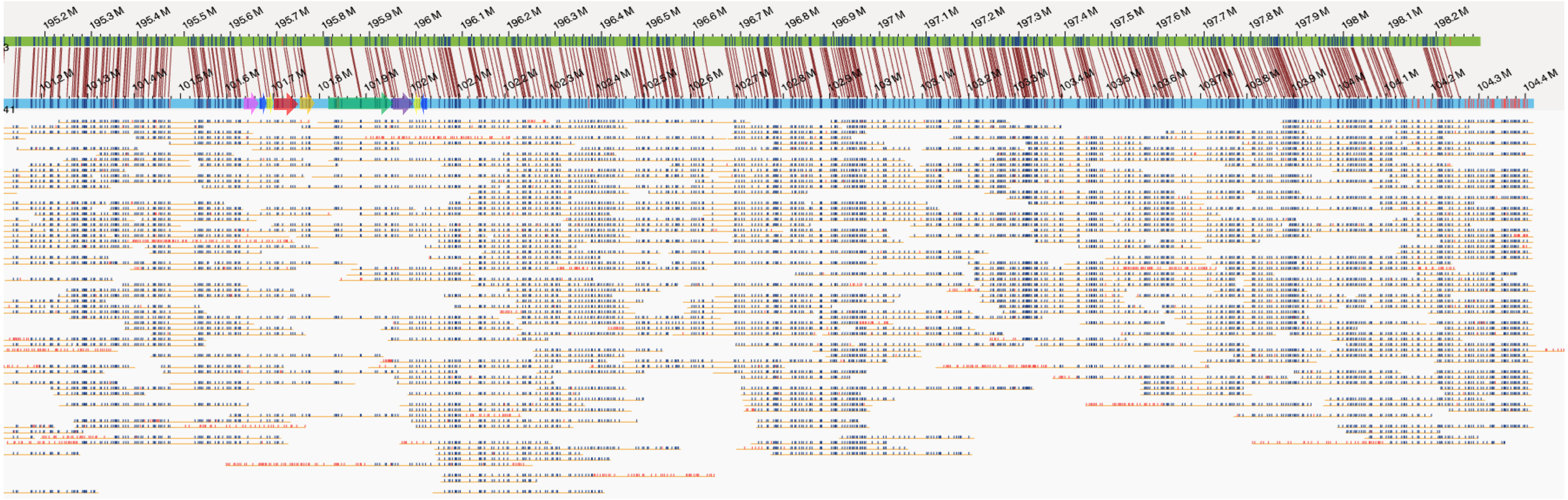

H3

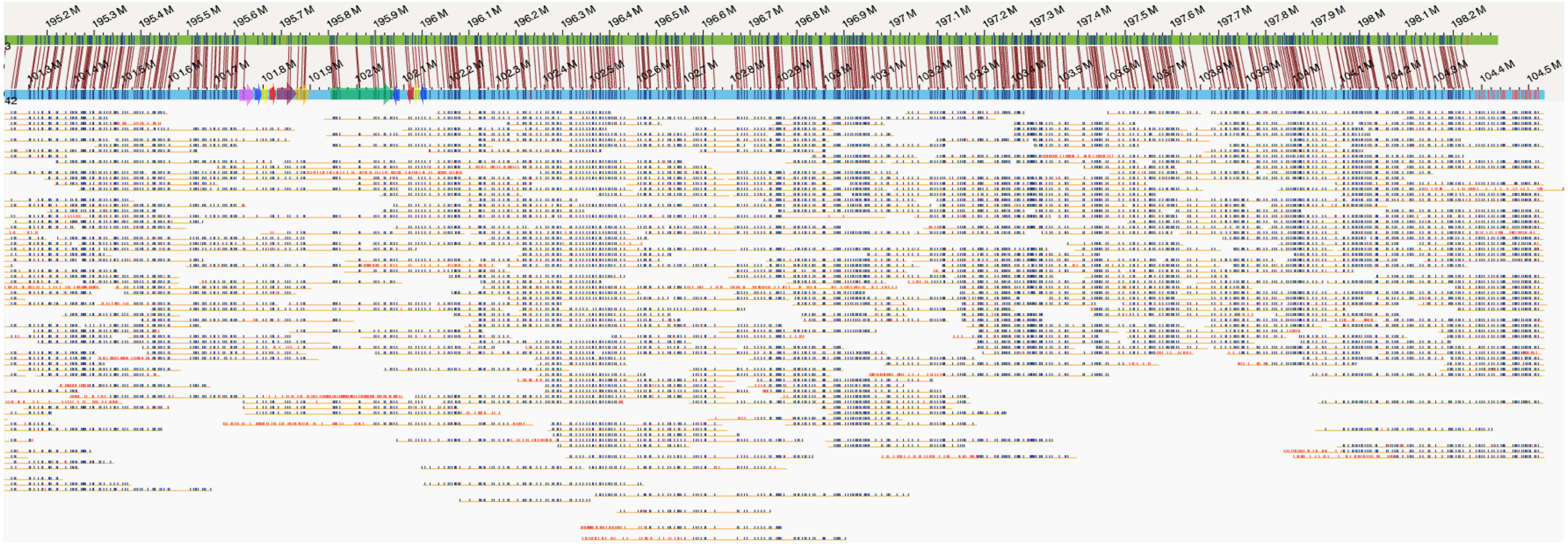

Go to the first page

Family 11 - Trio

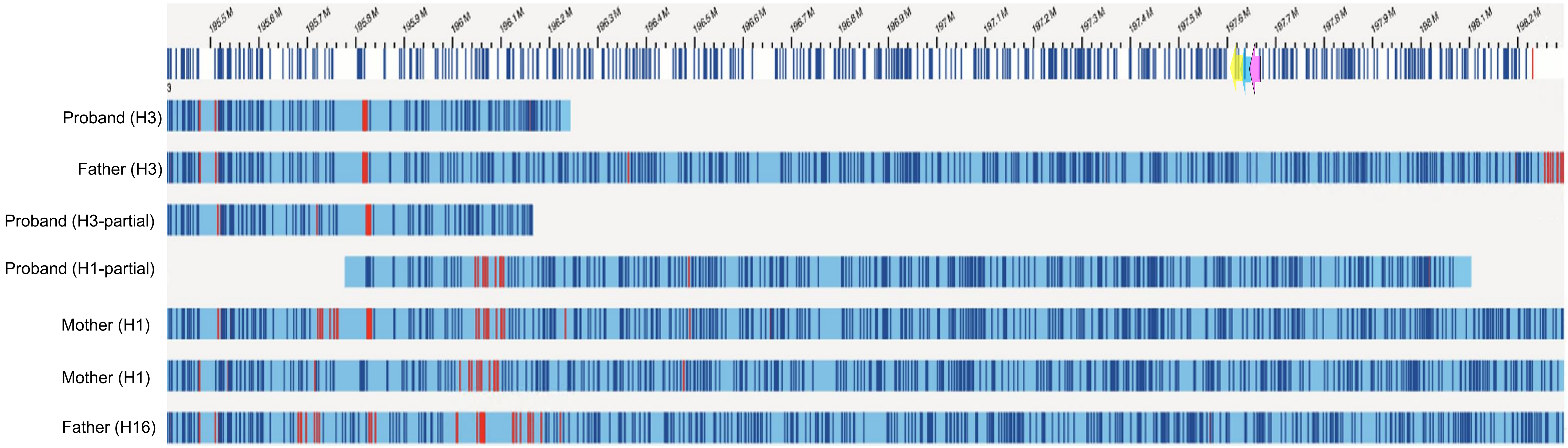

Go to the first page

Family 11 - Proband

H3

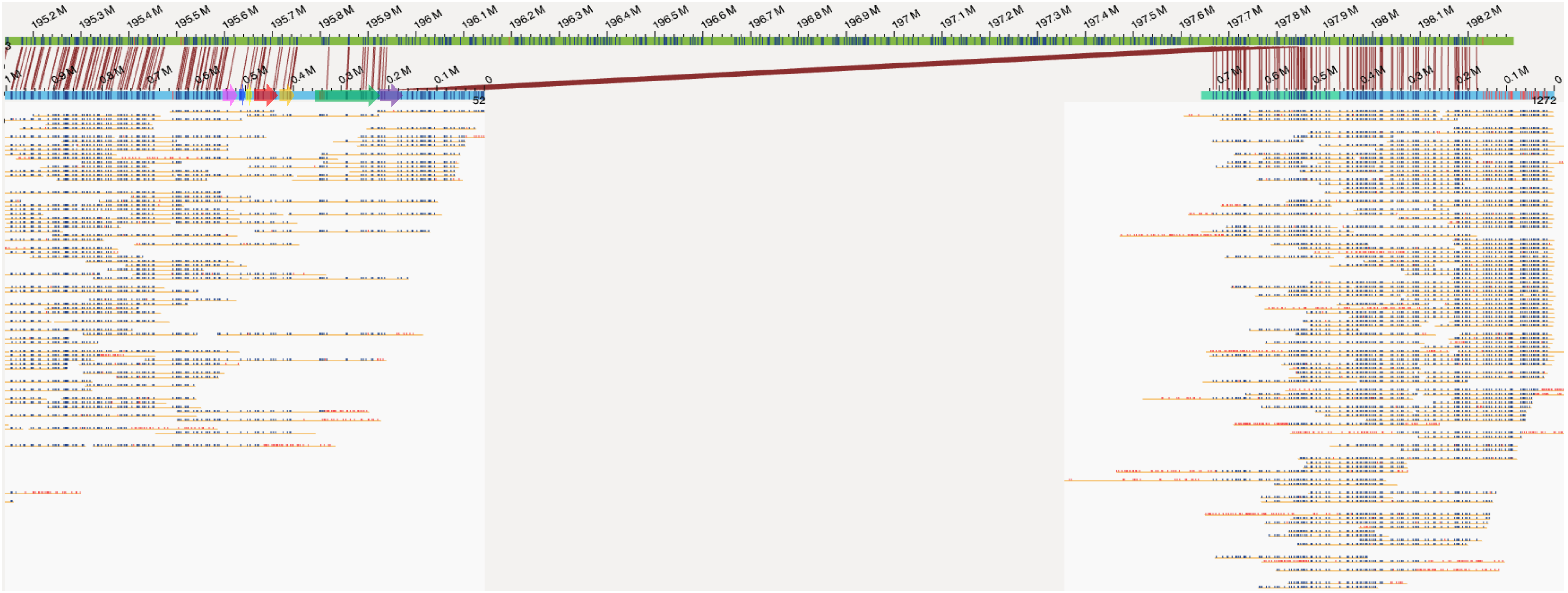

H1

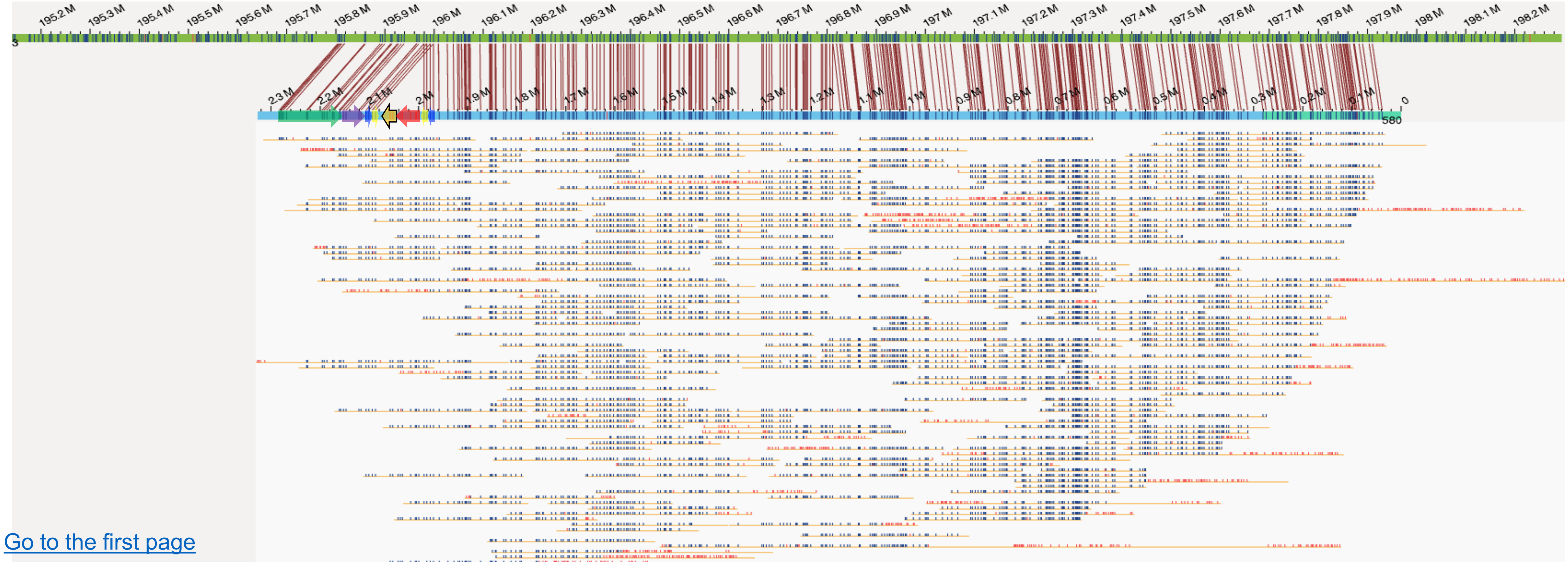

Go to the first page

Family 11 - Father

H16

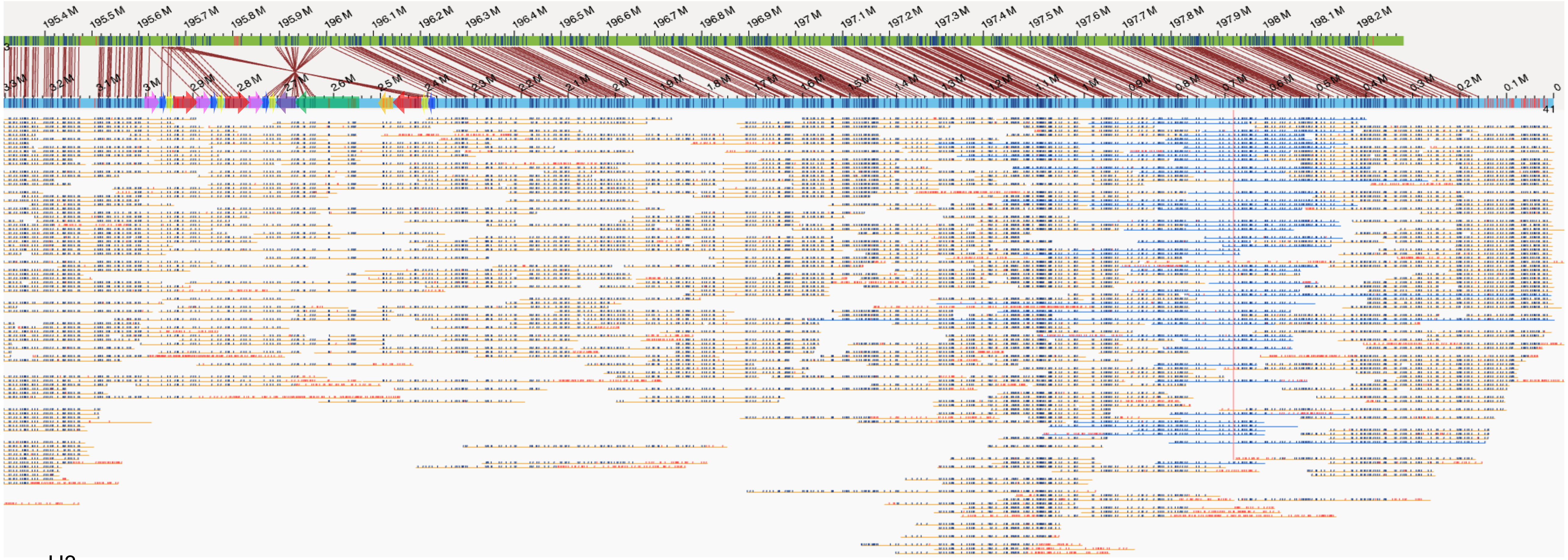

H3

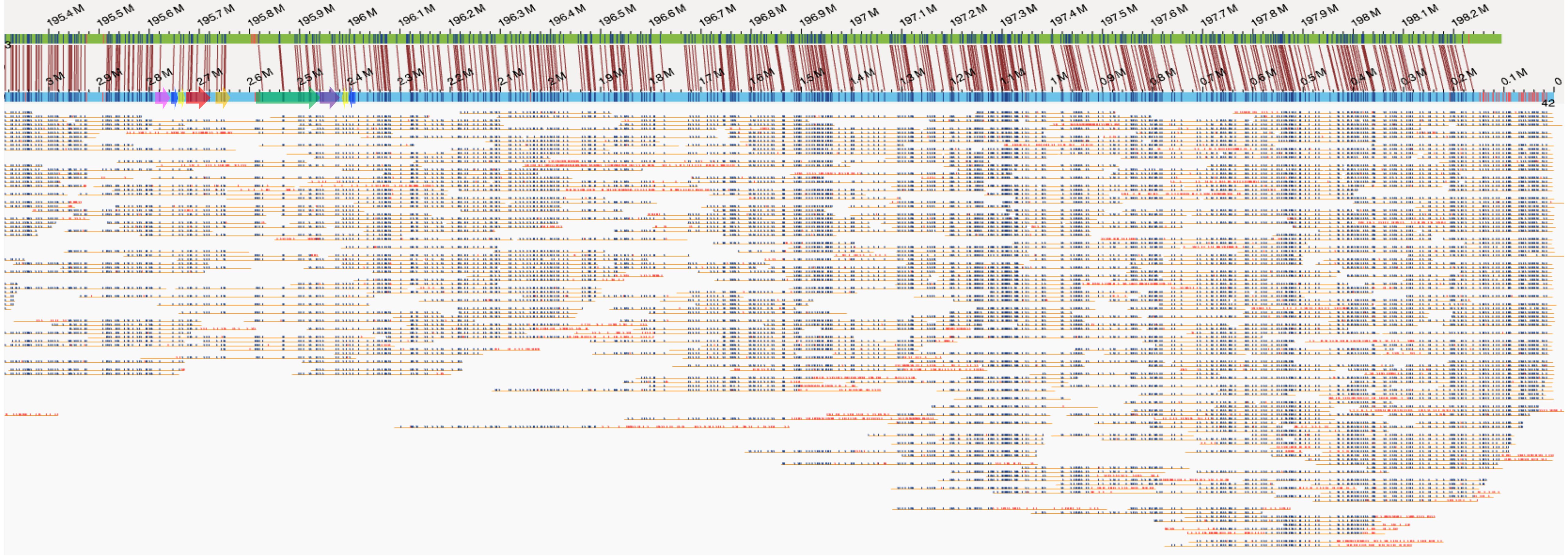

Go to the first page

Family 11 - Mother

H1

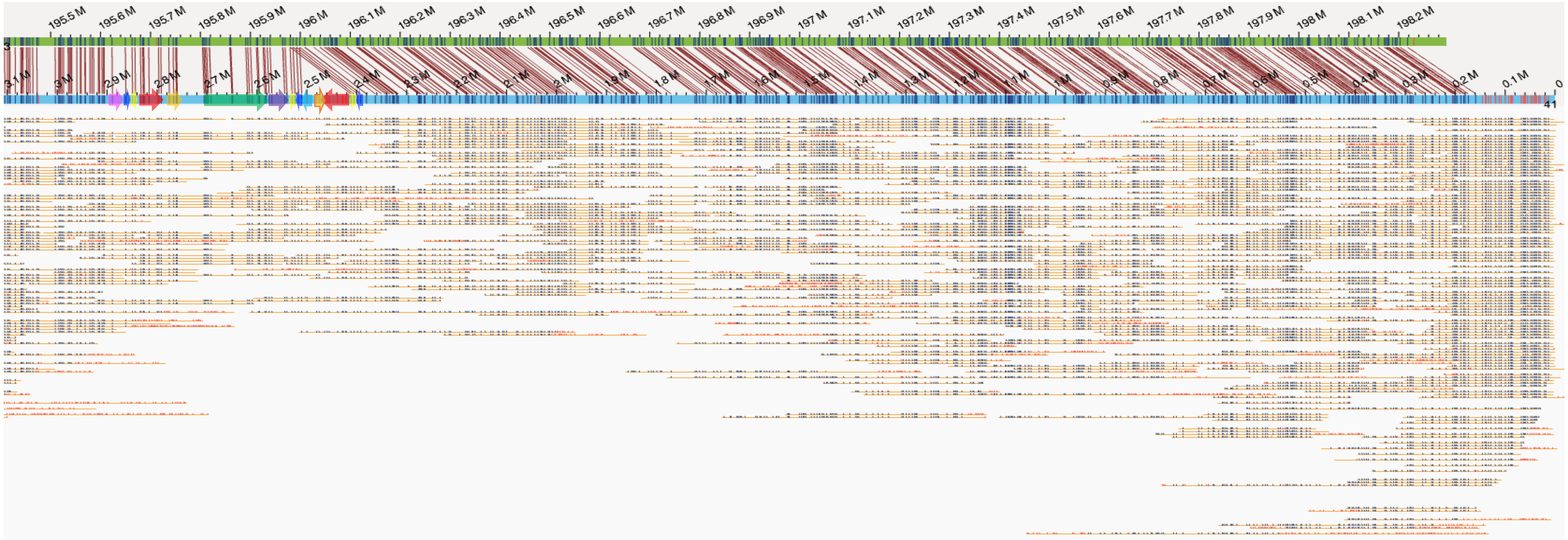

H1

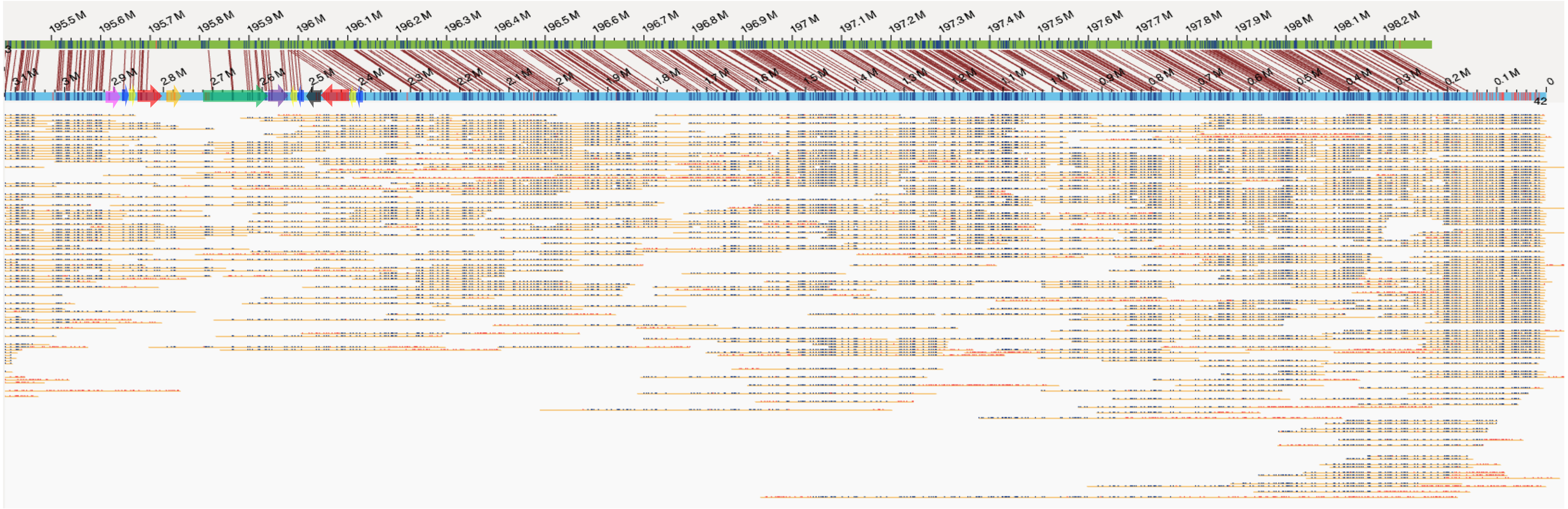

Go to the first page

Family 12 – Proband only

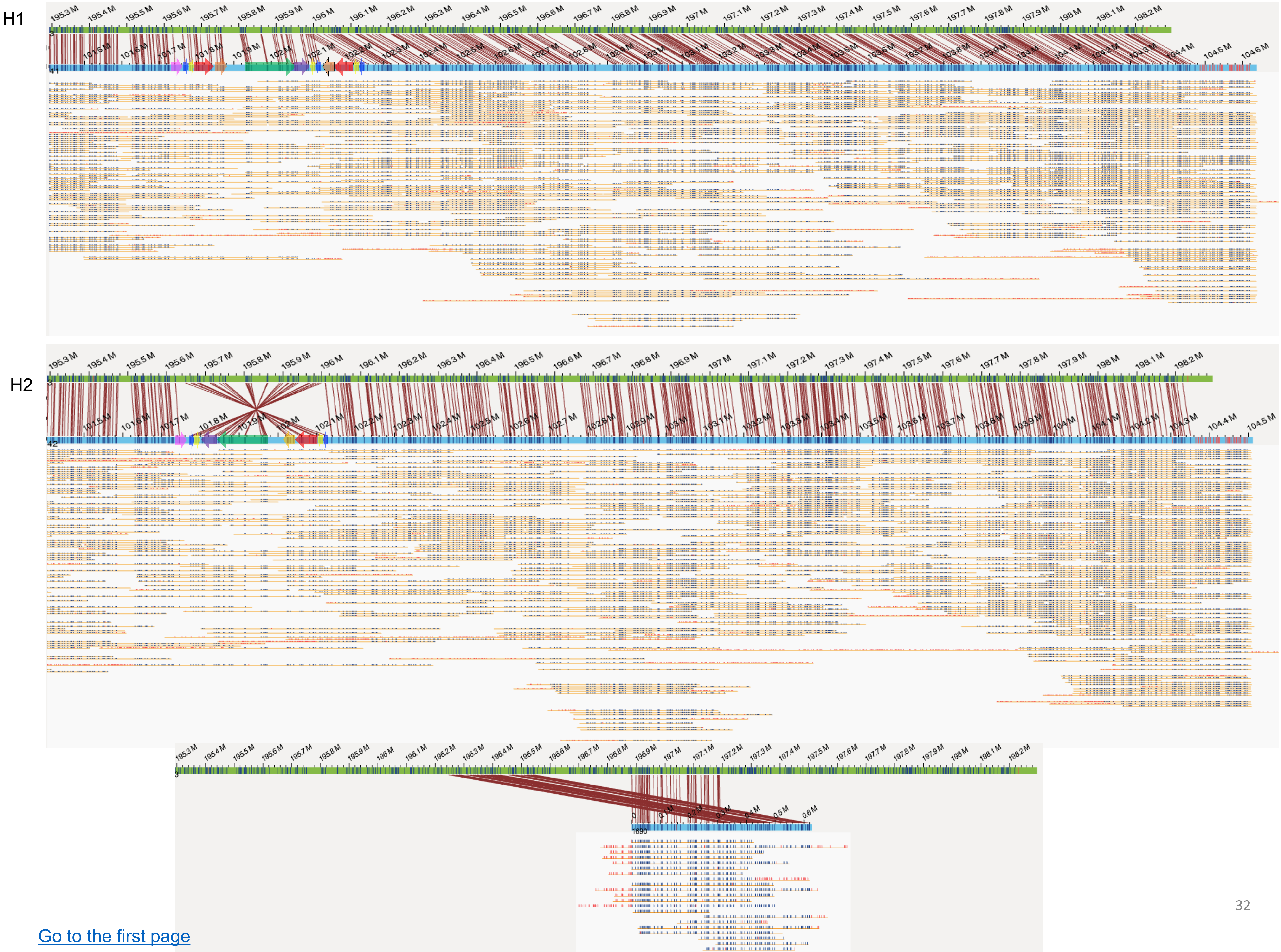

Go to the first page

Family 13 - Trio

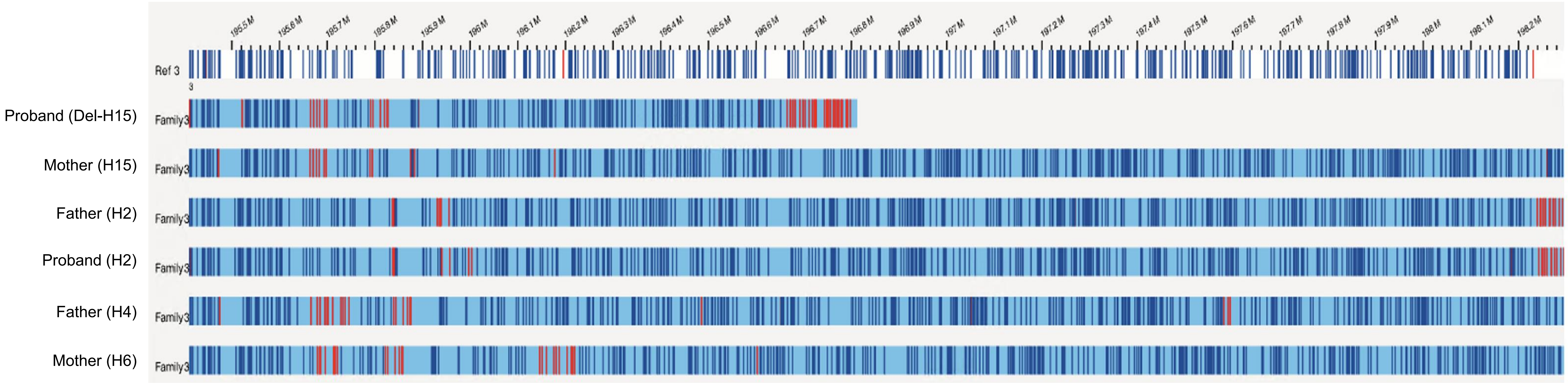

Go to the first page

Family 13 – Proband

H15

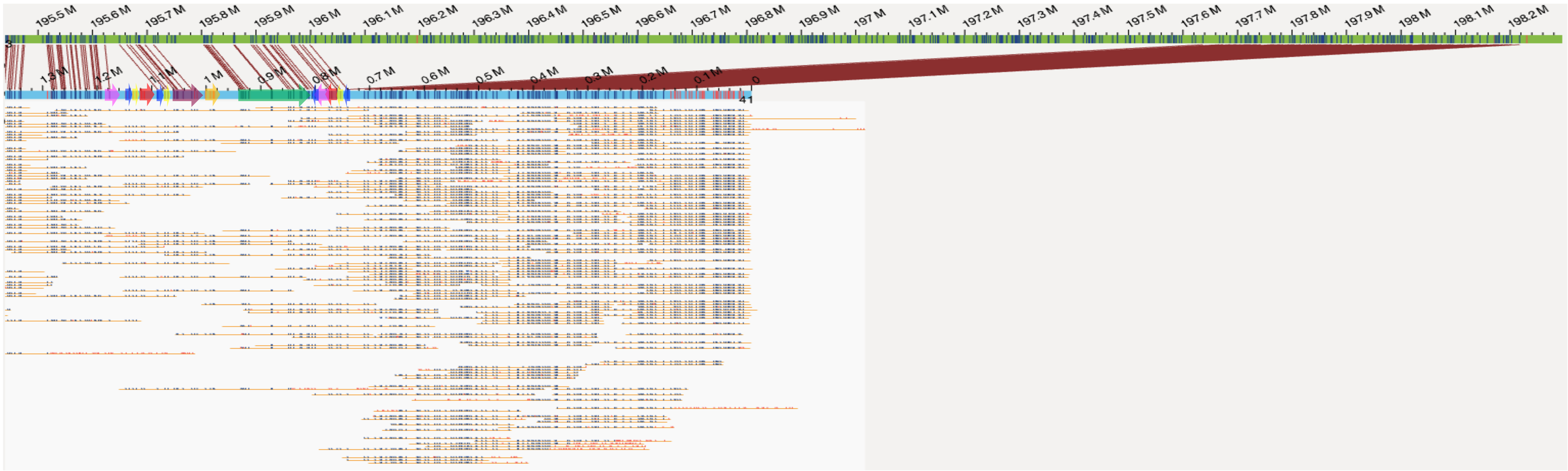

H2

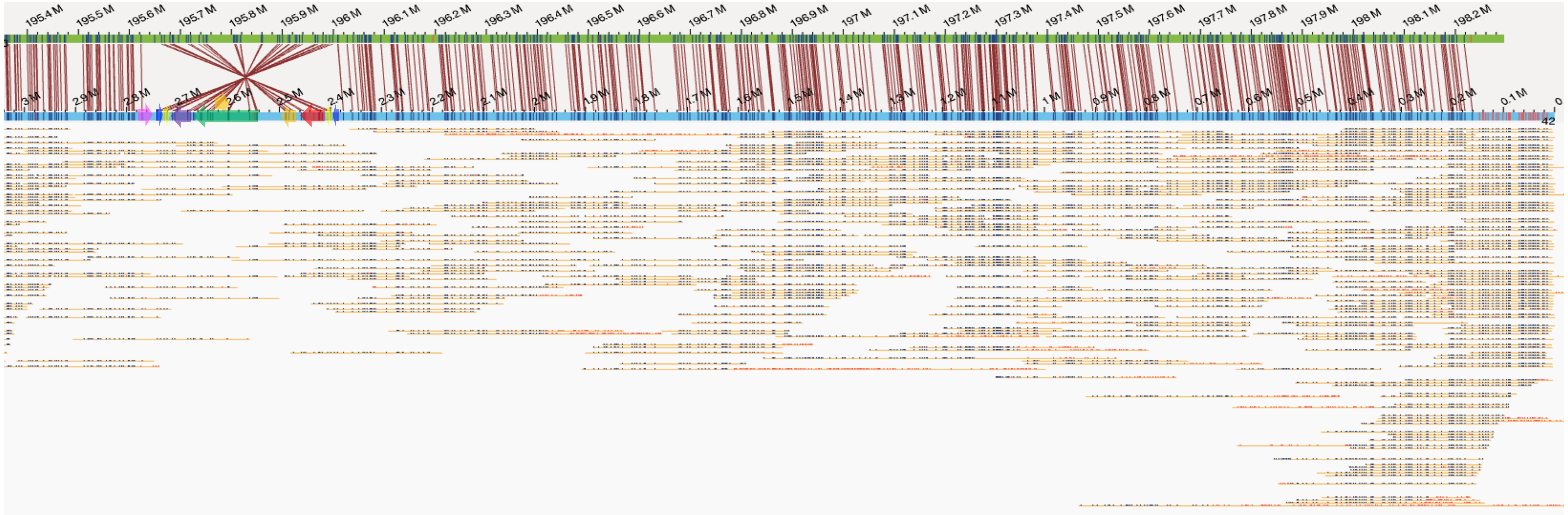

Go to the first page

Family 13 - Father

H4

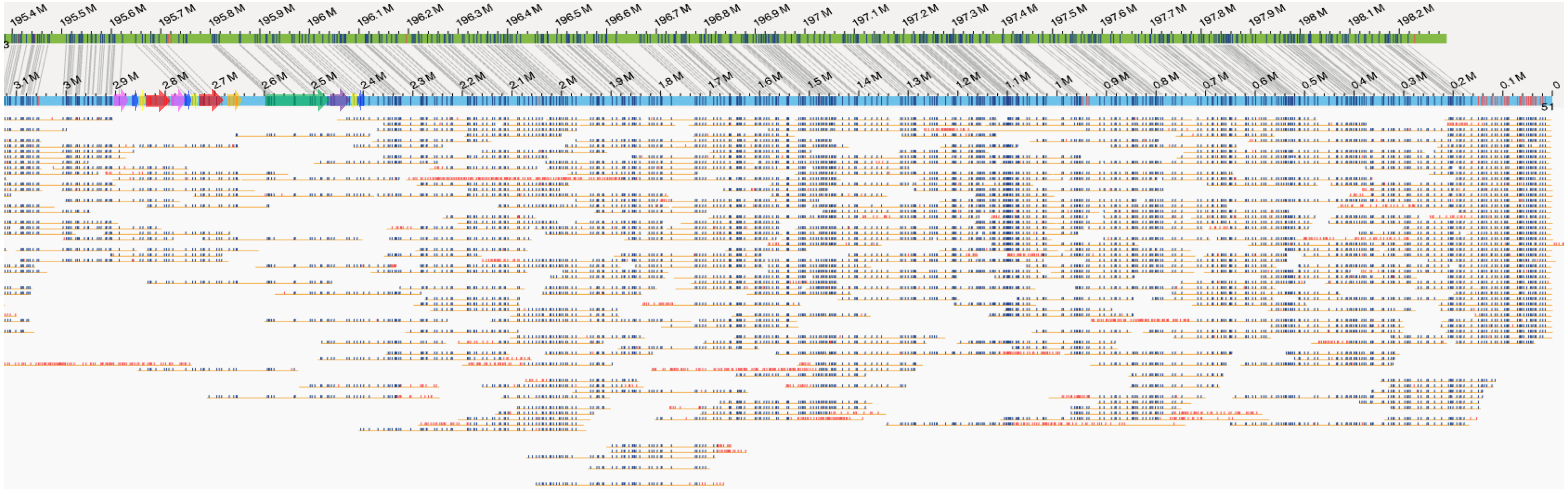

H2

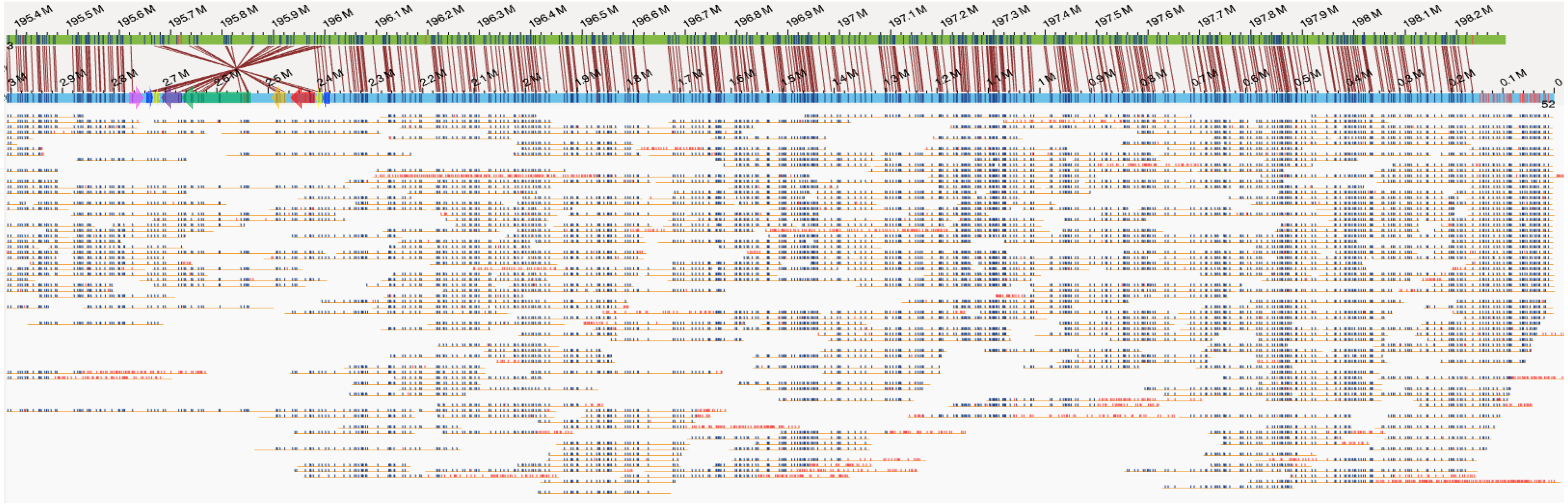

Go to the first page

Family 13 - Mother

H6

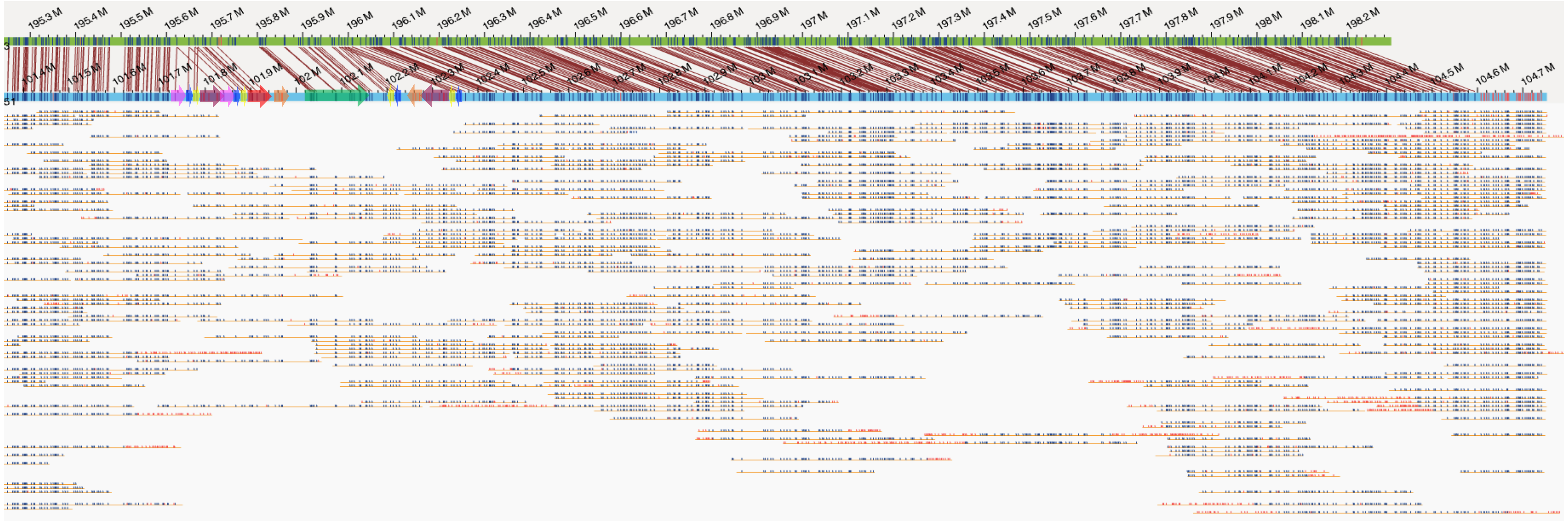

H15

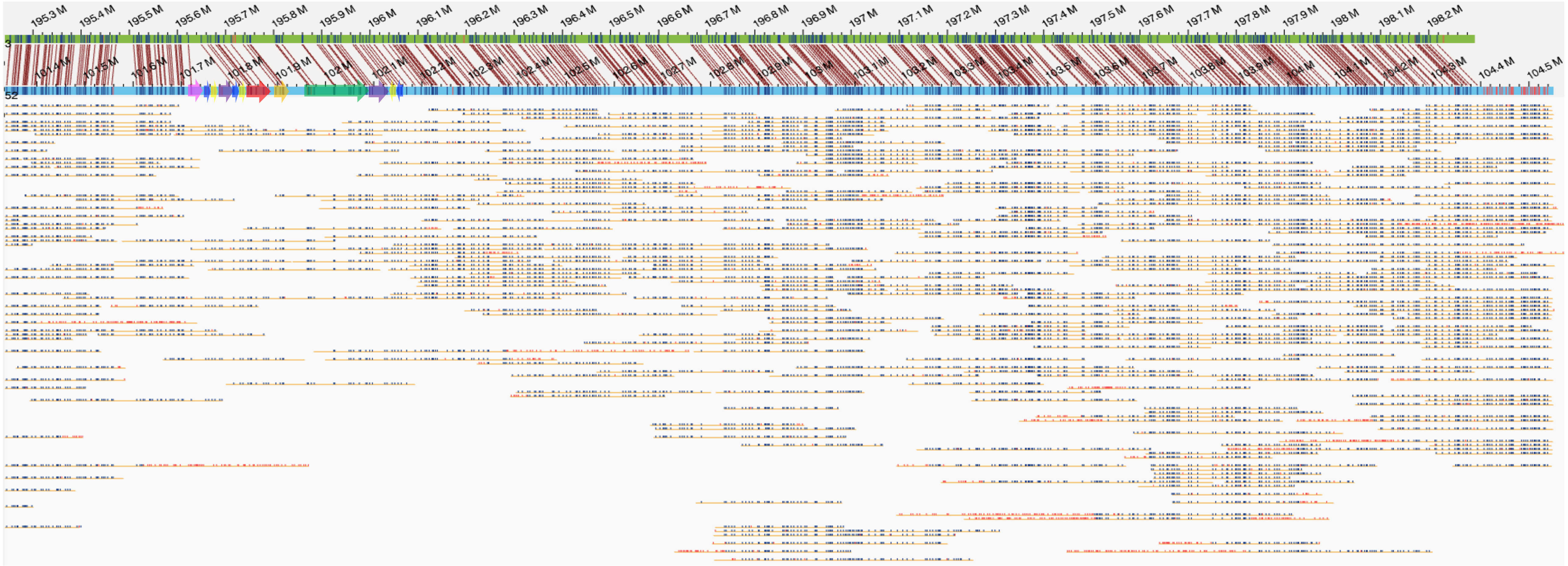

Go to the first page

Family 14 – Trio + sibling

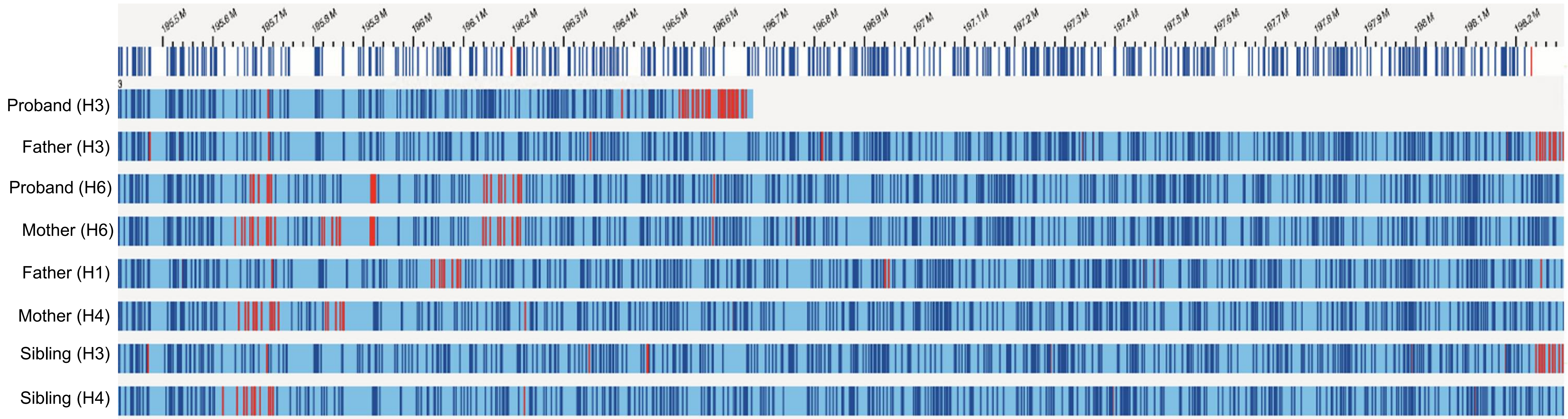

Go to the first page

Family 14 - Proband

H3

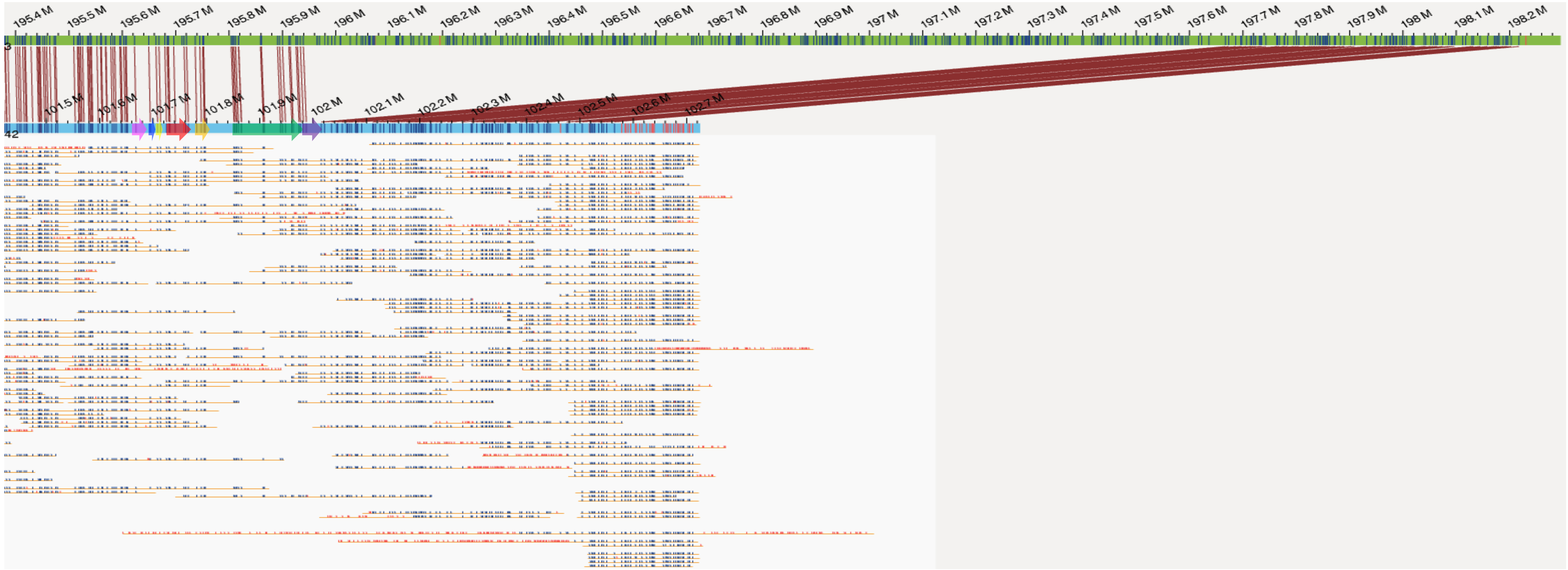

H6

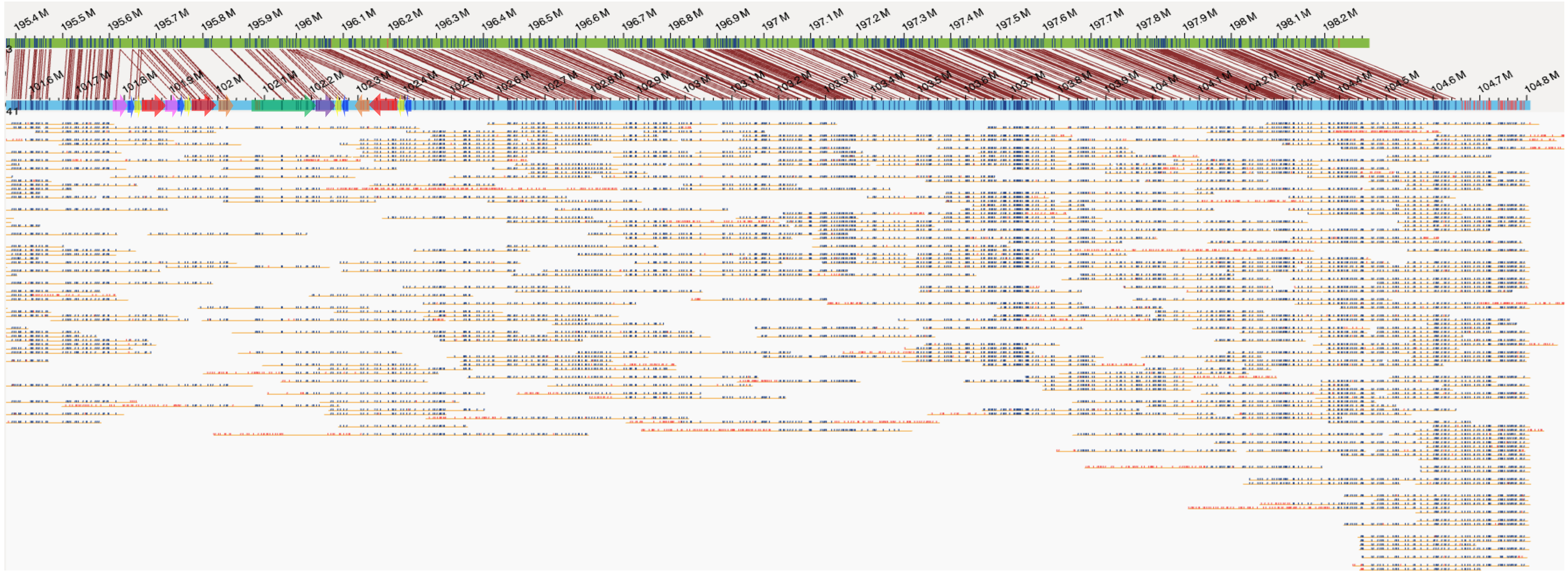

Go to the first page

Family 14 - Sibling

H4

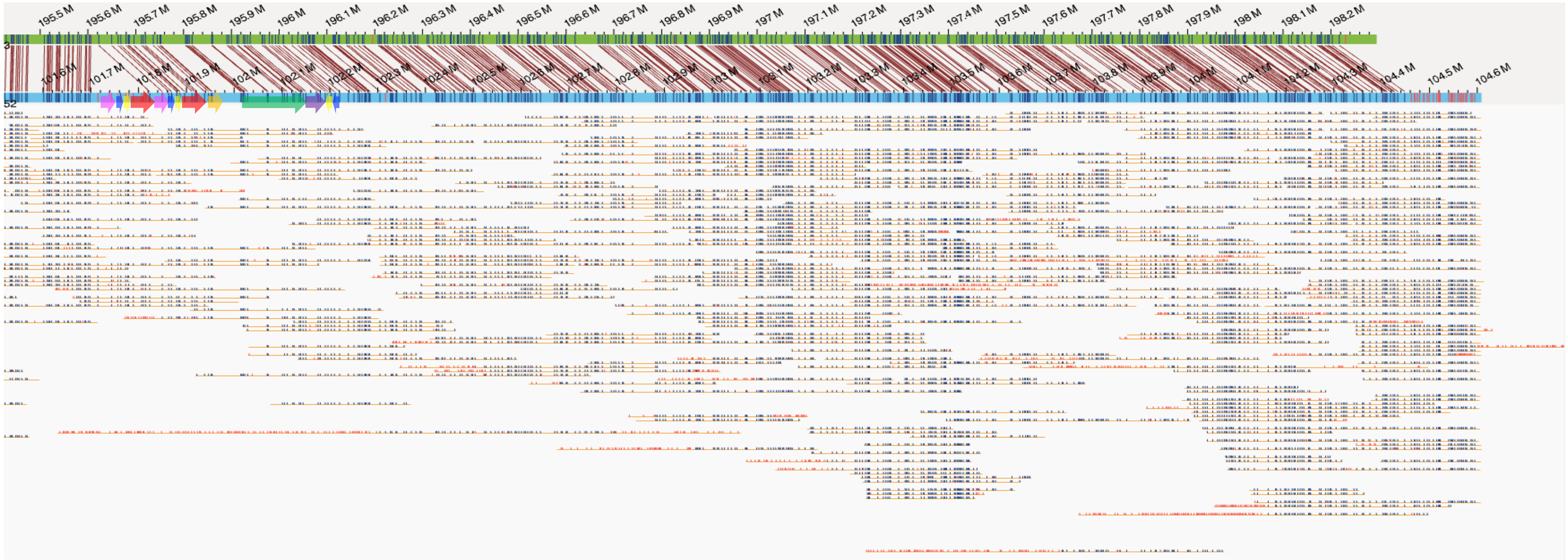

H3

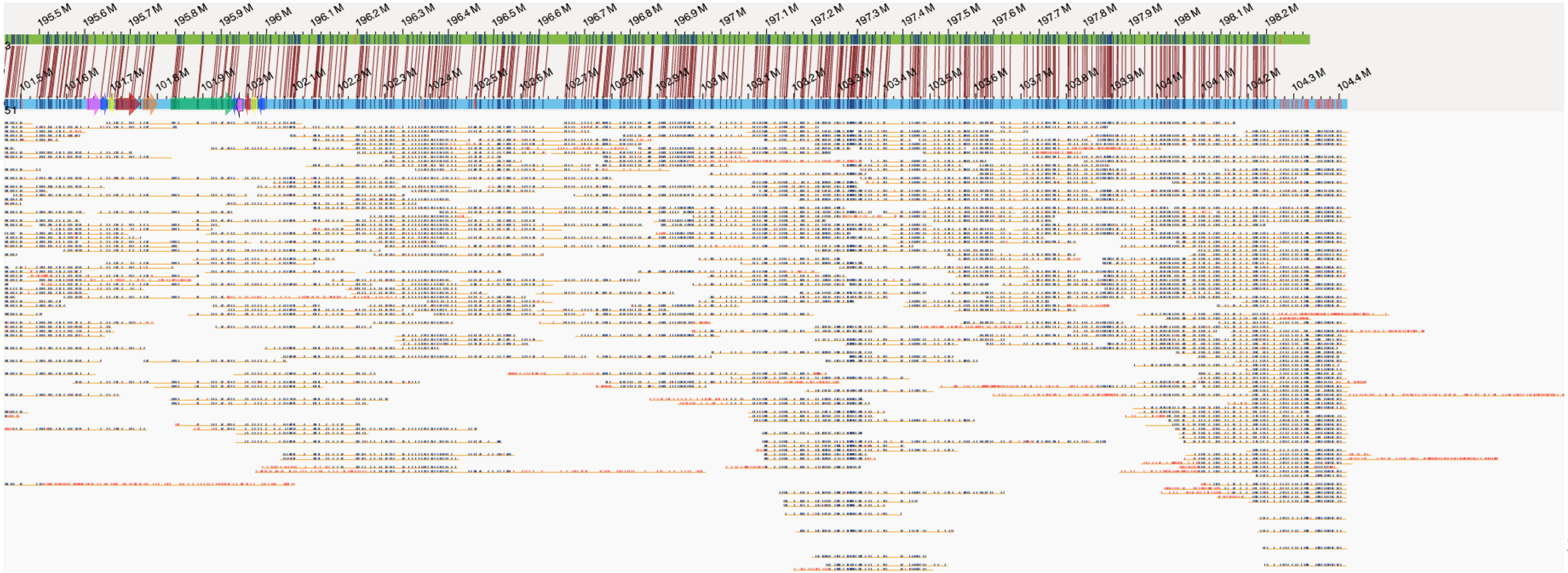

Go to the first page

Family 14 – Father

H3

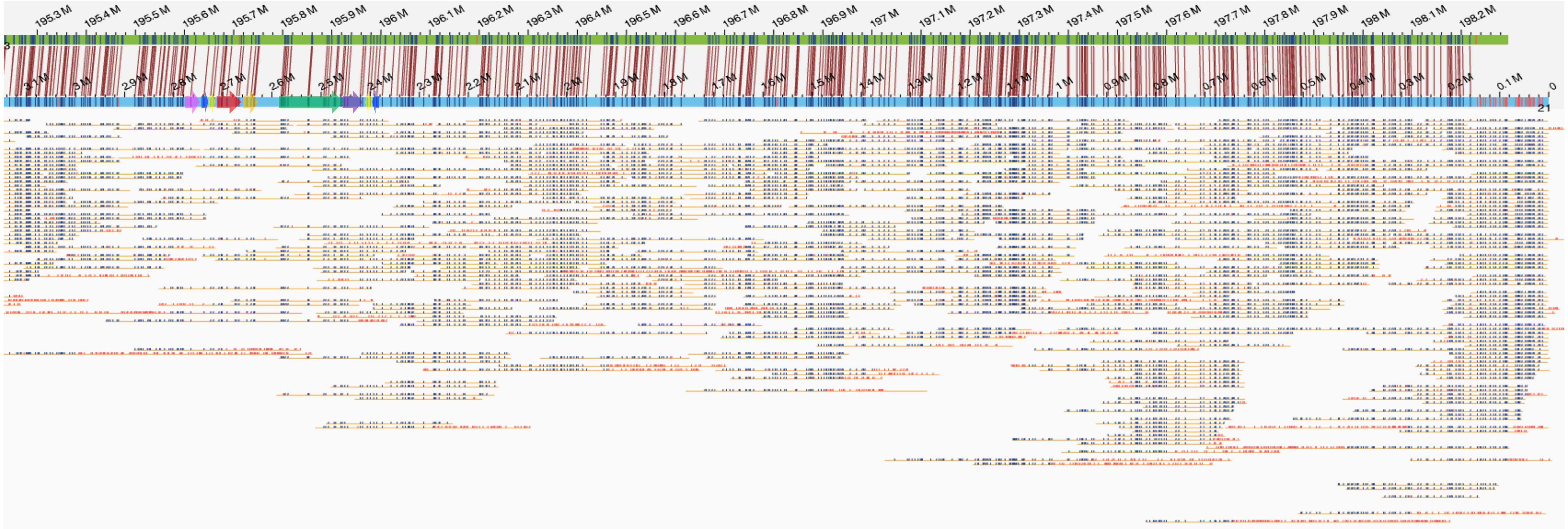

H1

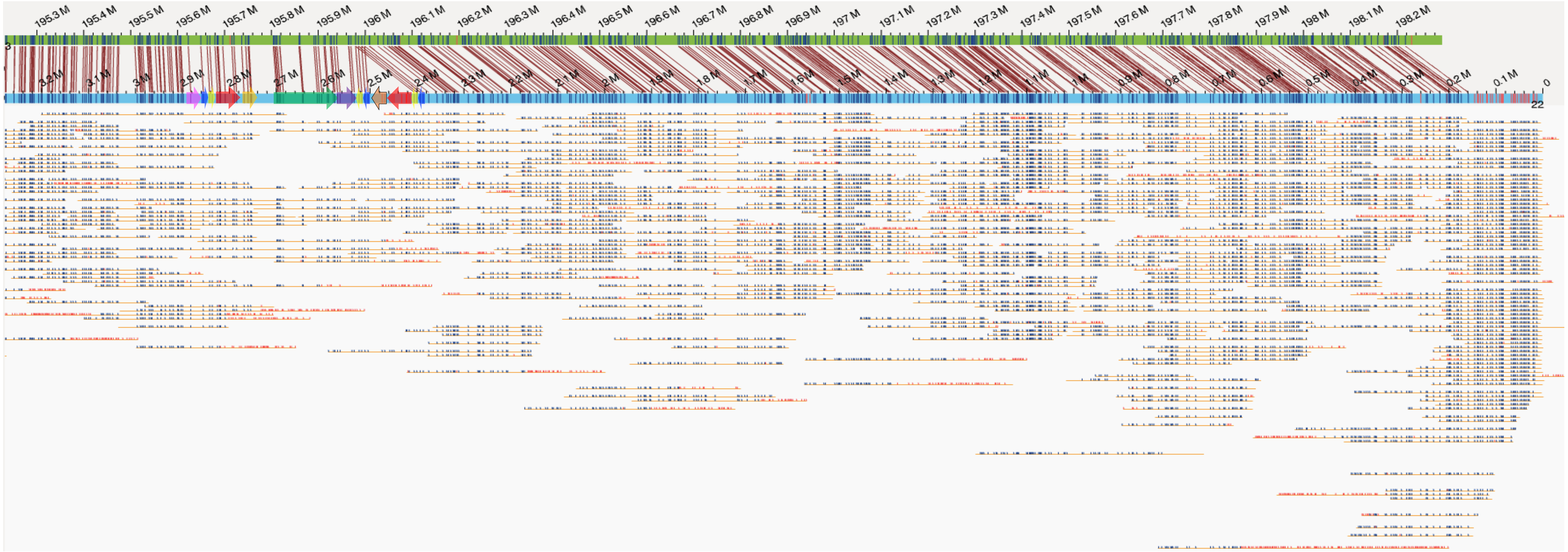

Go to the first page

Family 14 – Mother

H4

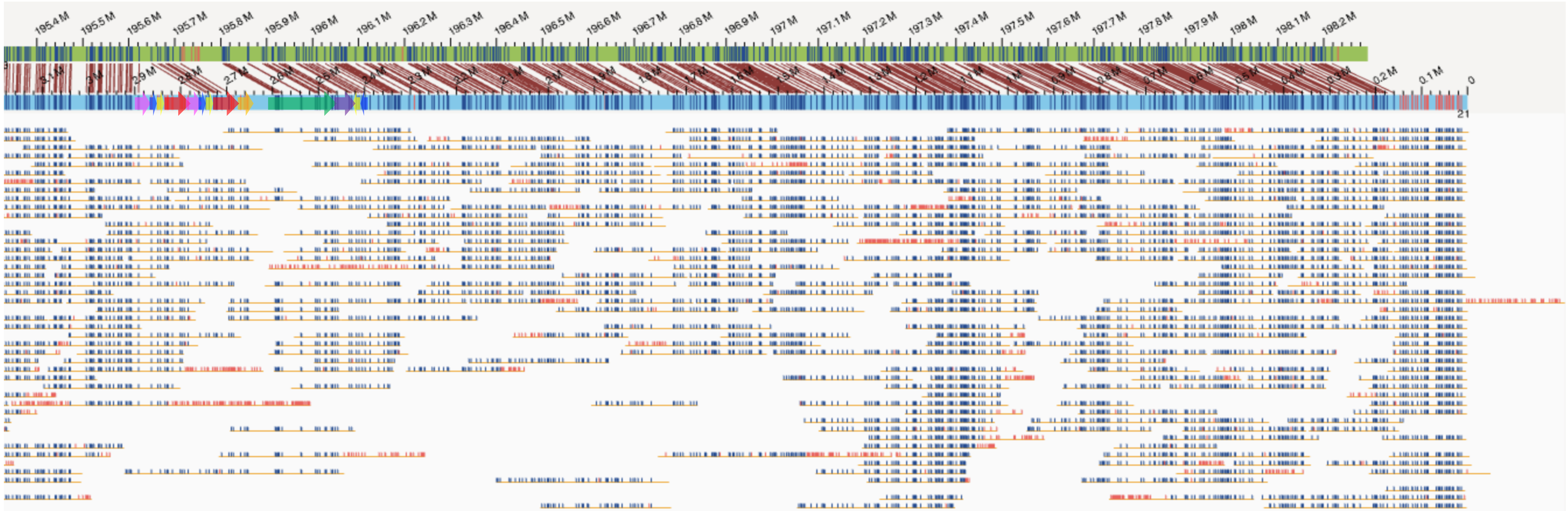

H6

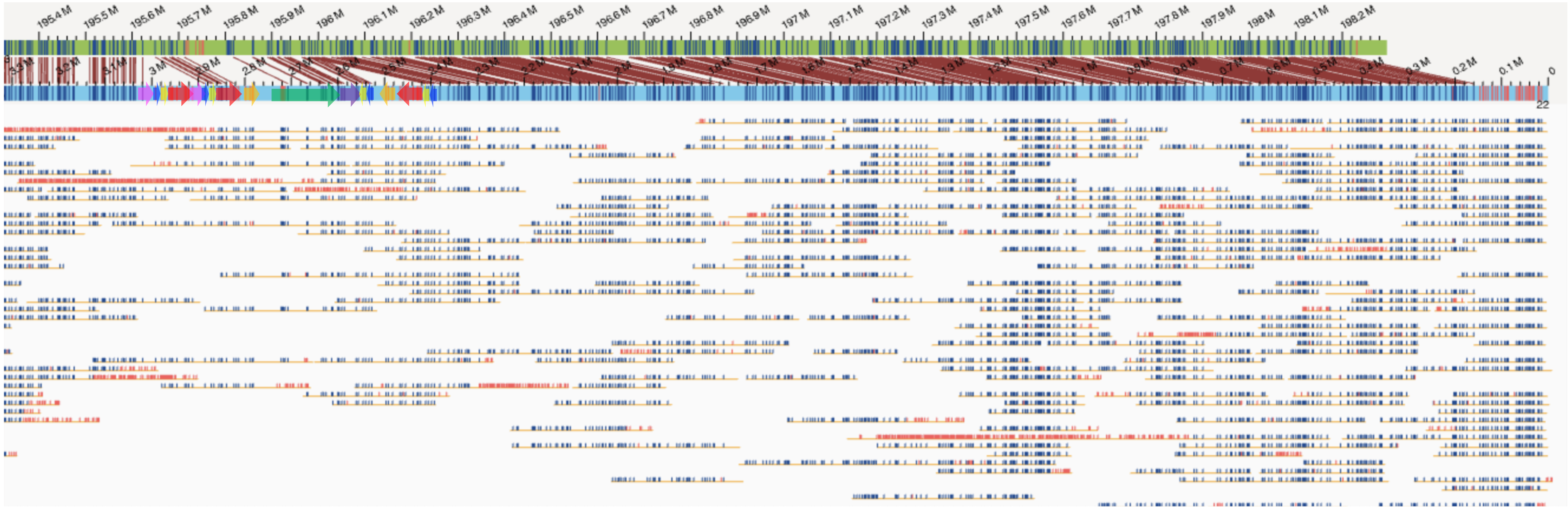

Go to the first page

Family 15 - Trio

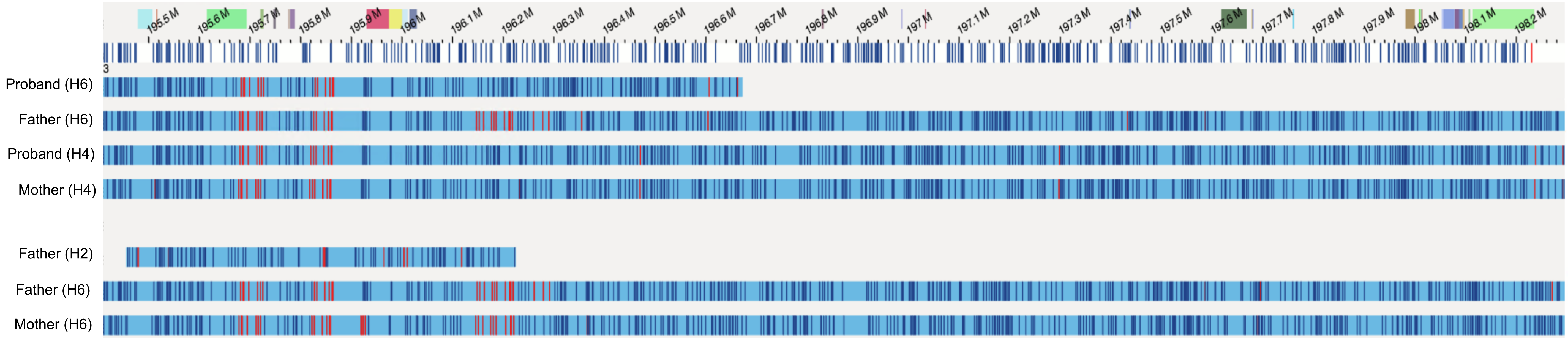

Go to the first page

Family 15 – Proband

H6

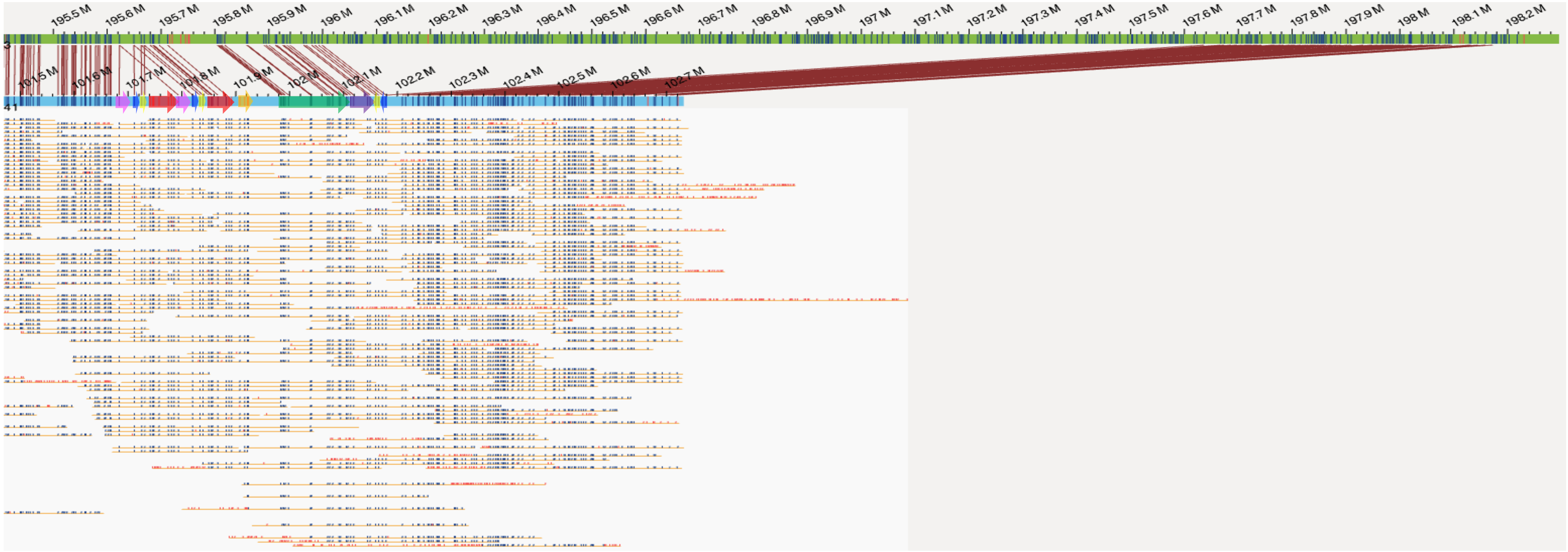

H4

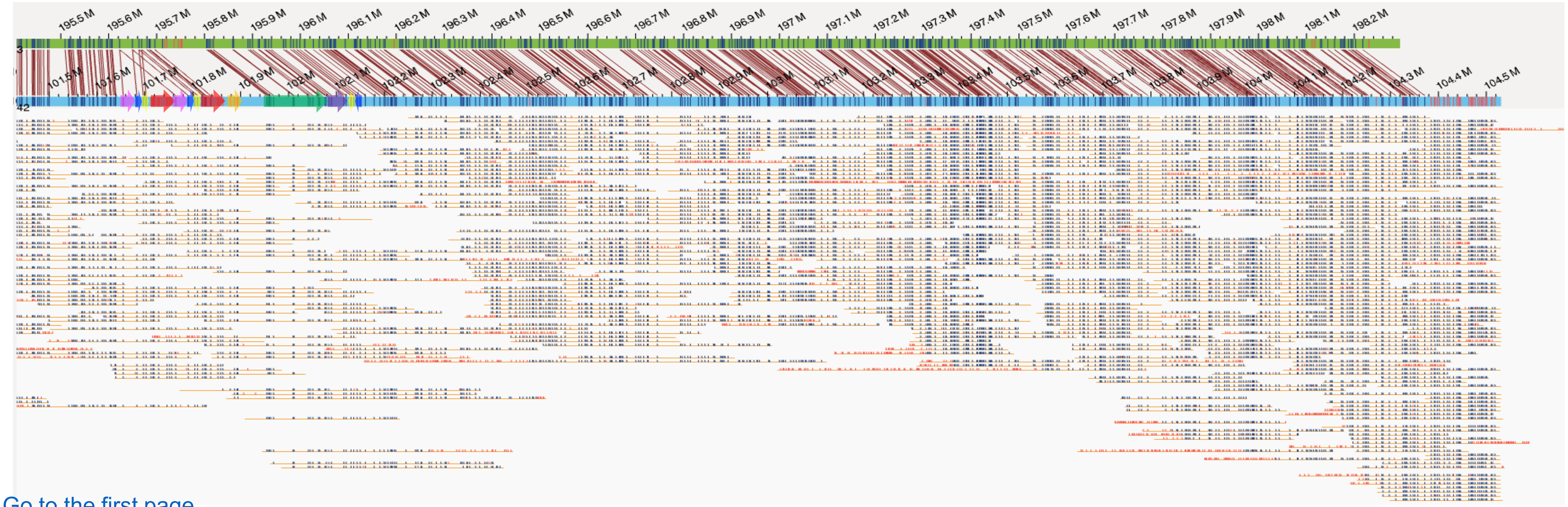

Go to the first page

Family 15 – Father

H6

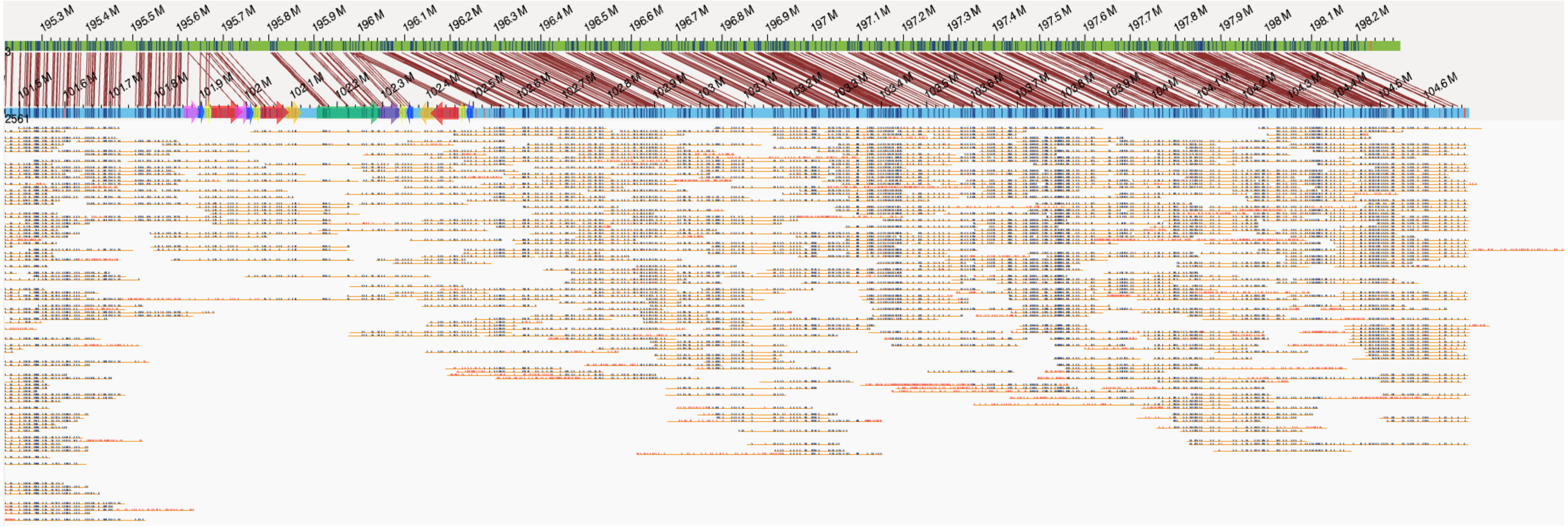

H6

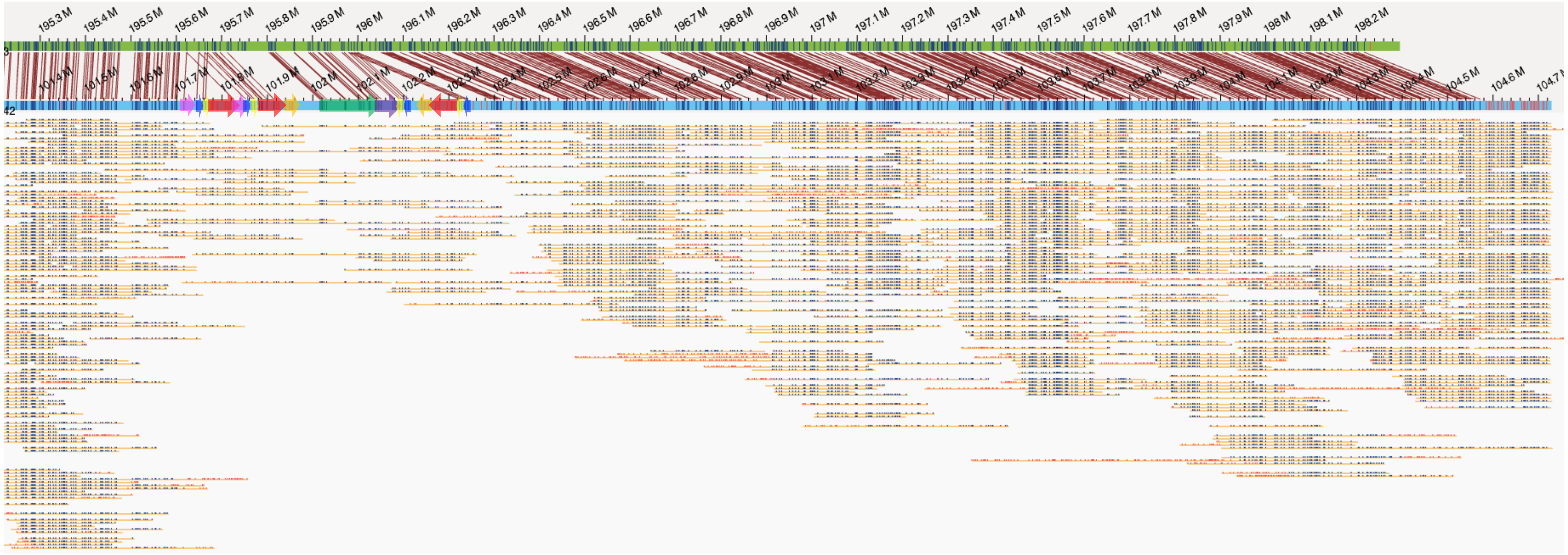

Go to the first page

Family 15 – Father

H2

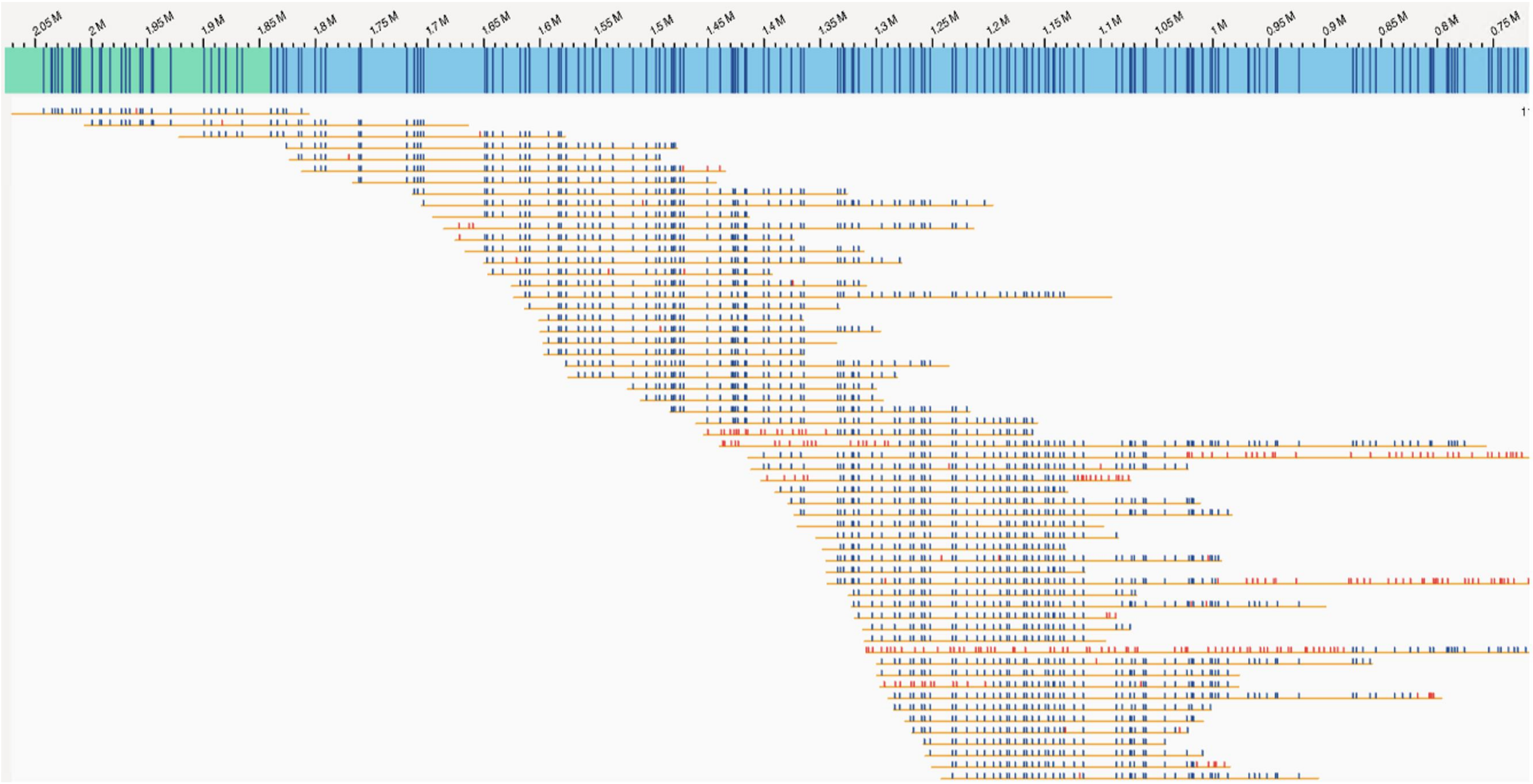

Go to the first page

Family 15 – Mother

H4

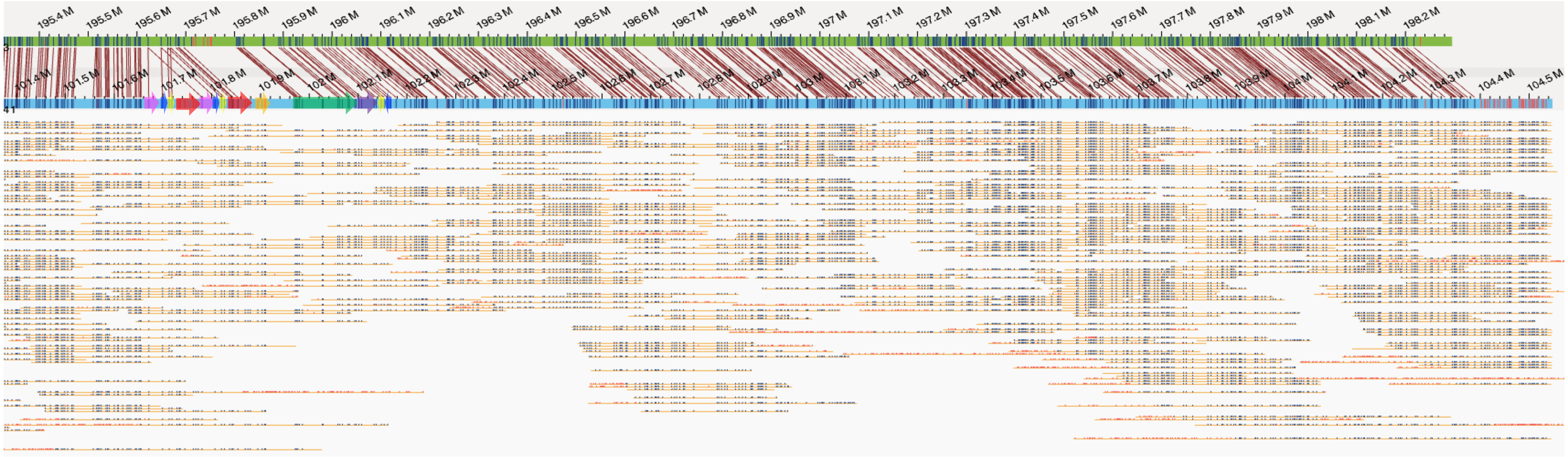

H6

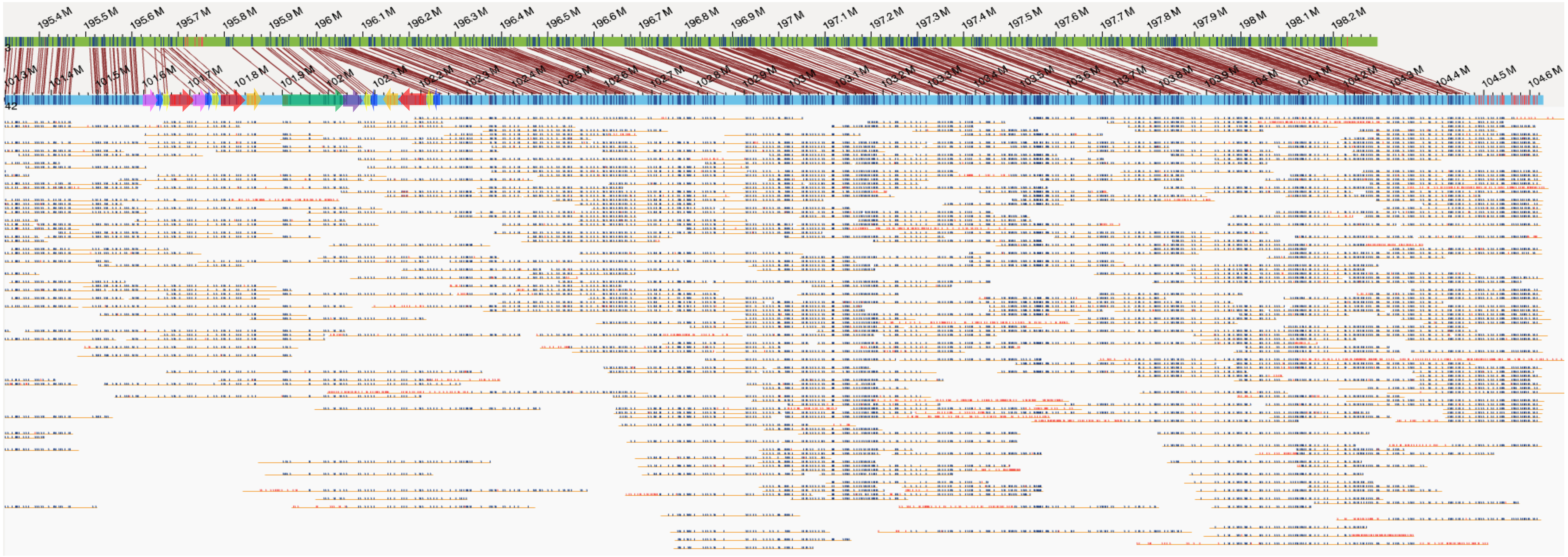

Go to the first page

Family 16 - Trio

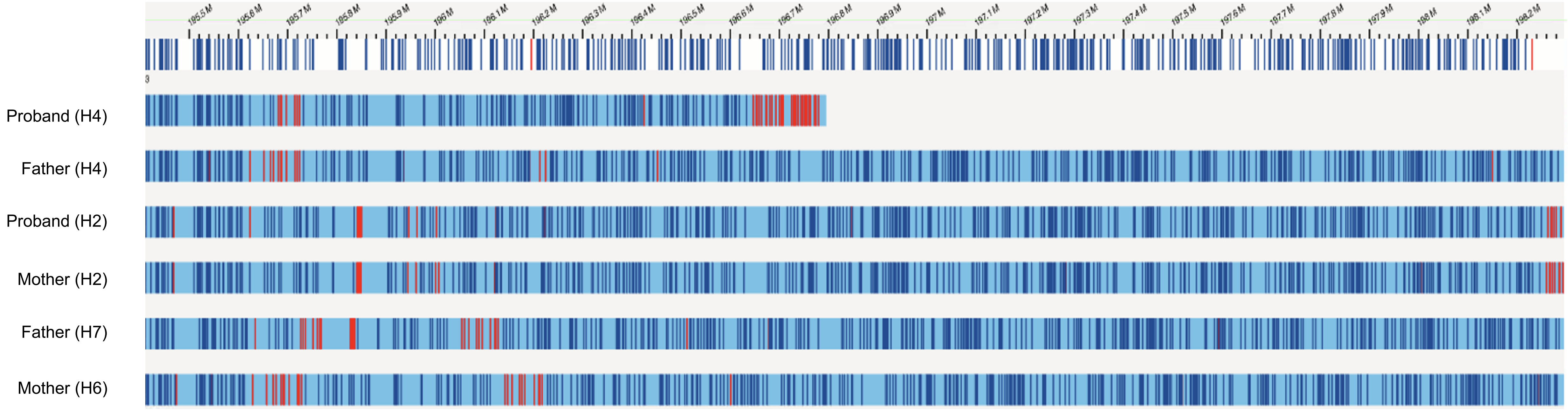

Go to the first page

Family 16 - Proband

H4

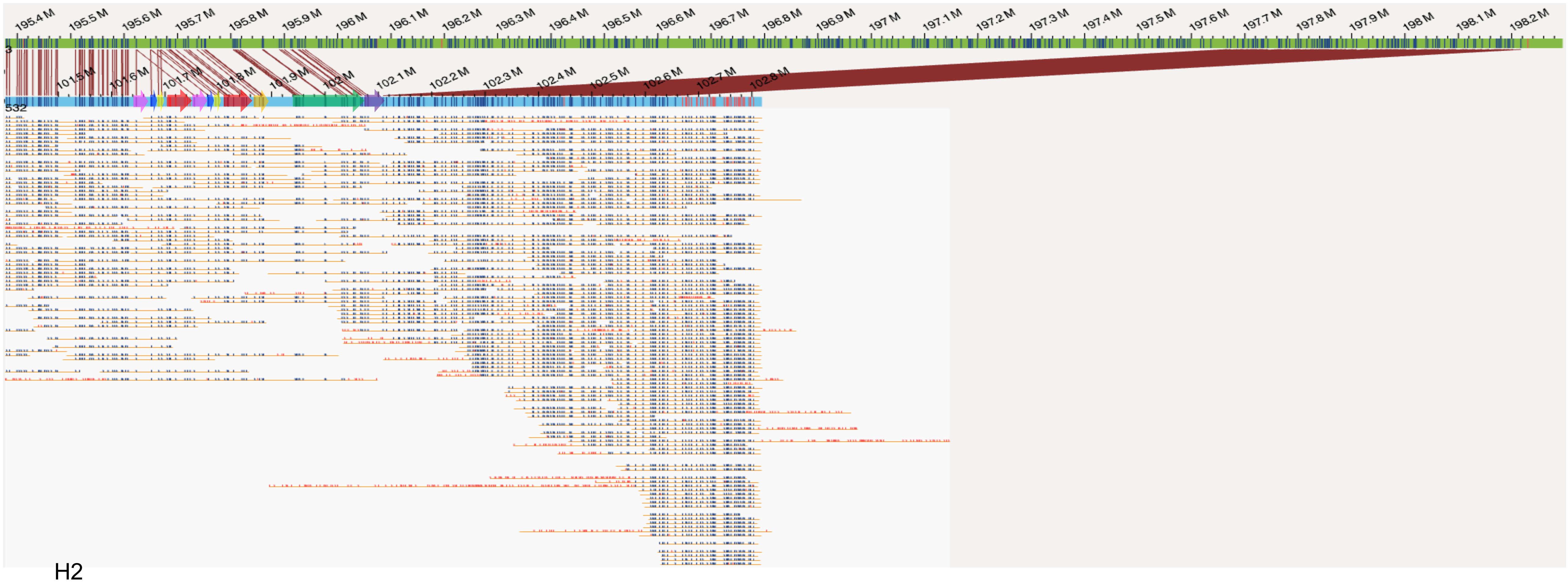

H2

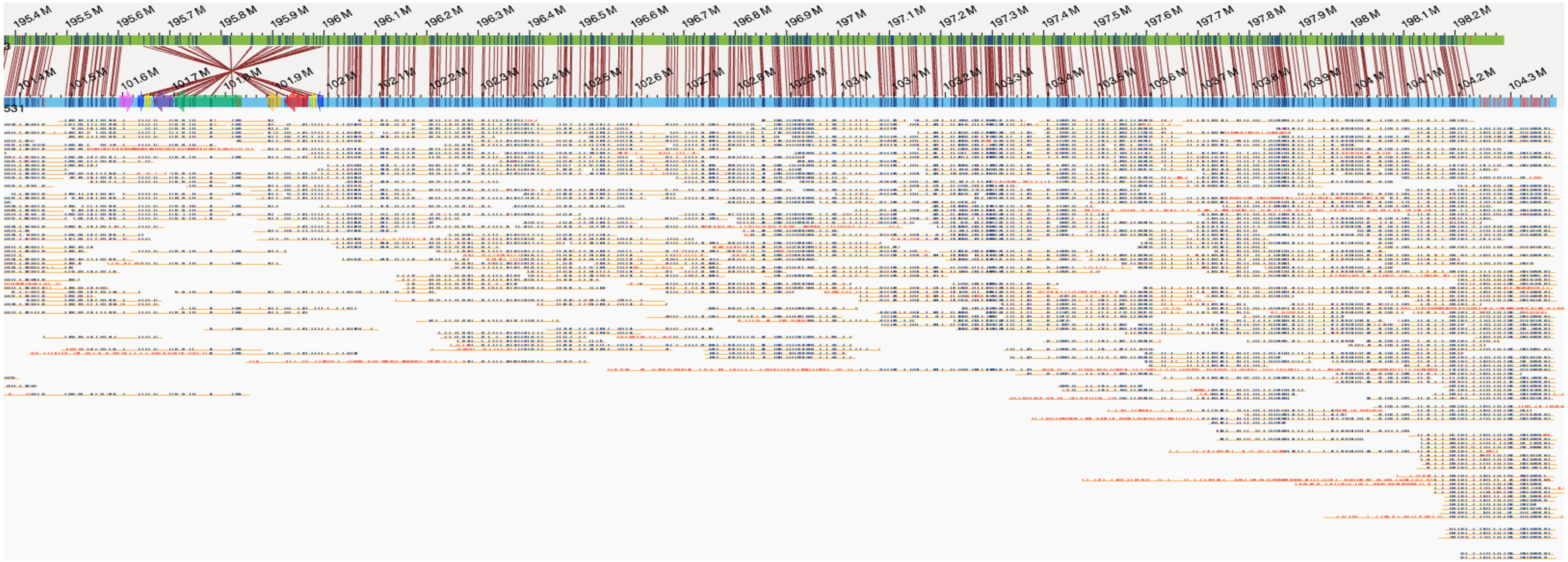

Go to the first page

Family 16 – Father

H7

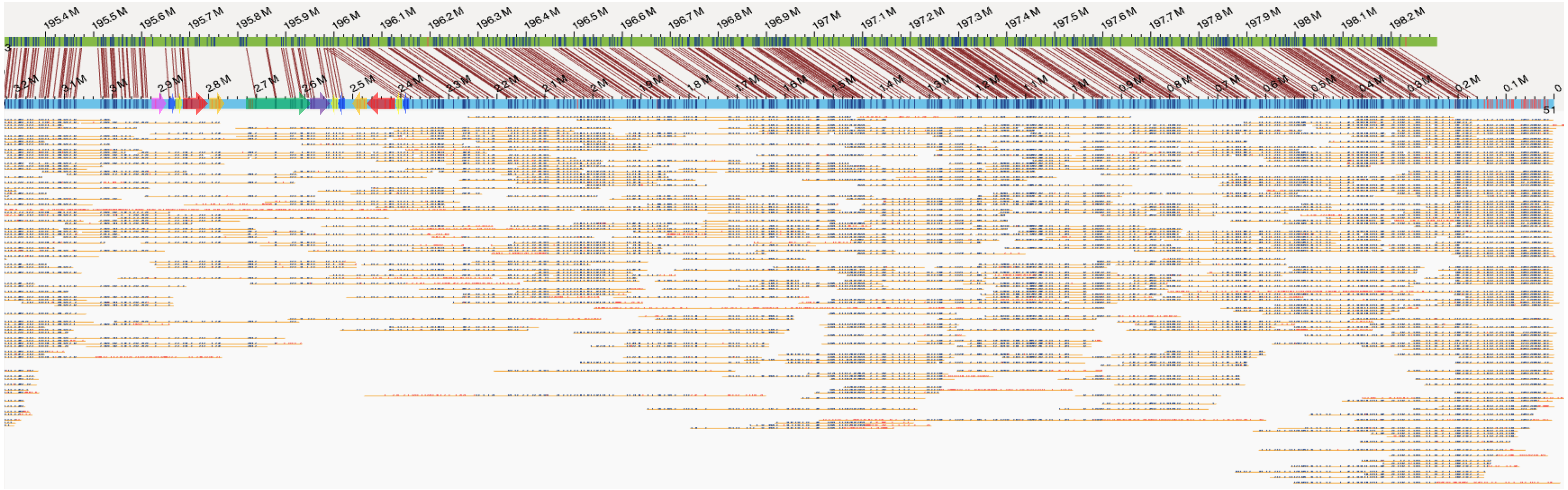

H4

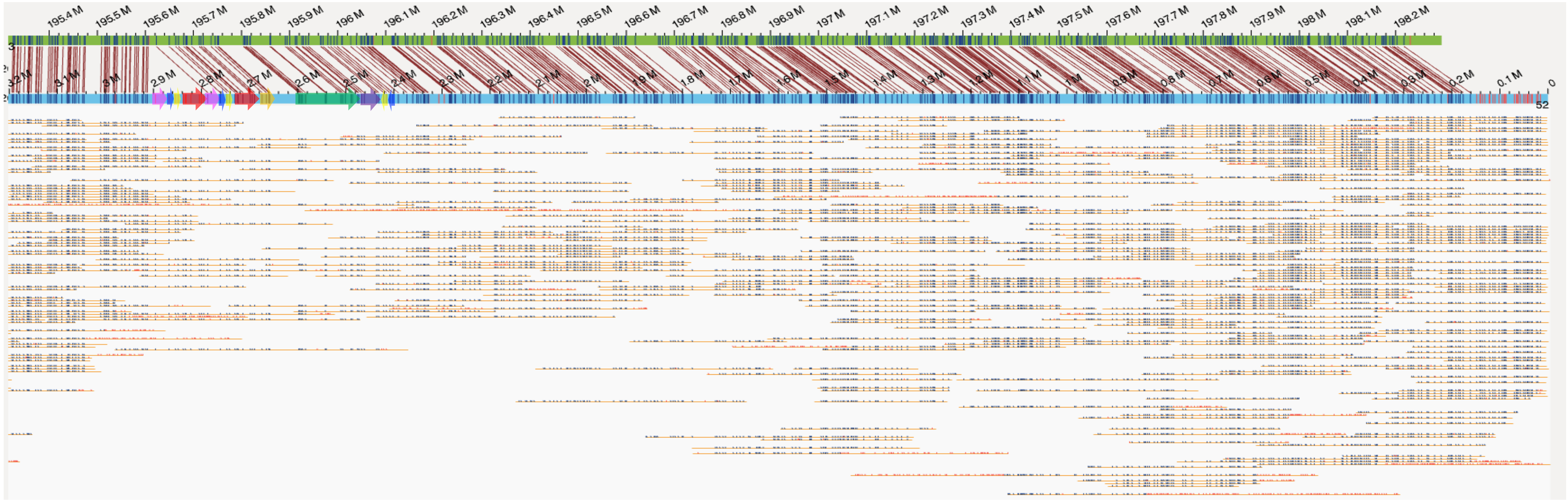

Go to the first page

Family 16 – Mother

H6

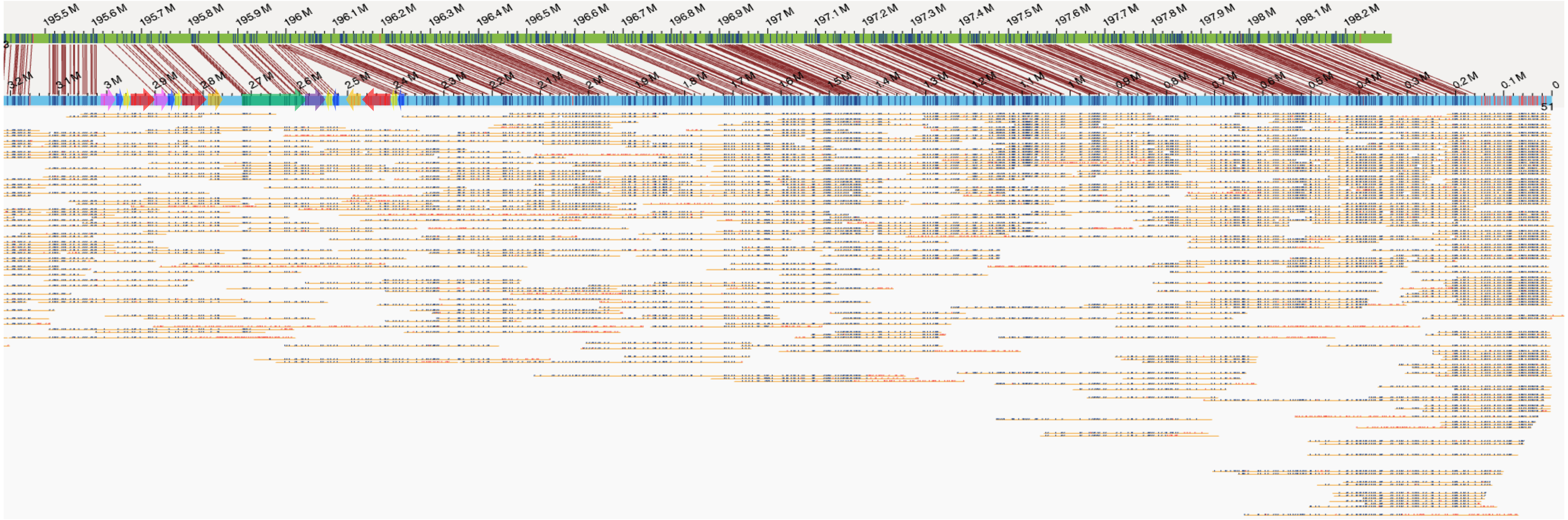

H2

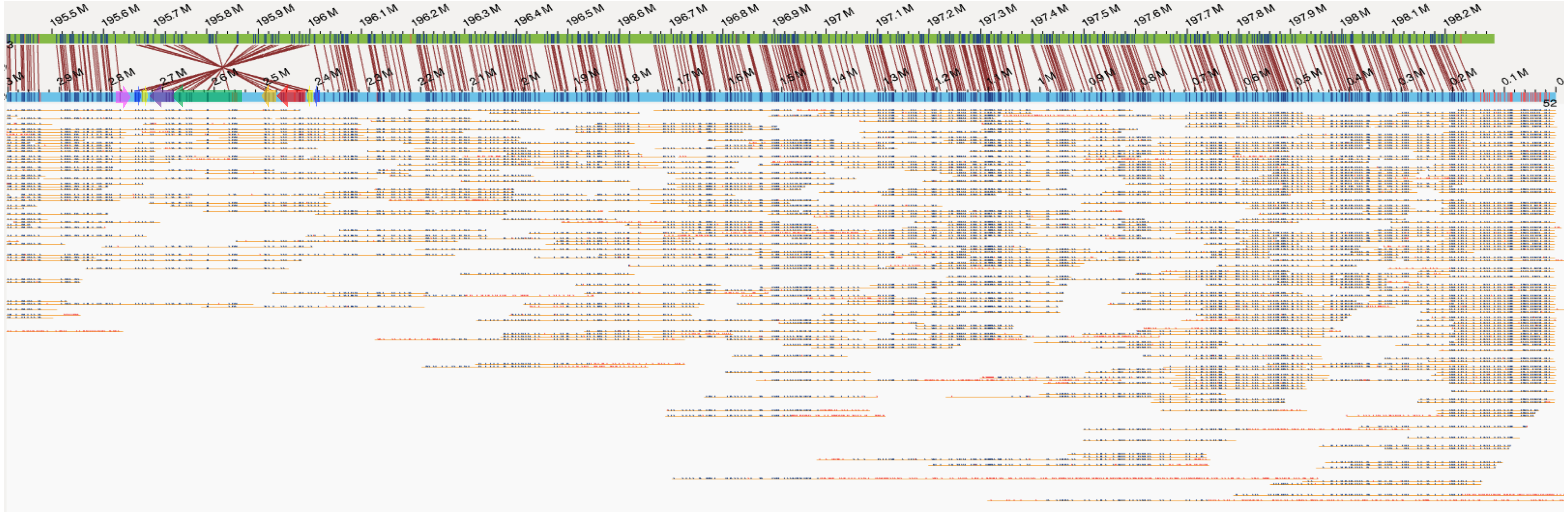

Go to the first page

Family 17 – Proband & Father

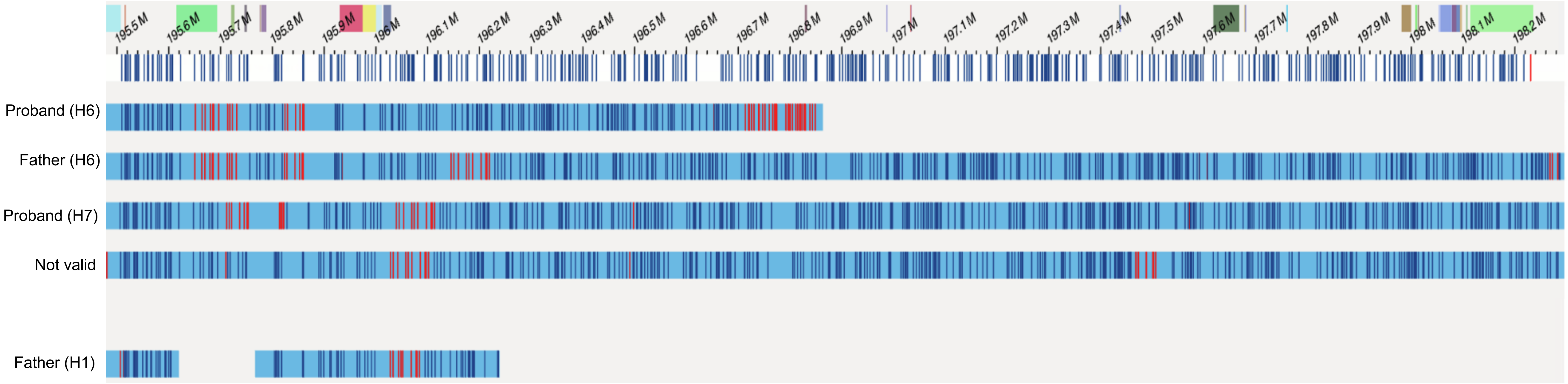

Go to the first page

Family 17 - Proband

H6

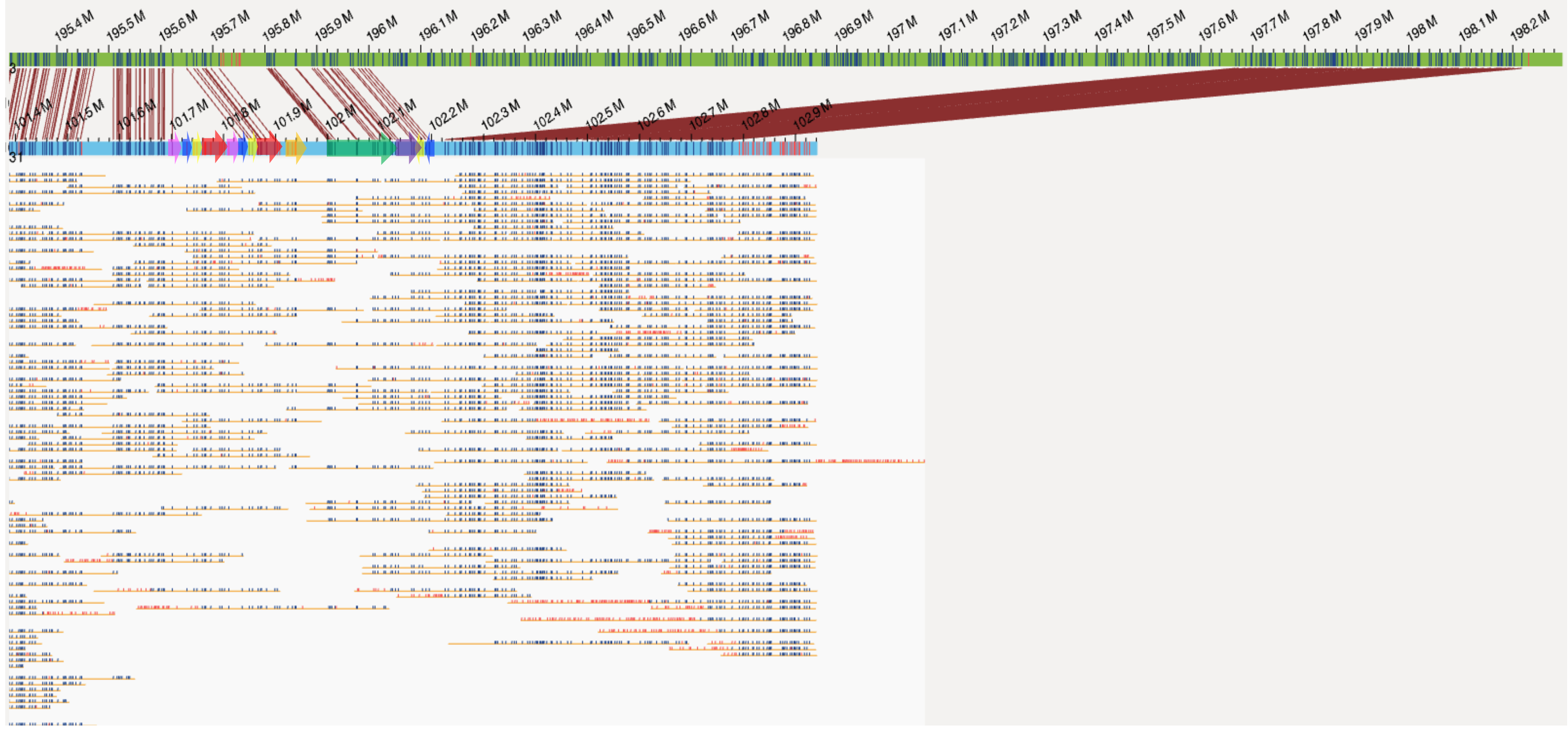

H7

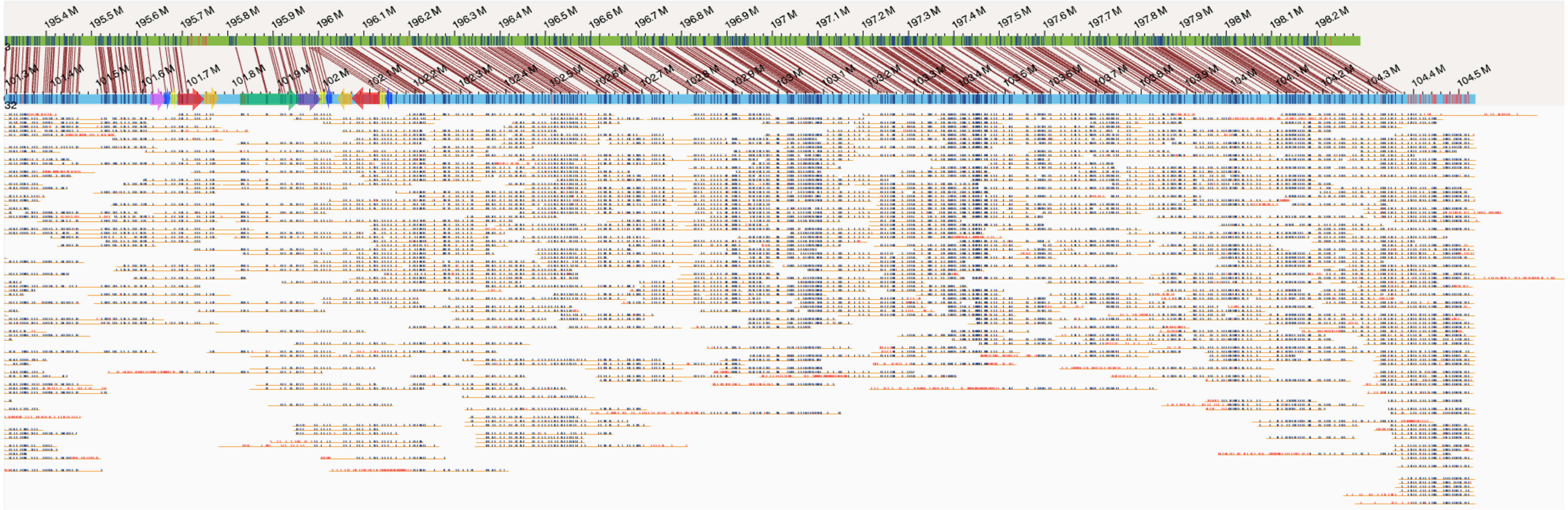

Go to the first page

Family 17 - Father

H1

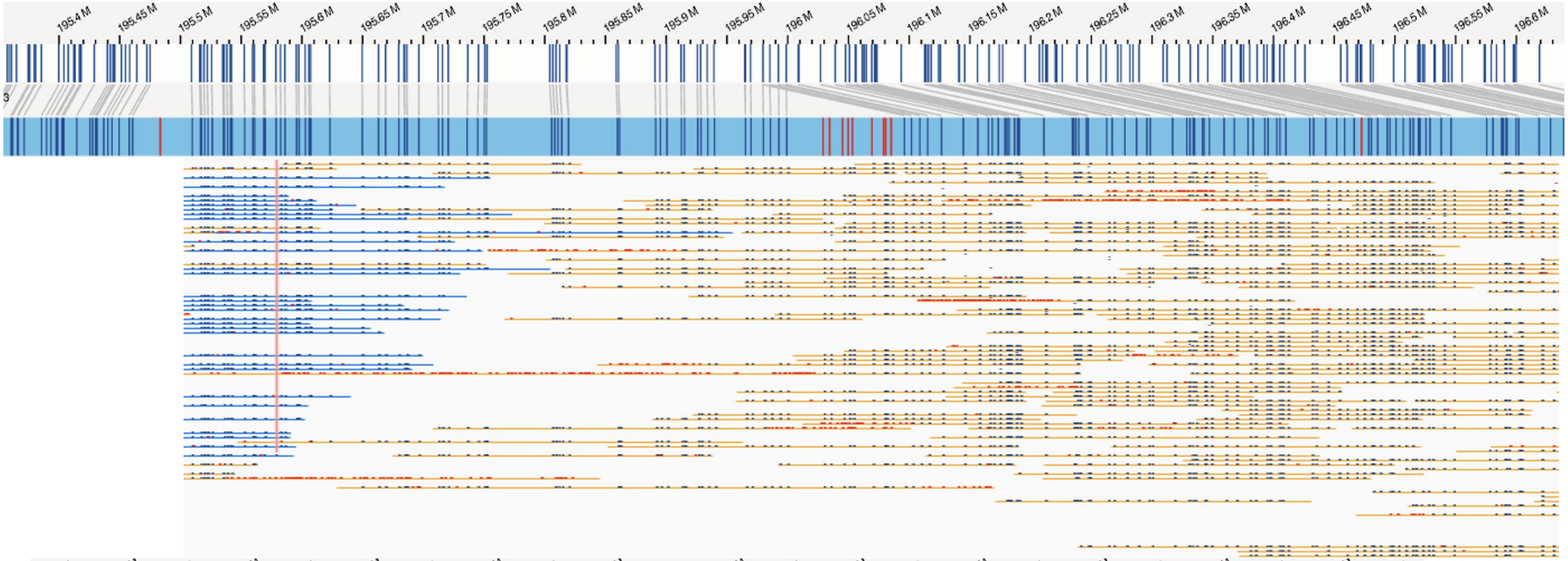

H6

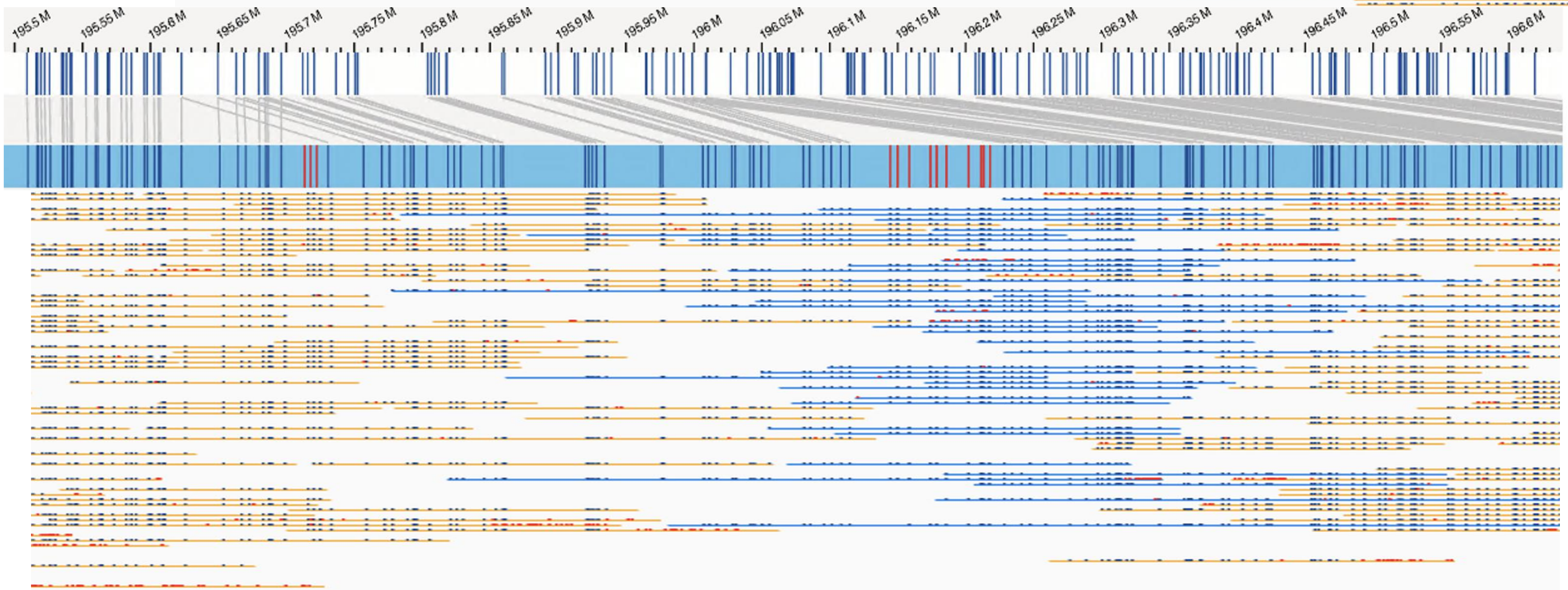

Go to the first page

Family 18 – Proband & Father

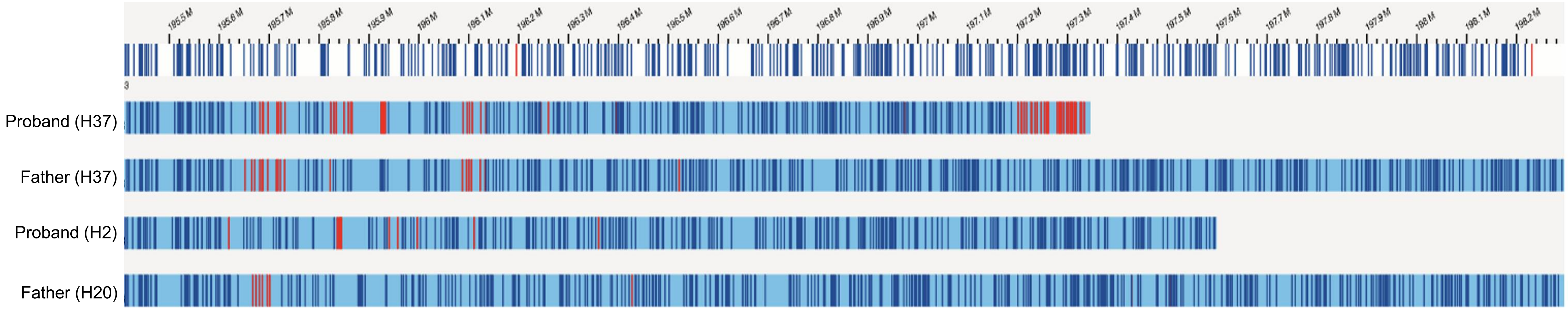

Go to the first page

Family 18 - Proband

H37

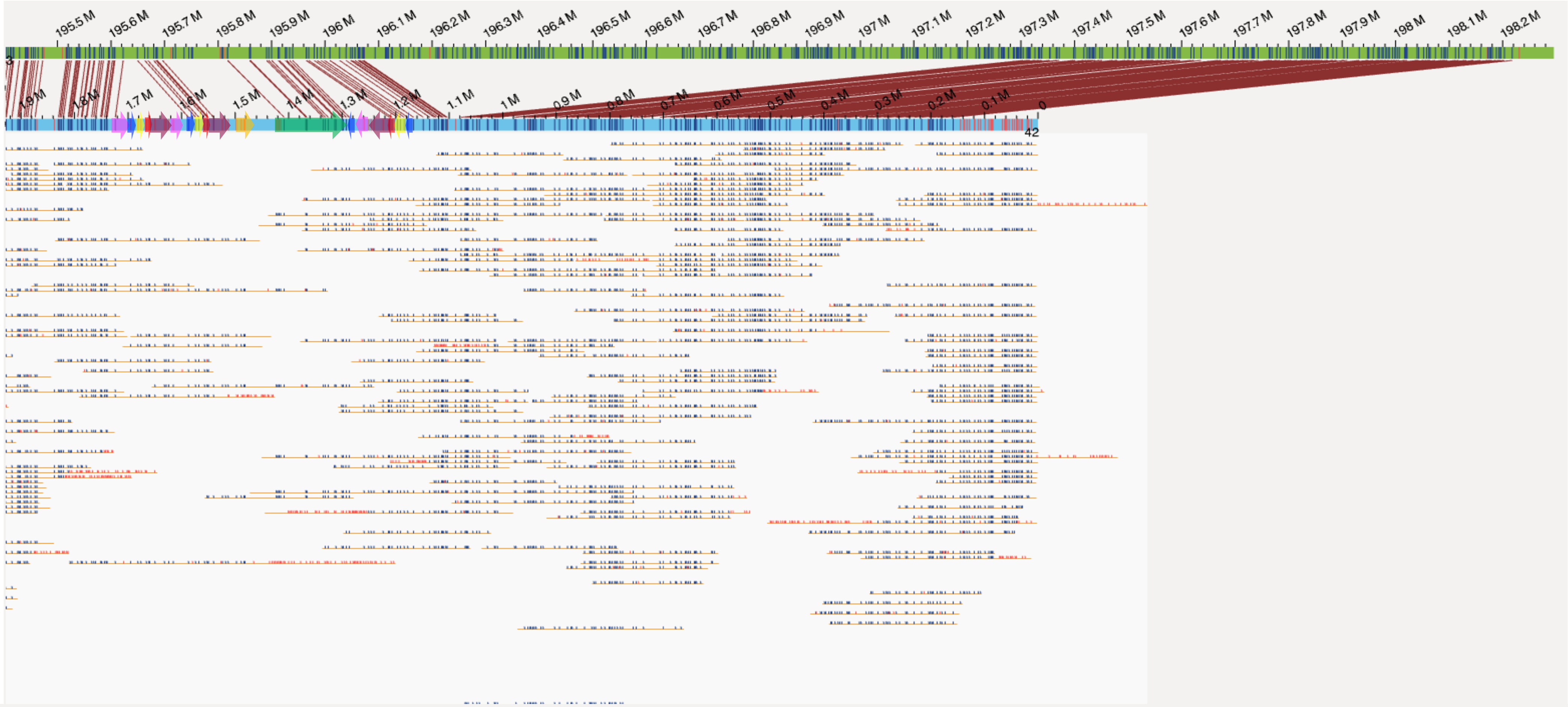

H2

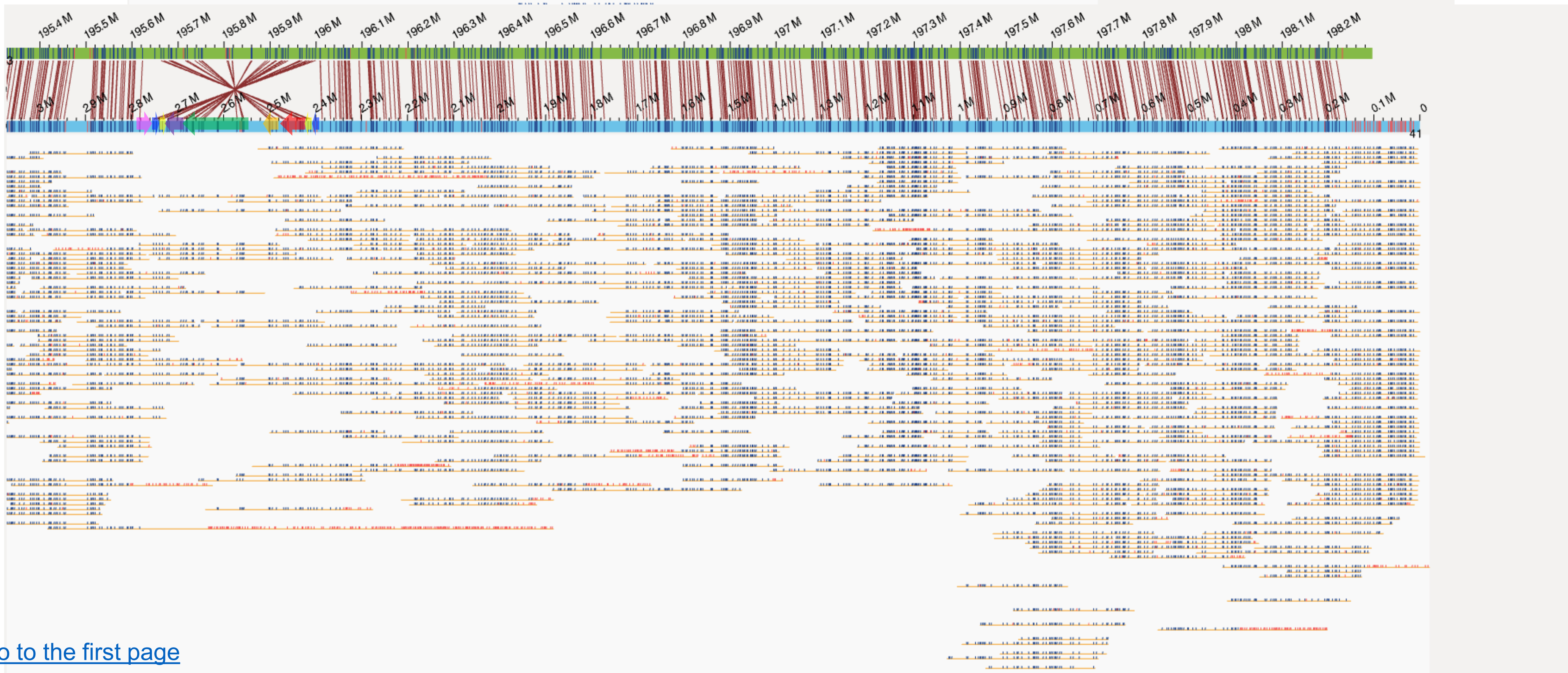

Go to the first page

Family 18 – Father

H20

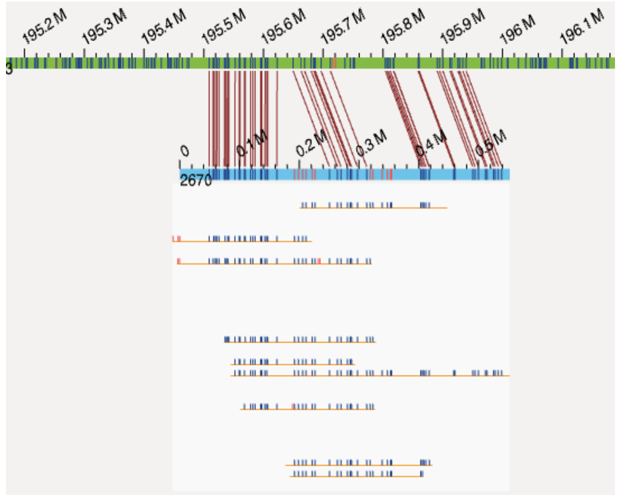

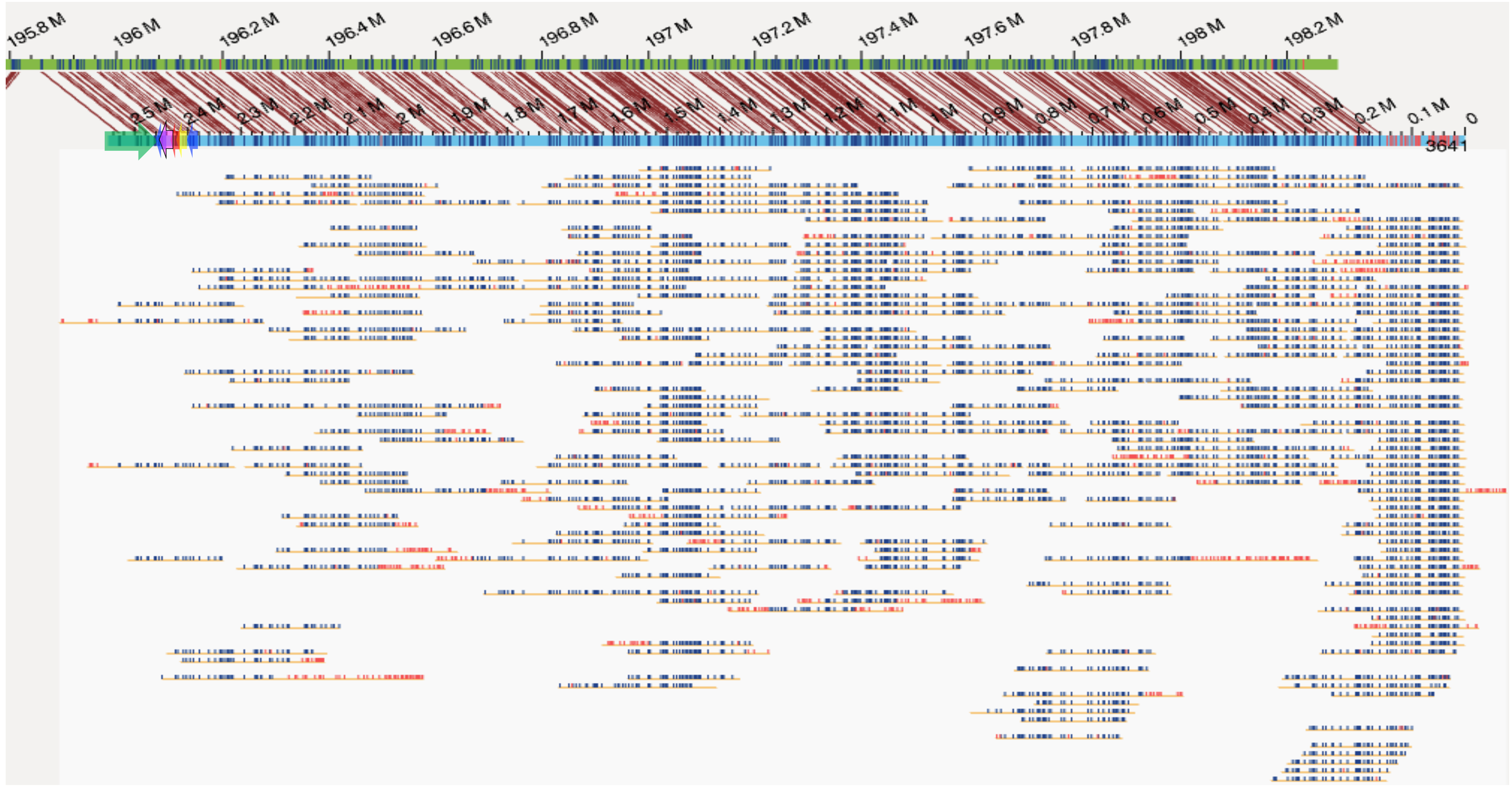

H37

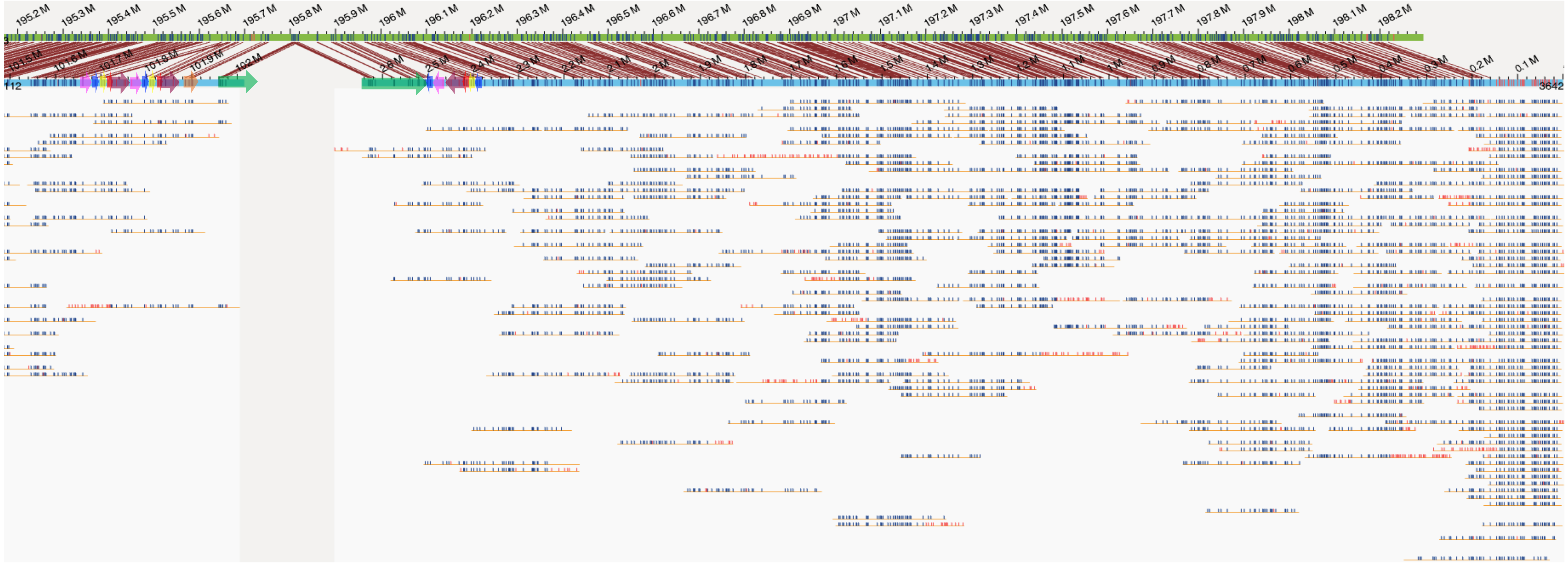

Go to the first page

Family 19 – Proband

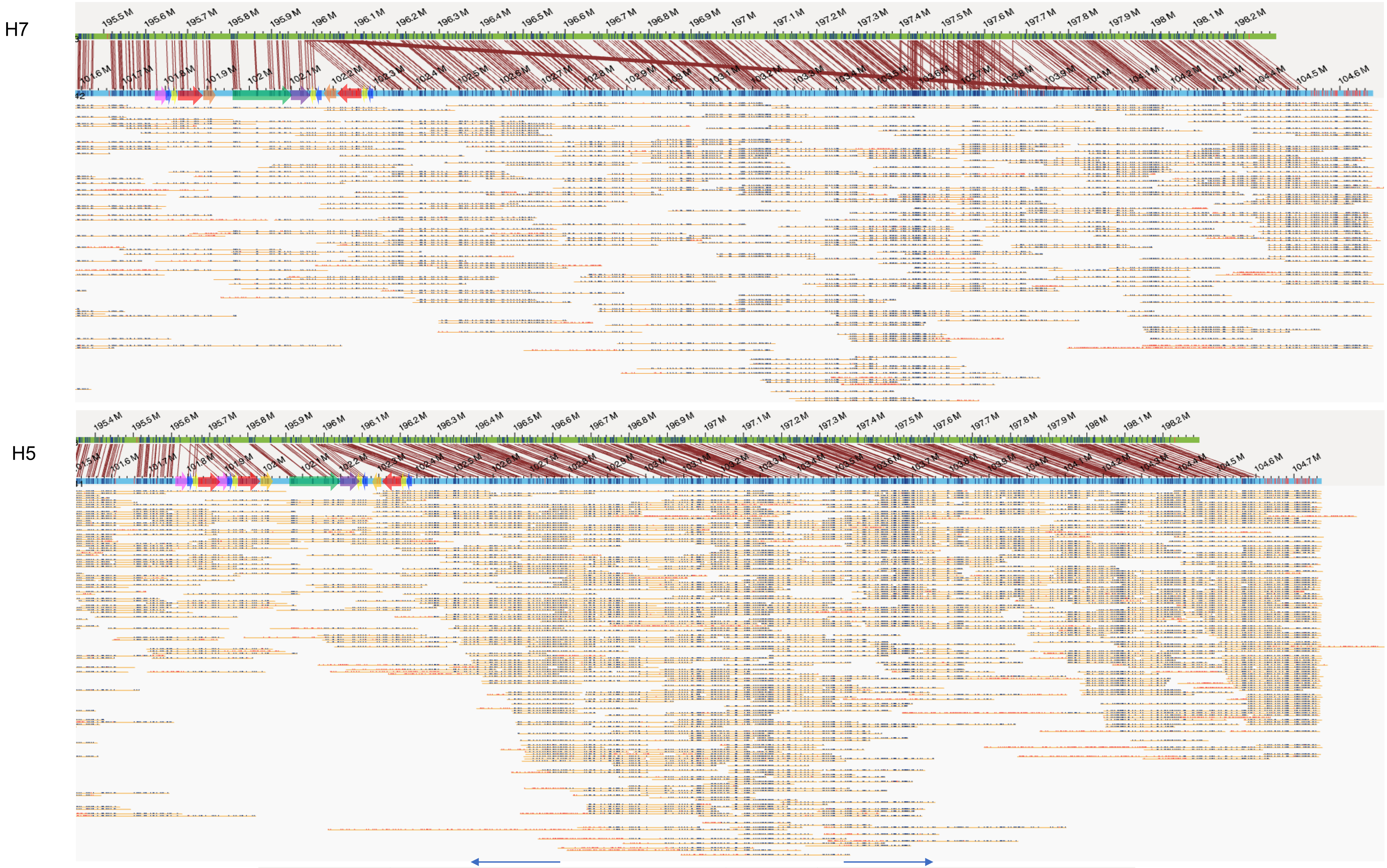

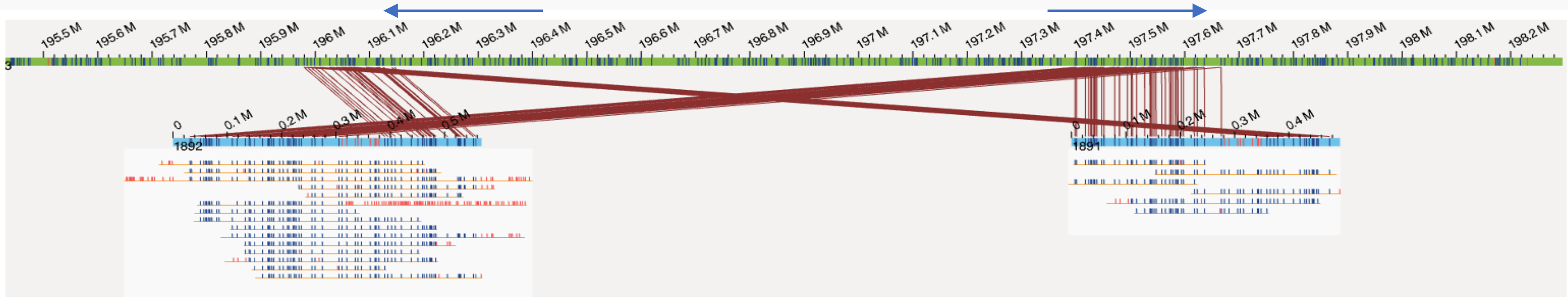

Go to the first page

Family 20 – Deletion Inherited from Father

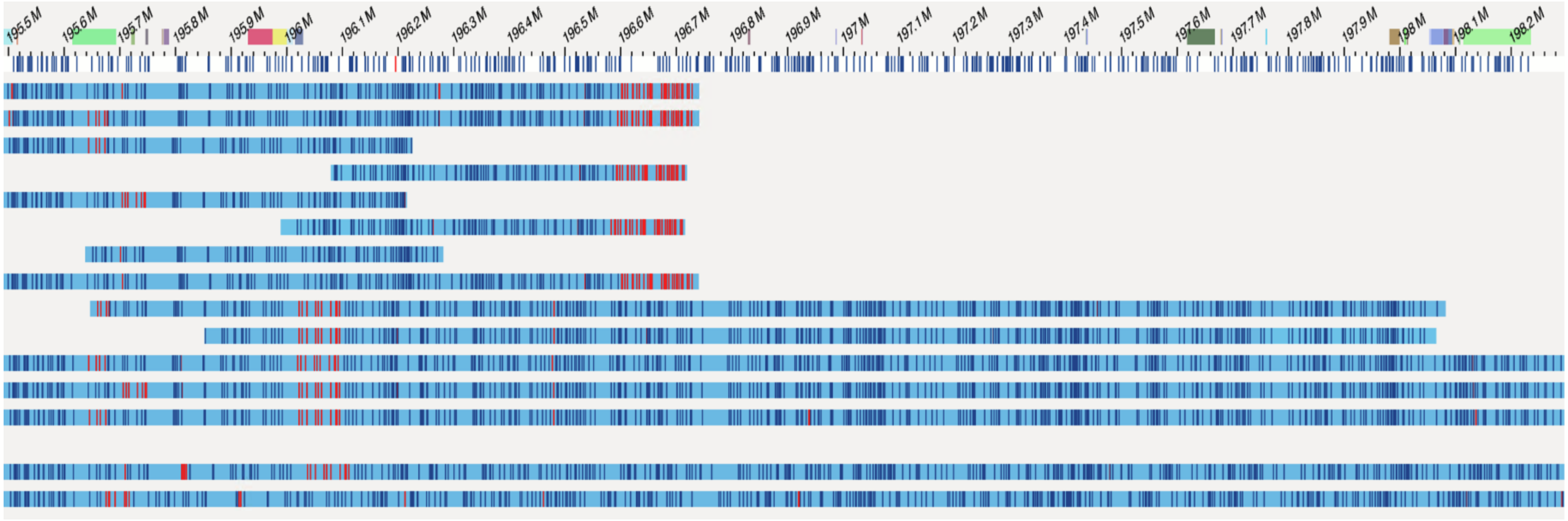

Go to the first page

Family 20 - Proband

NA

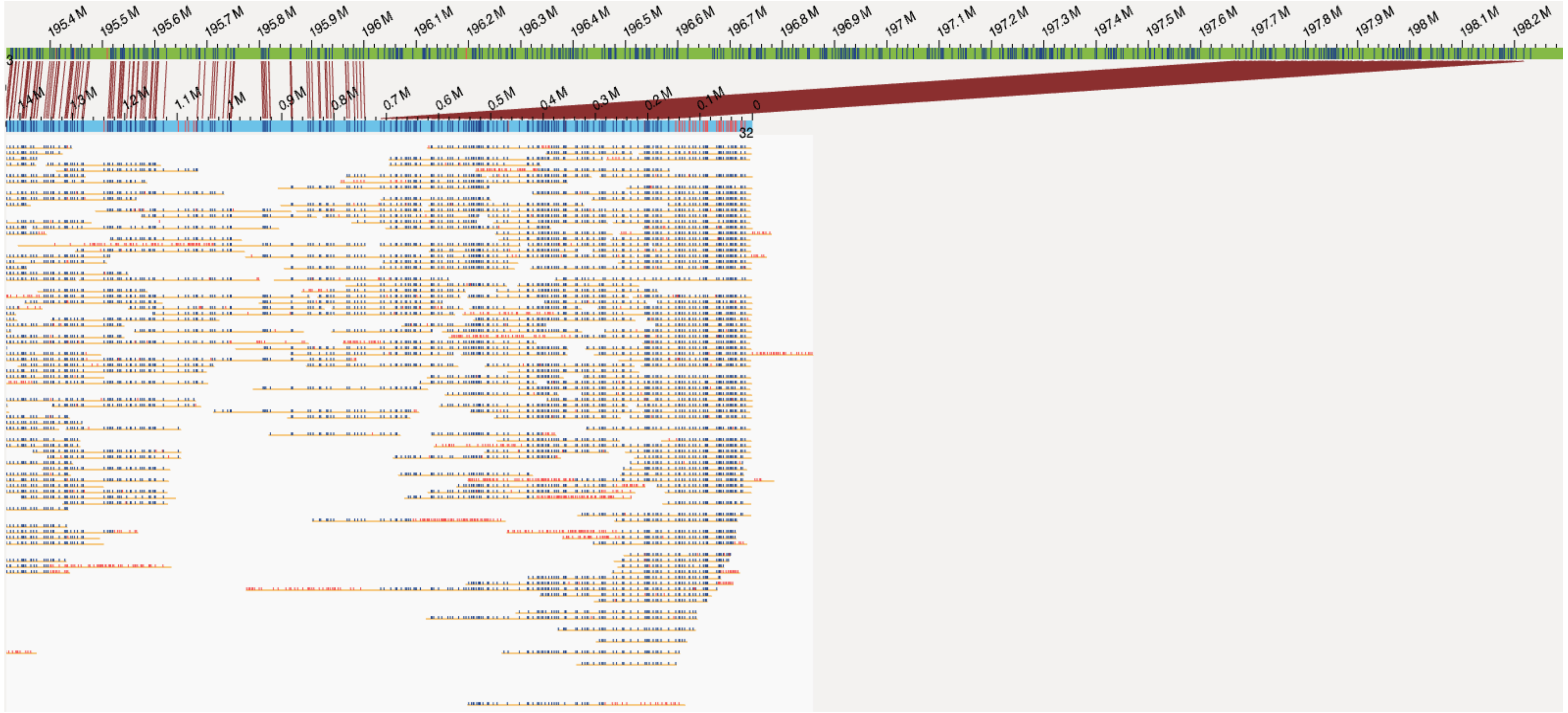

H7

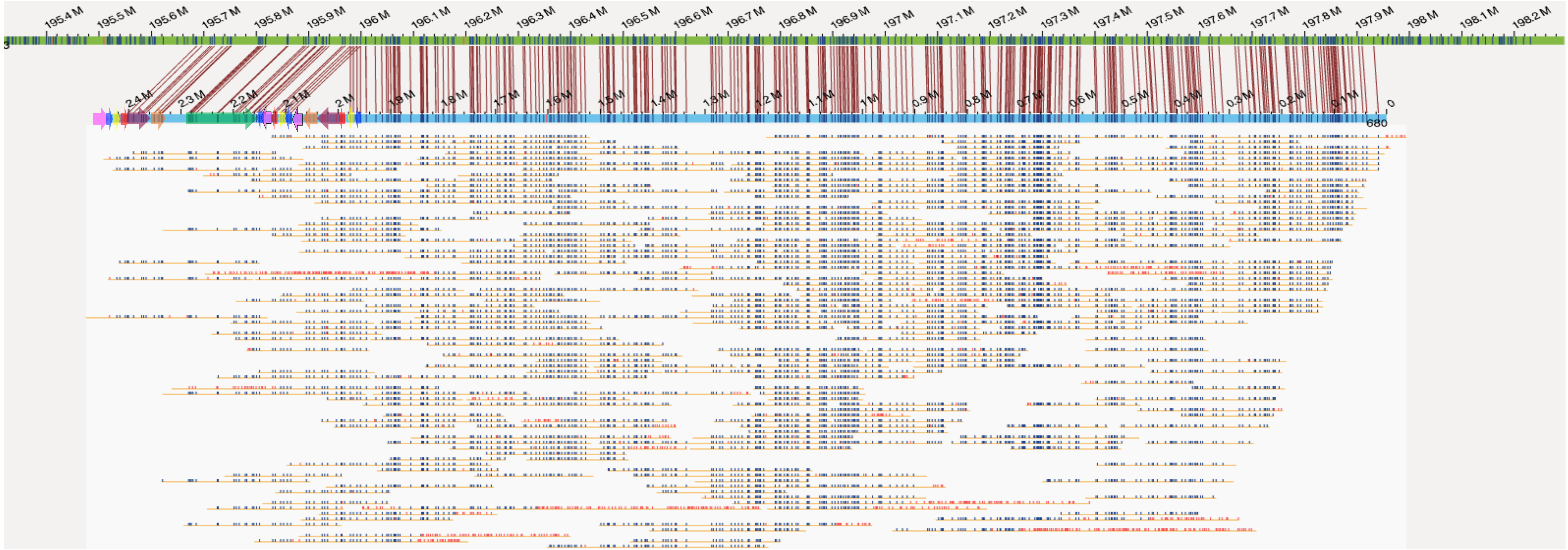

Go to the first page

Family 20 – Sibling

NA

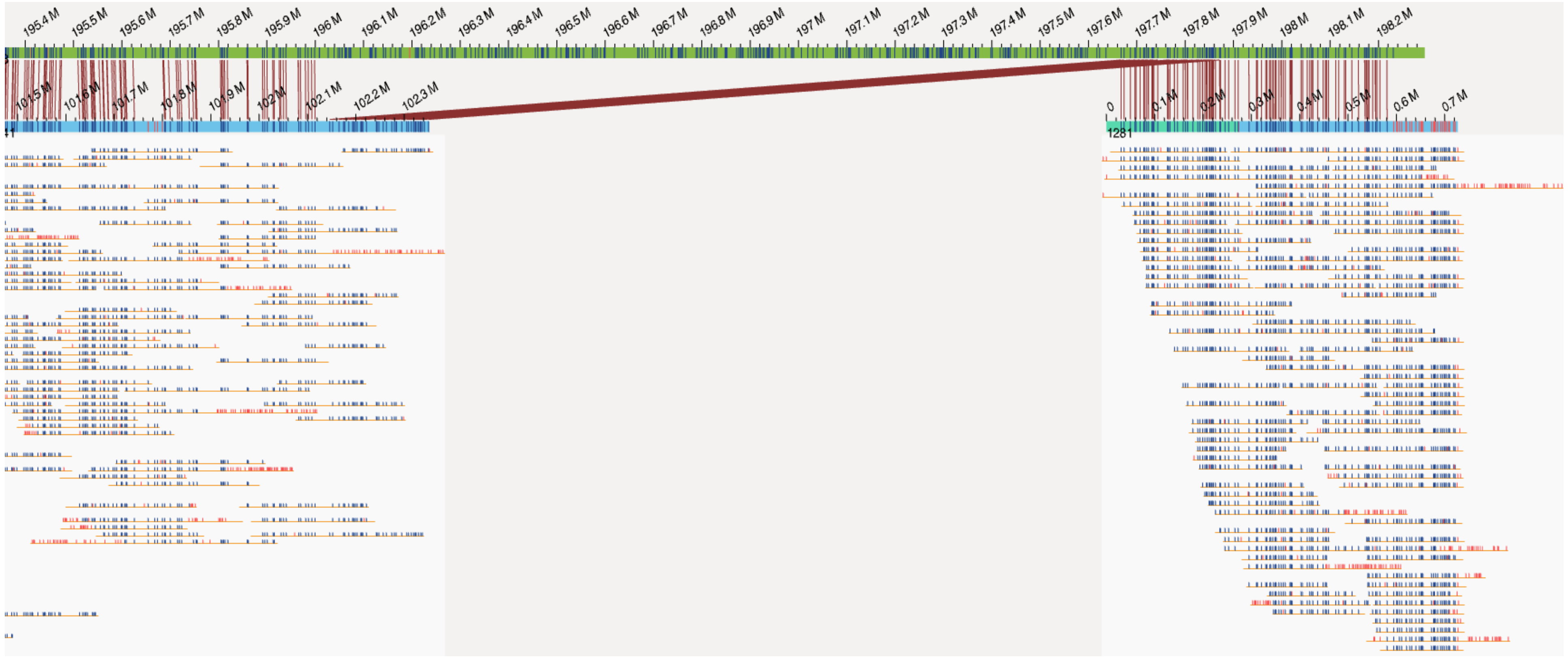

H7

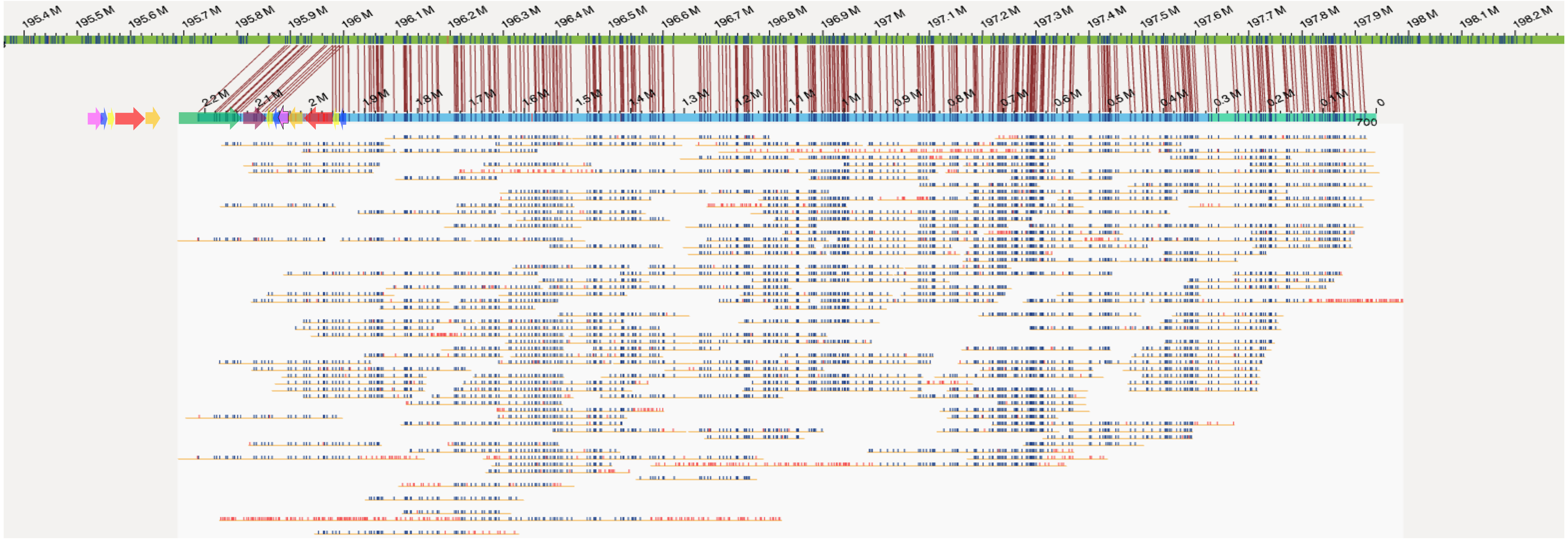

Go to the first page

Family 20 – Sibling

NA

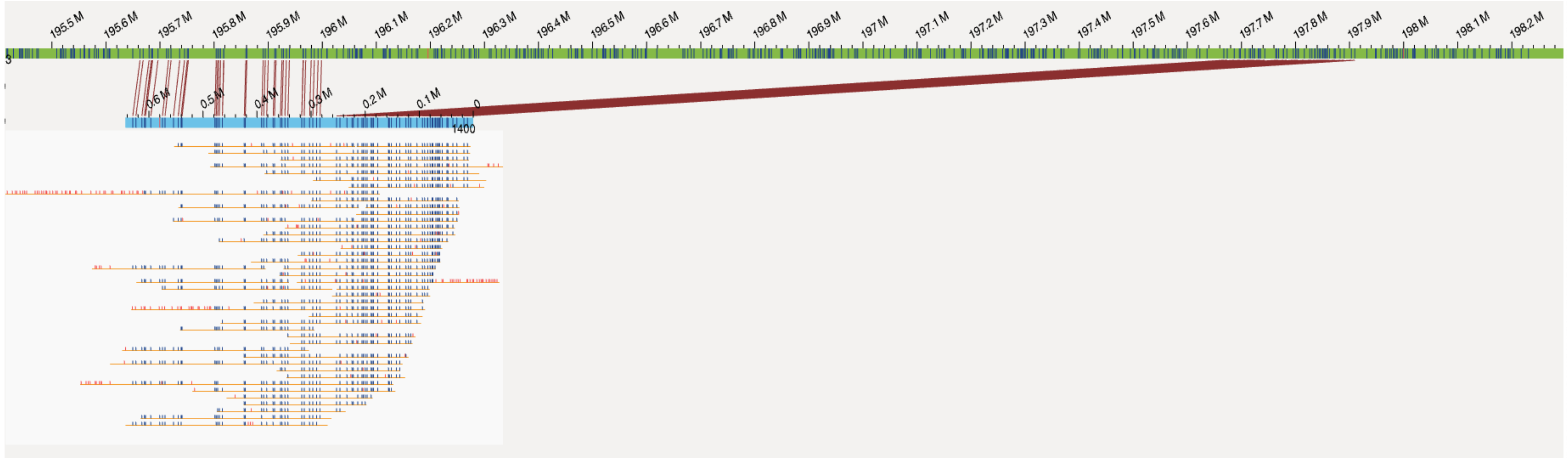

H7

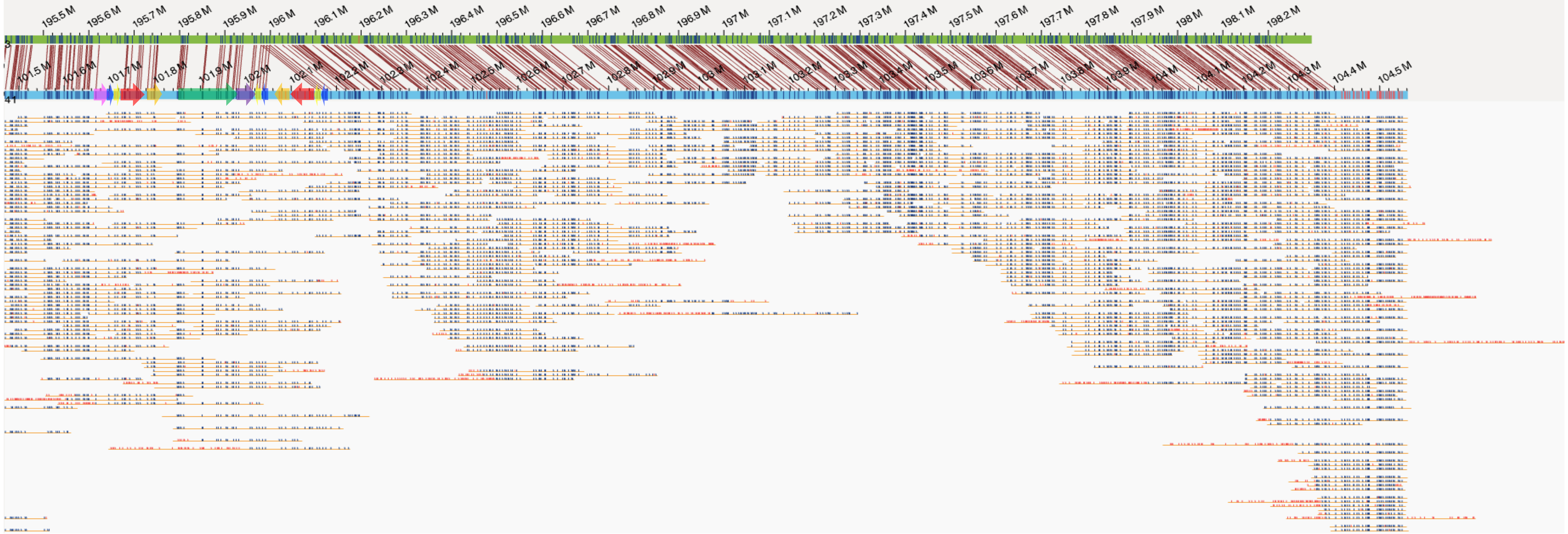

Go to the first page

Family 20 – Father

NA

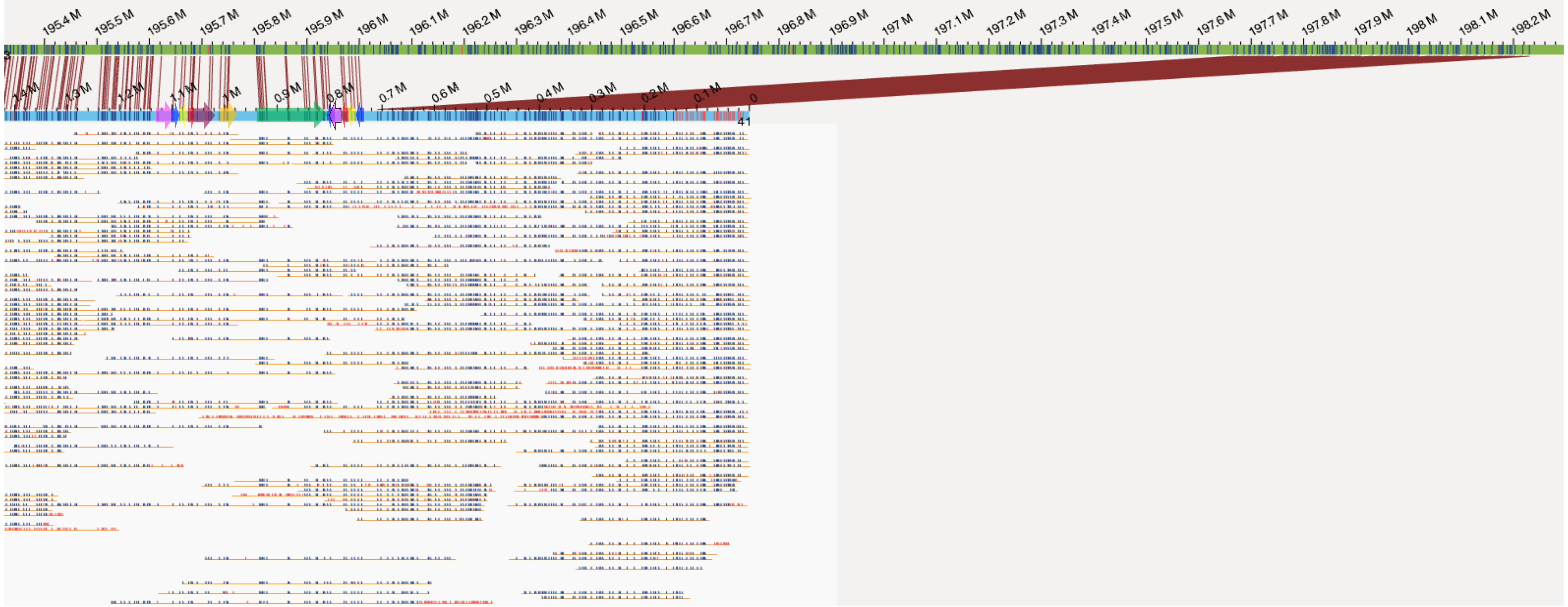

H7

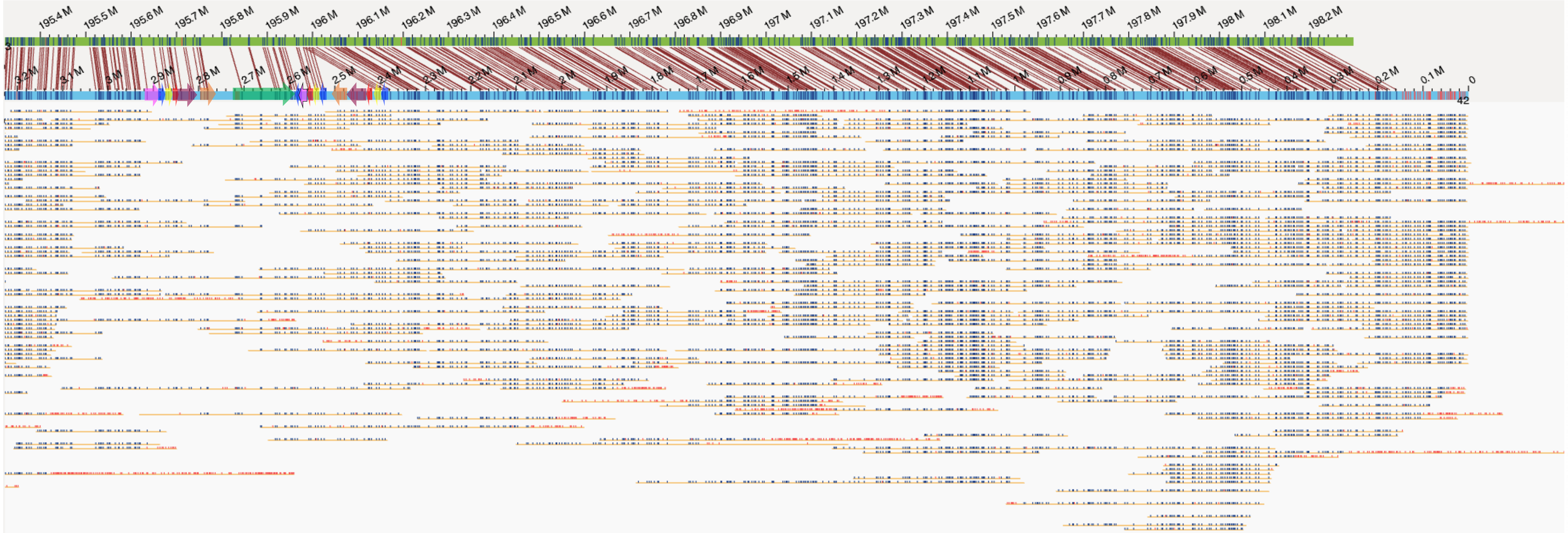

Go to the first page

Family 20 – Mother

H4

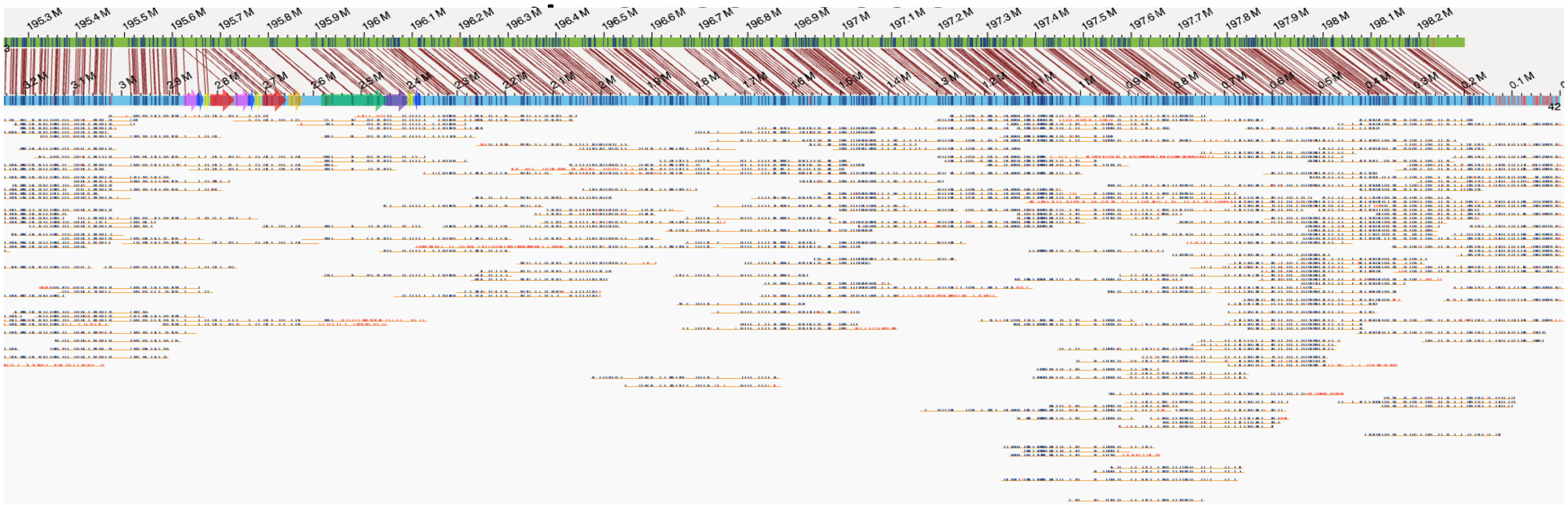

H7

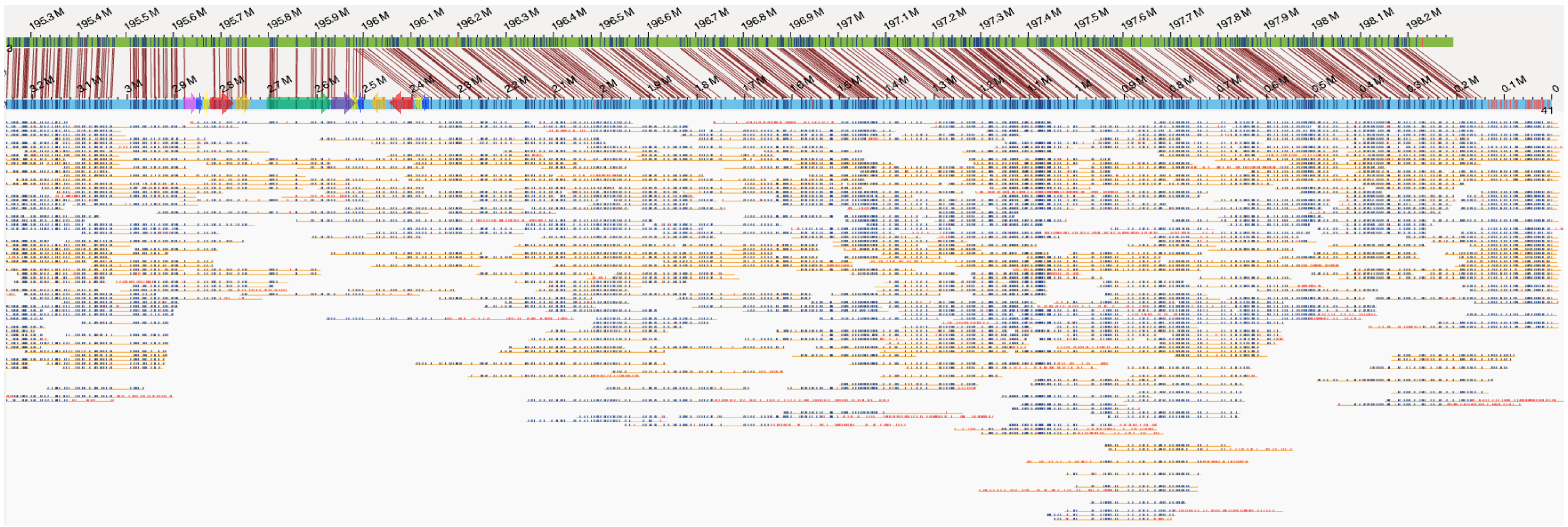

Go to the first page
